# High-throughput micropatterning platform reveals Nodal-dependent bisection of peri-gastrulation–associated versus preneurulation-associated fate patterning

**DOI:** 10.1371/journal.pbio.3000081

**Published:** 2019-10-21

**Authors:** Mukul Tewary, Dominika Dziedzicka, Joel Ostblom, Laura Prochazka, Nika Shakiba, Tiam Heydari, Daniel Aguilar-Hidalgo, Curtis Woodford, Elia Piccinini, David Becerra-Alonso, Alice Vickers, Blaise Louis, Nafees Rahman, Davide Danovi, Mieke Geens, Fiona M. Watt, Peter W. Zandstra

**Affiliations:** 1 Institute of Biomaterials and Biomedical Engineering (IBBME), University of Toronto, Toronto, Ontario, Canada; 2 Collaborative Program in Developmental Biology, University of Toronto, Toronto, Ontario, Canada; 3 Terrence Donnelly Centre for Cellular & Biomolecular Research, University of Toronto, Toronto, Ontario, Canada; 4 Centre for Stem Cells & Regenerative Medicine, King's College London, London, United Kingdom; 5 Research Group Reproduction and Genetics, Faculty of Medicine and Pharmacy, Vrije Universiteit Brussel, Brussels, Belgium; 6 Michael Smith Laboratories, University of British Columbia, Vancouver, British Columbia, Canada; 7 School of Biomedical Engineering, University of British Columbia, Vancouver, British Columbia, Canada; 8 Department of Quantitative Methods, Universidad Loyola Andalucia, Sevilla, Spain; University of Copenhagen, DENMARK

## Abstract

In vitro models of postimplantation human development are valuable to the fields of regenerative medicine and developmental biology. Here, we report characterization of a robust in vitro platform that enabled high-content screening of multiple human pluripotent stem cell (hPSC) lines for their ability to undergo peri-gastrulation–like fate patterning upon bone morphogenetic protein 4 (BMP4) treatment of geometrically confined colonies and observed significant heterogeneity in their differentiation propensities along a gastrulation associable and neuralization associable axis. This cell line–associated heterogeneity was found to be attributable to endogenous Nodal expression, with up-regulation of Nodal correlated with expression of a gastrulation-associated gene profile, and Nodal down-regulation correlated with a preneurulation-associated gene profile expression. We harness this knowledge to establish a platform of preneurulation-like fate patterning in geometrically confined hPSC colonies in which fates arise because of a BMPs signalling gradient conveying positional information. Our work identifies a Nodal signalling-dependent switch in peri-gastrulation versus preneurulation-associated fate patterning in hPSC cells, provides a technology to robustly assay hPSC differentiation outcomes, and suggests conserved mechanisms of organized fate specification in differentiating epiblast and ectodermal tissues.

## Abstract 2

This study describes a method to generate a robust high-throughput micropatterning platform, and uses it to reveal the role played by Nodal signalling in the self-organization of BMP signalling and the consequent fates that arise in micropatterned human embryonic stem cell colonies.

## Introduction

Following implantation, embryos undergo a dramatic transformation mediated by tissue growth, cell movements, morphogenesis, and fate specifications resulting in the self-organized formation of the future body plan [1]. Postimplantation development to the neurula-stage embryo is orchestrated by 2 vital developmentally conserved events called gastrulation and neurulation. Gastrulation is the developmental stage that segregates the pluripotent epiblast into the 3 multipotent germ layers, namely, the ectoderm, the mesoderm, and the endoderm [2–4]. Closely following gastrulation, the ectoderm undergoes further fate specification resulting in the patterned neural plate, neural plate border, and non-neural ectoderm regions thereby setting the stage for the onset of neurulation [5–9]. As neurulation proceeds, morphogenetic changes in these tissues result in the formation of the neural tube, the neural crest, and the epithelium, respectively [10]. Initiation of the morphogenetic restructuring of the epiblast and the ectoderm occurs because of self-organized gradients of signalling molecules called morphogens, and morphogens belonging to the transforming growth factor beta (TGFβ) superfamily, such as bone morphogenetic proteins (BMPs) and Nodal, play vital roles in these developmental stages.

Two biochemical models, reaction-diffusion (RD) and positional information (PI), have strongly influenced our mechanistic understanding of self-organized fate specification during embryogenesis. The RD model describes how a homogeneously distributed morphogen can self-organize into a signalling gradient in a developing tissue because of the presence of an interaction network between the morphogen and its inhibitor, both of which are hypothesized to be diffusible molecules albeit with differential diffusivities [11–13]. Recent interpretations of RD have proposed that higher order (>2 molecules) network topologies can also underlie this self-organization [14]. The PI model describes how fate patterning can occur in a developing tissue because of an asymmetric morphogen distribution. The classical version of this paradigm hypothesized that the cells in the developing tissue sense the morphogen concentration in their immediate vicinity and acquire fates according to a threshold model [15,16]. Recent studies have updated this interpretation of the PI model and suggest that fates are acquired as a function of both the morphogen concentration and time of induction [17,18]. Although both RD and PI have been incredibly valuable in facilitating our comprehension of how developmental fates arise in a self-organized manner, these models are typically studied in individual signalling pathways. How multiple signalling pathways may work in concert to execute the rules specified by either RD or PI are not well understood.

Studying postimplantation developmental events, like gastrulation and neurulation, directly in human embryos would unequivocally provide the most reliable interpretations of human development. Although valuable progress has been made of late in culturing human blastocysts in vitro [19,20], ethical concerns preclude their maintenance beyond 14 days—prior to the onset of gastrulation. On the other hand, recent studies on in vivo human development have provided incredible insight into human development well into the fetal stages [21]. However, these studies are performed on specimens acquired from terminations or abortions that are typically accessible after the stages of gastrulation and neurulation have already transpired. Consequently, investigation of the mechanisms underpinning early postimplantation human embryonic development directly in human embryos is currently not possible. Nevertheless, the ability of stem cells to organize into structures in vitro that mimic aspects of postimplantation human development when provided appropriate biophysical and biochemical cues is well established [22–27]. We and others have used human pluripotent stem cells (hPSCs) to demonstrate that BMP4 treatment of geometrically confined hPSC colonies recapitulate aspects of human peri-gastrulation–like organized fate patterning [24–26]. Although stem cell–derived in vitro constructs are indisputably an incomplete representation of embryos, they can serve to provide insights into some cell organizational events that occur during the critically important postimplantation developmental stages.

In addition to providing insight into cell organizational aspects of early gastrulation-like behaviour, in vitro models of human development are also of great value to the field of regenerative medicine. This is because the ability of these models to specify early developmental cell fates highlights their suitability for characterization of differentiation propensities of hPSC lines. It is well known that hPSC lines—whether they are derived from embryos or from reprogrammed somatic cells—have an inherent bias to differentiate toward specific lineages [28–30]. Consequently, assays that can characterize different hPSC lines to identify these biases are of crucial importance to the field of regenerative medicine. Given the significance of developing approaches to achieve this goal, multiple assays have been established to address this need. The most prominent of these are assays like the teratoma assay [31], the scorecard assay [32], and the pluritest [33]. Although each of these approaches have their benefits, they are either lengthy, tedious and expensive (teratoma and scorecard), or do not directly measure differentiation of the hPSC lines (pluritest). In contrast, the peri-gastrulation–like assay provides a quantitative measure of the generation of lineage-specific fates, is rapid and inexpensive in comparison to approaches like the teratoma assay. In addition, it is readily amenable to high-content screening. However, to capitalize on the capabilities of this assay, we require a robust micropatterning platform that readily enables high-content studies. Conventional approaches to establish micropatterning platforms have employed techniques like micro-contact printing (μCP) [30,34–36] or other soft-lithography approaches [37–39]. In fact, we have previously reported a high-throughput μCP platform that enables geometric confinement in 96-well microtiter plates [30]. However, such approaches require a manual step of stamping the extracellular matrix (ECM) proteins to transfer the adhesive ‘islands’ onto the substrate of choice (glass, tissue culture polystyrene, etc.), which can result in variability in the patterning efficiency and fidelity between experiments and between users. Additionally, they also employ soft-lithography based protocols that require costly equipment and access to clean rooms, which is detrimental to their broad utility. In contrast, techniques that employ the use of Deep UV (<200 nm) light to photo-oxidize Polyethylene Glycol (PEG)-coated substrates offer an attractive alternative to establish robust platforms that can enable high-content screening of hPSC lines [40,41].

Here, we report characterization of a high-content platform to produce microtiter plates that allow robust geometric confinement of a variety of adherent cell types at single-cell resolution. Employing this platform, we tested the response of a panel of hPSC lines to a previously reported peri-gastrulation-like assay [24] and observed significant variability in the induction of the Brachyury (BRA)-expressing region between the lines. To probe the emergent differentiation trajectories of hPSC lines, we assessed their differentiation-associated gene expression profiles and found a switch-like response in the up-regulation of gene profiles associated with either gastrulation or preneurulation. This switch in gene expression showed a strong association with Nodal signalling; hPSC lines that exhibited higher levels of a gastrulation-associated gene expression profile also up-regulated Nodal signalling, and those that exhibited a higher preneurulation-associated gene expression profile down-regulated Nodal signalling. We further validated this observation by inhibiting Nodal signalling in an hPSC line that induces gastrulation-like responses and validated the Nodal-dependent switch of gastrulation versus preneurulation-associated gene expression. In addition, we report that geometrically confined hPSC colonies induced to differentiate in the presence of BMP4 and a Nodal inhibitor undergo an RD-mediated organization of phosphorylated SMAD1 (pSMAD1) activity and PI-mediated fate patterning into compartments that express markers like transcription factor AP2-alpha (TFAP2A), SIX homeobox 1 (SIX1), Orthodenticle Homeobox 2 (OTX2), and GATA binding factor 3 (GATA3) indicative of differentiation toward ectodermal progenitors. We further demonstrate the ability of these progenitor regions to induce marker expression of the definitive fates of the respective compartments. Our findings provide insight into how hPSCs process information from morphogen inputs like BMP and Nodal to generate early developmental fates as output.

## Results

### A high-throughput platform for screening studies of geometrically confined cell colonies

**Fig 1:**
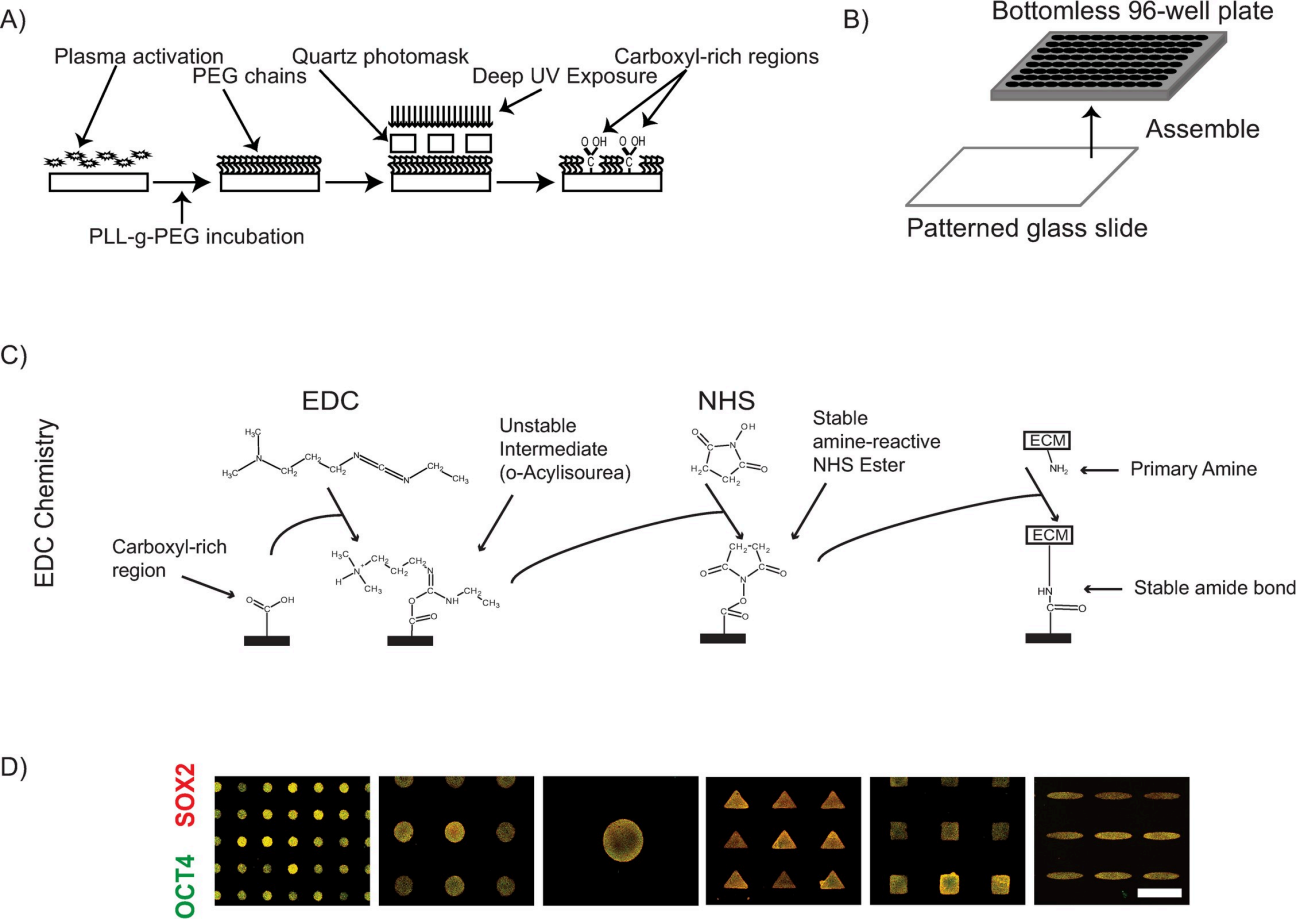
Development of PEG based micropatterning platform. (A) Scheme of protocol for transferring carboxyl-rich micro-patterns onto glass coverslips. (B) Overview of assembly procedure to produce 96-well microtiter plates with micropatterned culture surface. (C) Overview of carbodiimide- and succinimide-based ECM protein immobilization scheme. The carboxyl groups induced on the patterned glass coverslips react with EDC to form an unstable intermediate molecule O-Acylisourea. If kept in an aqueous environment for an extended period of time, this activated state may hydrolyze and result in the loss of activation of the carboxyl group. O-Acylisourea reacts with the present NHS molecules to form a stable amine-reactive NHS ester. This activated state is stable when kept dry, and the plates can be stored dry for some time after this activation step. Upon incubation with proteins (for instance, ECM proteins), it results in a covalent immobilization of the protein to the substrate. (D) Representative immunofluorescent images of micropatterned hPSCs colonies generated using the protocol depicted in panels A through C stained for OCT4 and SOX2. ECM, extracellular matrix; EDC, 1-Ethyl-3-(3-dimethylaminopropyl)carbodiimide; NHS, N-HydroxySuccinimide; OCT4, octamer-binding transcription factor 4 (also known as POU5F1); PEG, Polyethylene Glycol; PLL-g-PEG, Poly-L-Lysine-grafted-Polyethylene Glycol.

Photo-oxidation of organic polymers like PEG—a widely reported bio-inert polymer [42], by Deep UV (DUV) light has been shown to up-regulate carboxyl groups [40,41], which can be readily biofunctionalized with ECM proteins [43]. We employed this knowledge to develop a protocol to generate micropatterned, carboxyl-rich regions (Fig 1A) [24]. We confirmed that incubation with Poly-L-Lysine-grafted-Polyethylene Glycol (PLL-g-PEG) resulted in a PEGylated surface on plasma treated borosilicate glass coverslips by probing the carbon 1s (C1s) spectra profile using X-Ray Photoelectron Spectroscopy (XPS). Consistent with previous reports [40], a peak indicating the presence of the C-O-C functional group present in PEG was detected at 286.6 eV in the C1s spectrum on the PLL-g-PEG incubated glass coverslip, in addition to the peak at 285 eV that was observed in the blank glass coverslip control (S1A Fig). DUV treatment of the PEGylated coverslips progressively reduced the peak at 286.6 eV (S1B Fig), suggesting photo-oxidation mediated ablation of the PEG layer. However, we were unable to detect any carboxyl presence, which has been reported to occur at 289 eV [40]. We hypothesized that the photo-oxidation of the PEG during DUV treatment reduced the polymer thickness below the detection limit of the XPS equipment employed in this study, causing the absence of the carboxyl peak in the emission spectra. Given that biochemical assays circumvent the need of minimum polymer thickness to detect the presence of functional groups of interest, we opted to employ a previously reported assay based on the preferential affinity of Toluidine blue-O (TBO) to carboxyl functional groups (see Materials and methods for assay description) [44] and asked whether DUV treatment changed the amount of TBO adsorbed onto PEGylated coverslips. Indeed, DUV treatment resulted in an increase in the amount of TBO adsorption on PEGylated coverslips, with relative levels increasing with exposure times up to 12 min after which the relative levels detected decreased (S1C Fig). These findings indicate that, consistent with previous reports [40,45], PLL-g-PEG incubation results in PEGylation of coverslips, and that the optimal exposure time to maximize the presence of carboxyl functional groups on the PEGylated coverslips in our experimental setup was 12 min. To produce 96-well microtiter plates for patterned cell-culture surfaces, PEGylated large coverslips (110 mm × 74 mm) were photopatterned by DUV exposure through Quartz photomasks for 12 min and assembled to bottomless 96-well plates (Fig 1B). Carboxyl groups were activated using carbodiimide and succinimide chemistry [43] (Fig 1C) to enable covalent attachment to primary amines on ECM molecules. This PEG plates platform enabled robust geometrical-confinement of a variety of cell types in colonies of a variety of shapes and sizes (Fig 1D and S1D–S1I Fig).

Given the vital role that interactions between cells and the surrounding ECM play on cellular responses [46], we next asked whether the approach of covalent attachment of ECM molecules interfered with fate decisions of hPSCs micropatterned on the PEG plates. We opted to employ a recently reported 2-day assay using OCT4 and SOX2 expression as readouts to assess fate decisions in geometrically confined hPSC colonies [30] (S2A Fig) and directly compared fate acquisition of hPSCs on the PEG plates with μCP plates, a micropatterning technique that does not require any chemical immobilization of ECM molecules. We observed a highly correlated (R^2^ > 0.9) differentiation response between μCP and PEG plates (S2B Fig and S2C Fig). Furthermore, the PEG plates responded in a more reproducible manner than the μCP plates both in terms of the number of colonies achieved per well of a 96-well plate and the number of cells attached per colony (S2D Fig and S2E Fig). Taken together, these data demonstrate that the PEG plates enable robust geometric confinement of cell colonies, and the differentiation response of hPSC colonies micropatterned using the PEG plates differentiate in a highly correlated manner to those micropatterned on μCP plates, making them a valuable platform for high-throughput screening studies for the bioengineering community.

### hPSC line screen for peri-gastrulation–like patterning response yields variable responses

**Fig 2:**
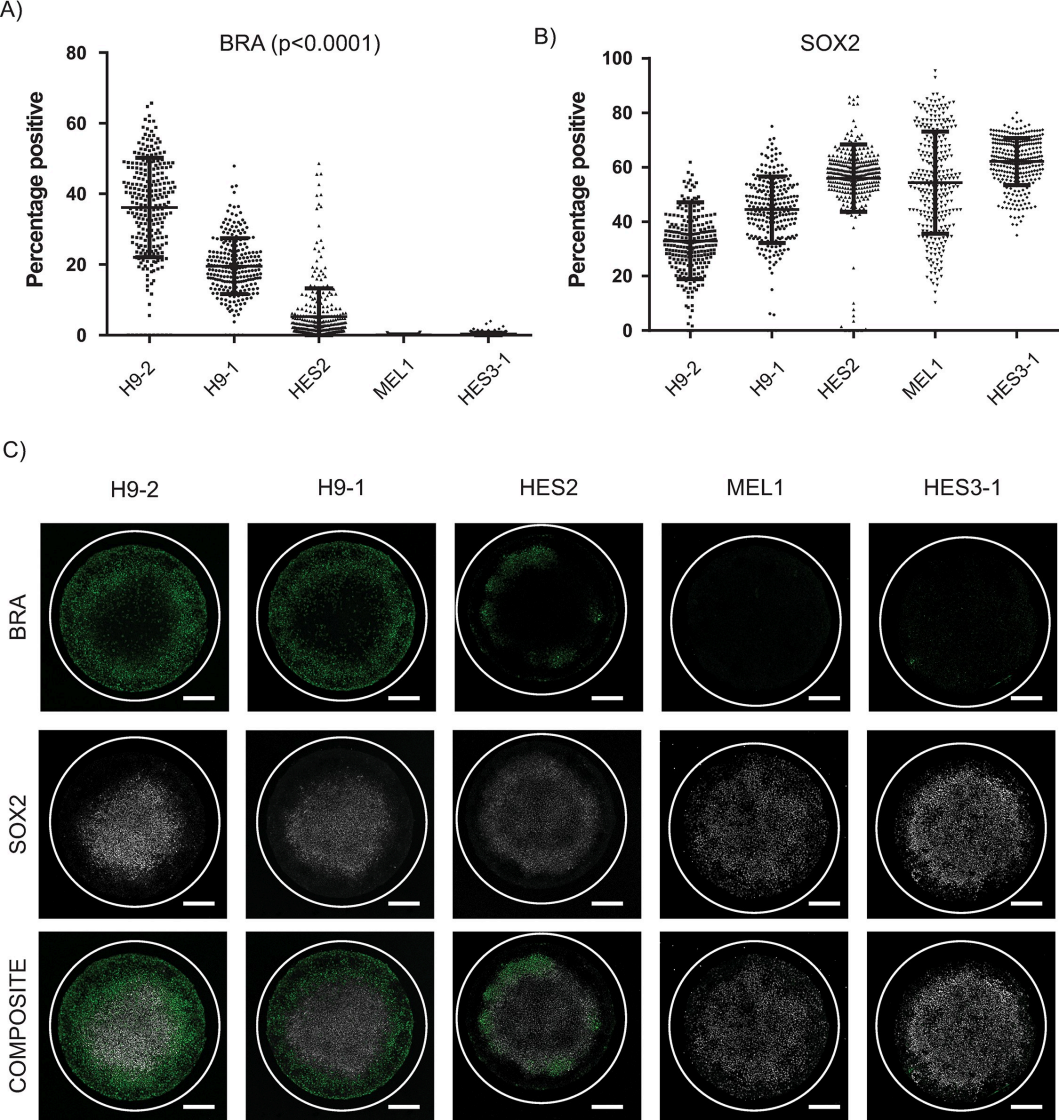
Variability in peri-gastrulation–like induction observed between test hPSC lines. (A–B) Quantified expression of BRA (A) and SOX2 (B) observed within the assayed hPSC lines tested. Number of colonies were 252, 245, 327, 288, and 304 for H9-2, H9-1, HES2, MEL1, and HES3-1, respectively. Each data point represents individual colonies identified. Data pooled from 2 experiments. (C) Representative immunofluorescent images for BRA and SOX2 for the test hPSC lines. Scale bar represents 200 μm. For DAPI staining, please see S3B Fig. Underlying numerical data for this figure can be found in https://osf.io/zrvxj/. BRA, Brachyury; hPSC, human pluripotent stem cell; SOX2, SRY (sex determining region Y)-box 2.

Recent studies have reported that BMP4 treatment of geometrically confined hPSC colonies results in organized fate patterning of gastrulation-associated markers [24–26]. Notably, these studies demonstrated that the differentiating geometrically confined hPSC colonies gave rise to a Brachyury (BRA)-expressing compartment, representing a primitive-streak–like identity. Given that lineage-specific differentiation potential between hPSC lines is known to vary widely [28–30], we hypothesized that different hPSC lines would induce the primitive-streak–like compartment at different efficiencies. We employed our platform to evaluate the response of BMP4-treatment of geometrically confined hPSC colonies (1 mm in diameter) in a screen of the following 5 hPSC lines: H9-1, H9-2, HES2, MEL1, and HES3-1. The induction medium employed for this screen, and all subsequent experiments (unless otherwise stated) was a knockout serum replacement–based medium supplemented with BMP4 and bFGF (see Materials and methods for composition). Although all hPSC lines tested expressed high levels of pluripotency markers at the start of the differentiation culture (S3 Fig), induction of BRA expression levels varied markedly between hPSC lines at 48 h after BMP4 treatment (Fig 2A and 2C). Notably, although the MEL1 and HES3-1 lines were unable to induce the expression of BRA, they did differentiate as indicated by the reduction of SOX2 expression relative to the starting population (Fig 2B and 2C and S3 Fig). These data indicate that although all hPSC lines tested under these experimental conditions differentiated upon BMP4 treatment, induction of the primitive-streak–like compartment, as indicated by BRA expression, varied considerably.

### hPSC differentiation propensities are correlated with endogenous Nodal signalling

**Fig 3:**
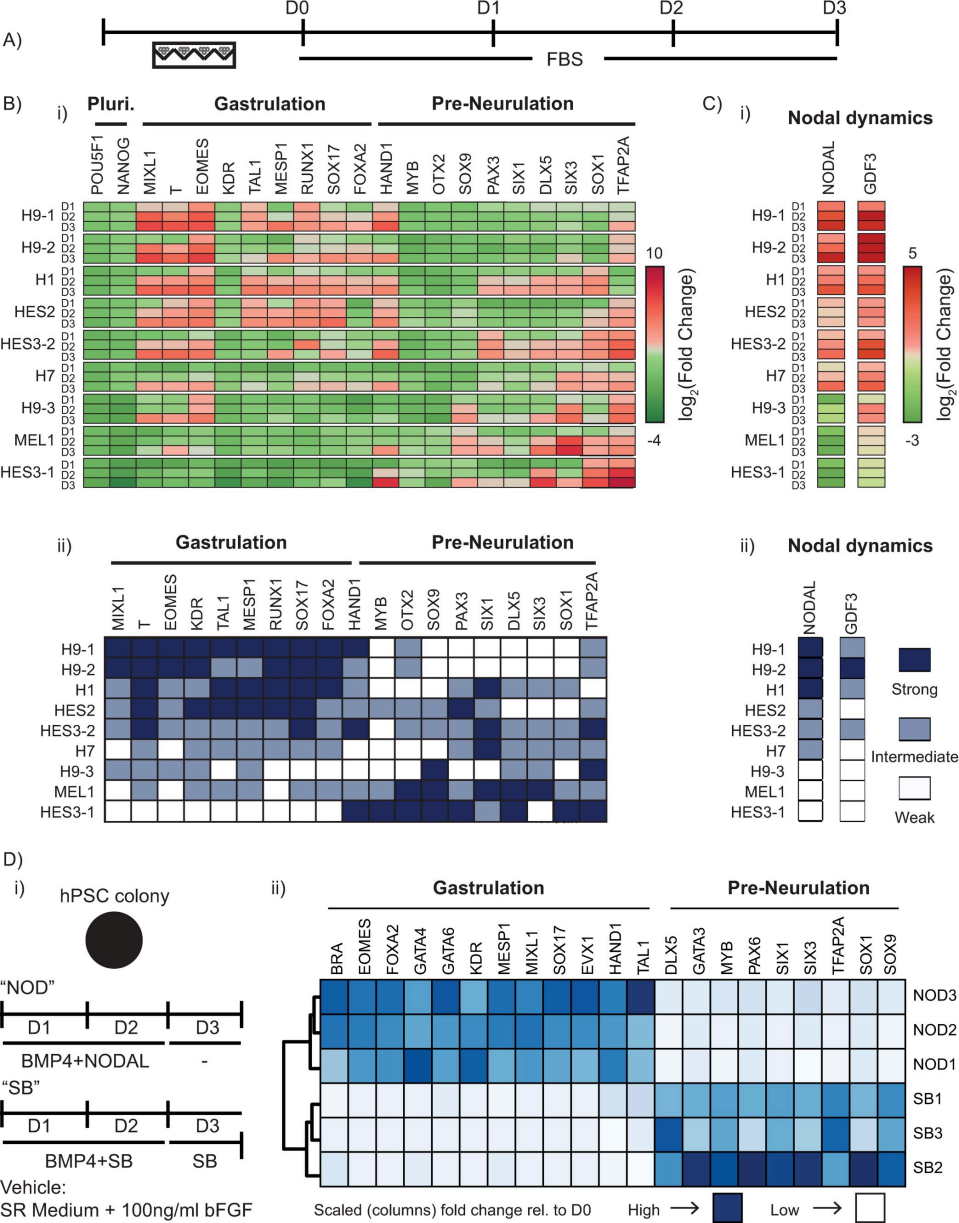
Nodal bisects gastrulation- and preneurulation-associated gene expression profiles. (A) Overview of experimental setup for EB assay. EBs were made from each test hPSC line and allowed to spontaneously differentiate in the presence of FBS for 3 days. (B) Observed gene expression dynamics of test cell lines when differentiated as EBs in FBS. (i) Observed gene expression for a panel of differentiation-associated genes (shown under ‘Gastrulation’ and ‘Pre-Neurulation’ groups) along with POU5F1 (OCT4) and NANOG. Data shown as heat map of mean expression of each day from 3 biological replicates, represented as log_2_(Fold Change) relative to the D0 sample of respective hPSC line. ‘Pluri’ indicates the pluripotency associated genes. (ii) Heat map representation of panel B(i) with the panel of hPSC lines clustered into 3 groups of ‘Strong’, ‘Intermediate’, and ‘Weak’ responders for each gene using unsupervised K-means clustering. ‘Pluri’ indicates the pluripotency associated genes. (C) Nodal dynamics during EB assay. (i) Observed gene expression of Nodal and a Nodal signalling target (GDF3). Data shown as heat map of mean expression of each day from 3 biological replicates (expression levels for individual replicates shown in S4 Fig), represented as log_2_(Fold Change) relative to the D0 sample of the respective hPSC line. (ii) Heat map representation of panel C(i) with the panel of hPSC lines clustered into 3 groups of ‘Strong’, ‘Intermediate’, and ‘Weak’ responders for Nodal and GDF3 using unsupervised K-Means clustering. (D) Effect of modulation of Nodal in the peri-gastrulation–like assay using geometrically confined colonies of the ‘CA1’ hPSC line. (i) Overview of the experimental setup. Geometrically confined colonies of CA1s were induced to differentiate for 3 days, with either a 2-day pulse of BMP4 and Nodal and just Nodal for the third day or a 2-day pulse of BMP4 and an inhibitor of Nodal signalling (SB431542, ‘SB’) and just SB for the third day. The vehicle employed in this experiment was SR medium (see Materials and methods for composition). (ii) Heat map representation of a panel of differentiation genes associated with either gastrulation or preneurulation. Dark blue represents higher levels of expression, whereas light blue represents lower levels of expression. Data shown as mean of 3 biological replicates. Expression levels of individual replicates shown in S7 Fig. Underlying numerical data for this figure can be found in https://osf.io/zrvxj/. BMP4, bone morphogenetic protein 4; BRA, brachyury (also known as T); DLX5, distal-less homeobox 5; EB, embryoid body; EOMES, Eomesodermin; FOXA2, forkhead box protein A2; GDF3, growth differentiation factor 3; HAND1, Heart- and neural crest derivatives-expressed protein 1; hPSC, human pluripotent stem cell; KDR, Kinase insert domain receptor; MESP1, mesoderm posterior bHLH transcription factor 1; MIXL1, Mix paired-like homeobox protein 1; MYB, Myb proto-oncogene protein; NANOG, Homeobox protein NANOG; OTX2, Orthodenticle homeobox 2; PAX3, paired box gene 3; POU5F1, POU domain, class 5, transcription factor 1 (also known as OCT4); RUNX1, Runt-related transcription factor 1; SIX1, Sineoculis homeobox homolog 1; SIX3, Sineoculis homeobox homolog 3; SOX1, SRY-box transcription factor 1; SOX17, SRY-box transcription factor 17; SOX9, SRY-box transcription factor 9; SR, serum replacement; T, T box transcription factor T (also known as Brachyury); TAL1, T-cell acute lymphocytic leukemia protein 1; TFAP2A, transcription factor AP2-alpha.

We hypothesized that differences in regulation of key signalling pathways controlling mesendodermal induction between the tested hPSC lines underlay the variation in BRA expression observed in the peri-gastrulation–like patterning. To test this hypothesis, we employed a recently reported approach that addressed a similar question in mouse epiblast stem cell (mEpiSC) lines [47]. In their study, Kojima and colleagues made embryoid bodies (EBs) out of various mEpiSC lines, allowed them to spontaneously differentiate in culture conditions unsupportive of pluripotency, and assayed for the expression of differentiation-associated genes to compare the transcriptional and functional profiles between the lines [47]. Employing a similar approach, we generated EBs from 9 hPSC lines—H9-3, H1, H7, HES3-2 in addition to the previous panel (complete list of lines and their respective culture conditions shown in S1 Table)—and cultured them in conditions unsupportive of pluripotency for 3 days and analyzed differentiation marker gene expression levels daily (henceforth ‘EB assay’; Fig 3A). We observed strong variation in expression profiles of differentiation-associated genes between the test hPSC lines (Fig 3Bi). To simplify data interpretation, we used unsupervised K-means clustering to segregate the hPSC lines into ‘Strong’, ‘Intermediate’, and ‘Weak’ expressers for each gene tested. This analysis revealed distinct sets of responses in the test lines in which some lines up-regulated expression of genes associated with gastrulation whereas others up-regulated expression of preneurulation-associated genes (Fig 3Bii). In a recent study, Funa and colleagues showed that Wnt signalling mediated differentiation of hPSCs results in fate acquisition that is dependent on Nodal signalling [48]. Specifically, the authors demonstrated that the presence of Nodal signalling during Wnt-mediated differentiation of hPSCs resulted in the acquisition of a primitive streak fate, whereas the absence of Nodal signalling during Wnt-mediated differentiation resulted in the induction of the neural crest fate [48]. Given that the primitive streak is a gastrulation-associated fate, neural crest arises during neurulation, and the fact that our data demonstrated a gastrulation versus preneurulation switch in differentiating hPSC lines, we hypothesized that difference in Nodal signalling could be responsible for these gene expression profiles in our EB assay. Consistent with this hypothesis, the expression of Nodal and GDF3 (a Nodal target) in the differentiating hPSC lines showed a strong trend indicative of their up-regulation linked with the induction of gastrulation-associated genes and their absence linked with the induction of preneurulation-associated genes (Fig 3Ci). Furthermore, clustering the hPSC lines with reference to the expression profiles of Nodal and GDF3 by unsupervised K-means clustering (Fig 3Cii and S4 Fig) indicated that up-regulation of Nodal and GDF3 coincided with gastrulation-associated gene expression, whereas their down-regulation corresponded with preneurulation-associated gene expression.

### Validation of gene expression responses in EB assay

We next sought to validate the gene expression differences observed in the differentiating hPSC lines in the EB assay by asking if the variation would influence cell fate acquisition during directed differentiation. Given the key role that MIXL1 plays in the induction of definitive endoderm [49], Kojima and colleagues investigated the expression profiles of Mixl1 in their EB assay with mEpiSCs and demonstrated that Mixl1 expression was predictive of endodermal differentiation bias of the mEpiSC lines [47]. Importantly, much like MIXL1, EOMES is also known to play an important role in the endoderm specification [50,51]. Consistent with this idea, the ‘Strong’, ‘Intermediate’, and ‘Weak’ responders of the panel of hPSCs for both MIXL1 and EOMES contained the identical hPSC line cohorts (Fig 3Bii and S5A Fig), suggesting the likelihood of parallel functions of both these genes in differentiating hPSCs. To validate the gene expression profiles observed in our EB assay, we asked if the observed expression differences of these genes that critically regulate endoderm specification were able to predict the propensity of the hPSC lines to differentiate toward the definitive endodermal fates. Consequently, we differentiated the panel of hPSCs toward definitive endoderm using an established protocol (S5B Fig), and consistent with the findings of Kojima and colleagues [47], the expression profiles of endoderm specifiers (MIXL1 and EOMES in our case) in our EB assay closely matched the propensity of the hPSC lines to induce SOX17 expression upon directed differentiation toward the definitive endoderm fate (S5C Fig and S5D Fig). The differential expression profiles of MIXL1 and EOMES in the EB assay were also able to predict the induction efficiency of mature endodermal fates. Specifically, lines from the MIXL1/EOMES-Strong cluster outperformed candidate lines from the MIXL1/EOMES-Weak cluster in the induction of pancreatic progenitors as marked by the co-expression of PDX1 and NKX6.1 (S5E Fig). These data provide protein-level phenotypic validation of the variable gene expression observed in our EB assay.

Given that geometrically confined hPSC colonies are able to induce organized fate patterning [24–26], we asked if subjecting geometrically confined hPSC colonies to defined endodermal differentiation conditions could be used as an assay to predict the differentiation propensity of hPSC lines. We selected 3 hPSC lines—H9-1, HES3-2, and HES3-1—to represent each MIXL1/EOMES induction compartment defined in the EB assay (Fig 3Bii and S5A Fig) and differentiated them as geometrically confined colonies in defined endodermal induction conditions (S6A Fig). Interestingly, we found that the relative efficiency of SOX17 and FOXA2 double-positive expression under these experimental conditions closely matched the endodermal lineage bias of the lines as predicted by the EB assay (S5A–S5D Fig, S6B Fig and S6C Fig). Taken together, these data validate the differential gene expression observed between the panel of hPSC lines by demonstrating congruence between MIXL1 and EOMES temporal dynamics and endoderm lineage bias of hPSC lines and provide proof-of-concept data that the defined differentiation protocols in geometrically confined hPSC colonies can be used as quick assays to assess lineage bias of hPSC lines.

### Nodal bisects gastrulation- versus preneurulation-associated hPSC differentiation

Thus far, our data showed that in conditions that do not support pluripotency, differentiating EBs made from hPSC lines assume a transcriptional state associated with either gastrulation or preneurulation, and endogenous Nodal dynamics correlated with this switch. However, whether the differential Nodal dynamics caused the switch in the acquired transcriptional state or if the association was correlative remained unclear. Given that BMP4 treatment has been previously reported to induce gastrulation-associated fate patterning [24–26] and that we have previously demonstrated the robust response of peri-gastrulation-like fate acquisition in the CA1 hPSC line [24], we revisited the peri-gastrulation–like model in hPSC colonies to test whether Nodal signalling had a direct effect in regulating this switch. We asked if inducing geometrically confined colonies of the CA1 line to differentiate in response to BMP4 either in the presence or absence of a small molecule inhibitor of Alk4/5/7 receptors (SB431542, hereafter ‘SB’) which antagonizes Nodal signalling (Fig 3Di) recapitulated the observed switch in emergent gene expression. After a 3-day induction, we observed that colonies grown in the presence of SB up-regulated genes associated with preneurulation, whereas those grown in the absence of SB up-regulated genes associated with gastrulation (Fig 3Dii, S7 Fig). These results are consistent with our hypothesis that Nodal signalling distinguishes gastrulation- and preneurulation-associated gene expression profiles in differentiating hPSCs. Given that differentiating hPSCs in the absence of Nodal signalling up-regulated preneurulation-associated genes, we next set to investigate if BMP4 treatment of geometrically confined hPSC colonies in the presence of SB gave rise to early neurulation-associated spatially patterned fate allocation.

### An RD network in BMP signalling can organize pSMAD1 activity independent of Nodal

**Fig 4:**
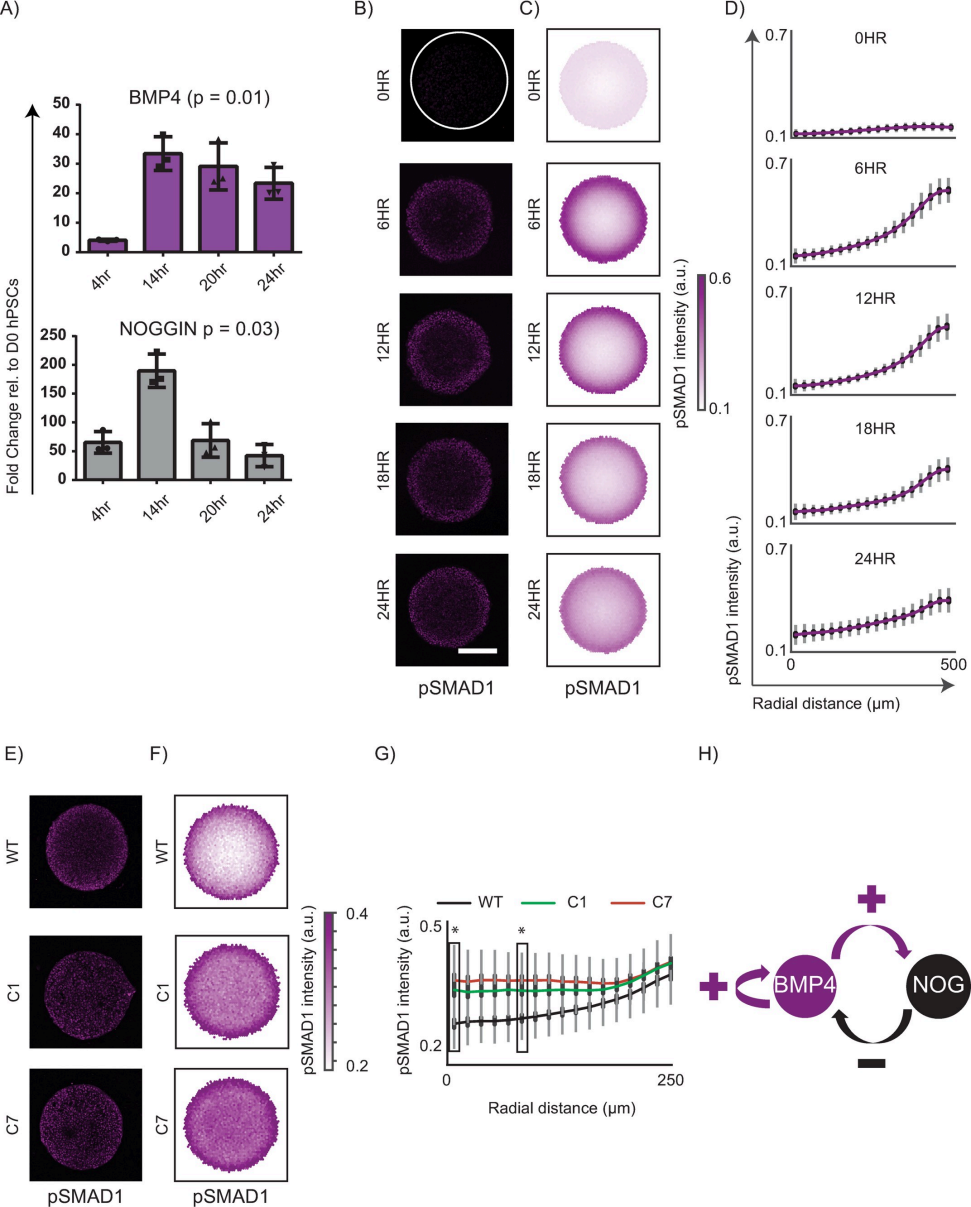
In the absence of Nodal, an interaction network between BMP4-Noggin underlies organization of pSMAD1 gradient. (A) Temporal gene expression for BMP4 and Noggin at 4 h, 14 h, 20 h, and 24 h after BMP4 and SB treatment. Data shown as mean ± SD of 3 independent experiments. The *p*-values shown were calculated using Kruskal-Wallis test. Underlying numerical data for this figure can be found in the file Fig 4A in OSF repository in the project named after the paper title. (B) Representative immunofluorescent images of geometrically confined hPSC colonies of 500 μm in diameter stained for pSMAD1 after different times (0 h, 6 h, 12 h, 18 h, and 24 h) of BMP4 and SB exposure. Scale bar represents 200 μm. (C) Average pSMAD1 intensity observed in colonies treated with BMP4 and SB represented as overlays of 231, 241, 222, 238, and 228 colonies for respective induction times. Data pooled from 2 experiments. (D) The average radial trends of pSMAD1 at each duration shown as line plots. SDs shown in grey, and 95% confidence intervals shown in black. Underlying numerical data for this figure can be found in the file Fig 4D in OSF repository in the project named after the paper title. (E–G) Response of pSMAD1 organization in homozygous knockout lines of Noggin. (E) Representative immunofluorescent images of geometrically confined hPSC colonies of WT, Noggin^−/−^ clones C1, and C7 (characterization shown in S8 Fig) stained for pSMAD1 after 24 h of BMP4 exposure in the presence of SB. (F–G) The average radial trends of pSMAD1 for the WT, C1, and C7 clones shown as overlays of all identified colonies (F) and line plots (G). Data pooled from 2 experiments and include 151, 150, 150 colonies for each line, respectively. SDs shown in grey, and 95% confidence intervals shown in black. The *p*-values were calculated using Mann-Whitney U test. *p < 0.0001 for each clone relative to the WT control. Underlying numerical data for this figure can be found in the file Fig 4G in OSF repository in the project named after the paper title. (H) Model of RD mediated organization of pSMAD1. Underlying numerical data for this figure can be found in https://osf.io/zrvxj/. BMP4, bone morphogenetic protein 4; hPSC, human pluripotent stem cell; OSF, open science framework; pSMAD1, phosphorylated SMAD1; RD, reaction-diffusion; SB, SB431541 (Nodal signalling antagonist); WT, wild type.

In a recent study, we demonstrated that the peri-gastrulation–like fate patterning in geometrically confined hPSC colonies occurs via a coordinated process of RD and PI in which a BMP4-Noggin RD network organizes a phosphorylated SMAD1 (pSMAD1) signalling gradient within the colonies, resulting in the peri-gastrulation–like fates being patterned in a manner consistent with the PI paradigm [24]. We set out to investigate whether a conserved mechanism would give rise to preneurulation-associated fate patterning. As a first step, we asked if a BMP4-Noggin RD network governed pSMAD1 organization within the geometrically confined hPSC colonies treated with BMP4 and SB. Consistent with the presence of a BMP4-Noggin RD network in experimental conditions that included Nodal [24], we observed an up-regulation of both BMP4 and Noggin upon BMP4 treatment of hPSCs in the presence of SB (Fig 4A). We next asked whether BMP4 treatment of geometrically confined hPSC colonies in the presence of SB would result in the organized gradient of nuclear-localized pSMAD1. Indeed, pSMAD1 activity within the colonies rapidly organized into a radial gradient under these experimental conditions (Fig 4B–4D). We have previously showed that in the presence of Nodal signalling, Noggin can play a role in the formation of the pSMAD1 gradient by demonstrating that Noggin inhibition by small interfering RNA (siRNA) resulted in a significant increase in pSMAD1 activity in the colony centre [24]. To ascertain whether Noggin retains its function in the organization of the pSMAD1 gradient in the absence of Nodal signalling (SB supplementation), we generated 2 homozygous knock-outs of Noggin (‘C1’, and ‘C7’) using CRISPR/CAS9 (characterization of lines shown in S8 Fig) and asked whether the absence of Noggin compromised the organization of pSMAD1 in induction medium supplemented with BMP4 and SB. Consistent with our hypothesis that a BMP4-Noggin RD network was underlying the pSMAD1 organization, the formation of the pSMAD1 signalling gradient under these experimental conditions was significantly compromised in Noggin knockout lines C1 and C7 compared with the wild-type control (Fig 4E–4G), indicating an integral involvement of Noggin in the organization of the pSMAD1 signalling gradient. In our previous study using the peri-gastrulation–like model, we showed that a BMP4-Noggin RD computational model predicts the experimentally observed responses of a pSMAD1 organized gradient at the periphery and the centre of the colonies to perturbations to the BMP4 dose in the induction medium and size of the geometrically confined hPSC colony [24]. Specifically, we showed that reducing the BMP4 dose while maintaining the colony size reduces the levels of pSMAD1 at the periphery, and reducing the colony size while maintaining a constant BMP4 dose in the induction medium results in an increase of pSMAD1 levels at the centre of the colonies [24]. We reasoned that a conserved mechanism underlying the pSMAD1 organization would result in identical responses to these perturbations. Consistent with our anticipated results, reducing the BMP4 dose in the induction medium while maintaining the colony size resulted in a reduction of the detected immunofluorescent levels of nuclear-localized pSMAD1 at the colony periphery (S9A–S9C Fig). Furthermore, reducing the colony size while maintaining the BMP4 dose in the induction medium increased the detected immunofluorescent levels of nuclear localization of pSMAD1 at the colony centres (S9D Fig and S9E Fig). Taken together, these data demonstrate that in the absence of Nodal signalling, pSMAD1 activity in the geometrically confined hPSC colonies organizes into a signalling gradient and suggest that a BMP4-Noggin RD system governs this observation (Fig 4H)—consistent with our previous study [24].

### Nodal signalling contributes to the shape of the organized pSMAD1 gradient

**Fig 5:**
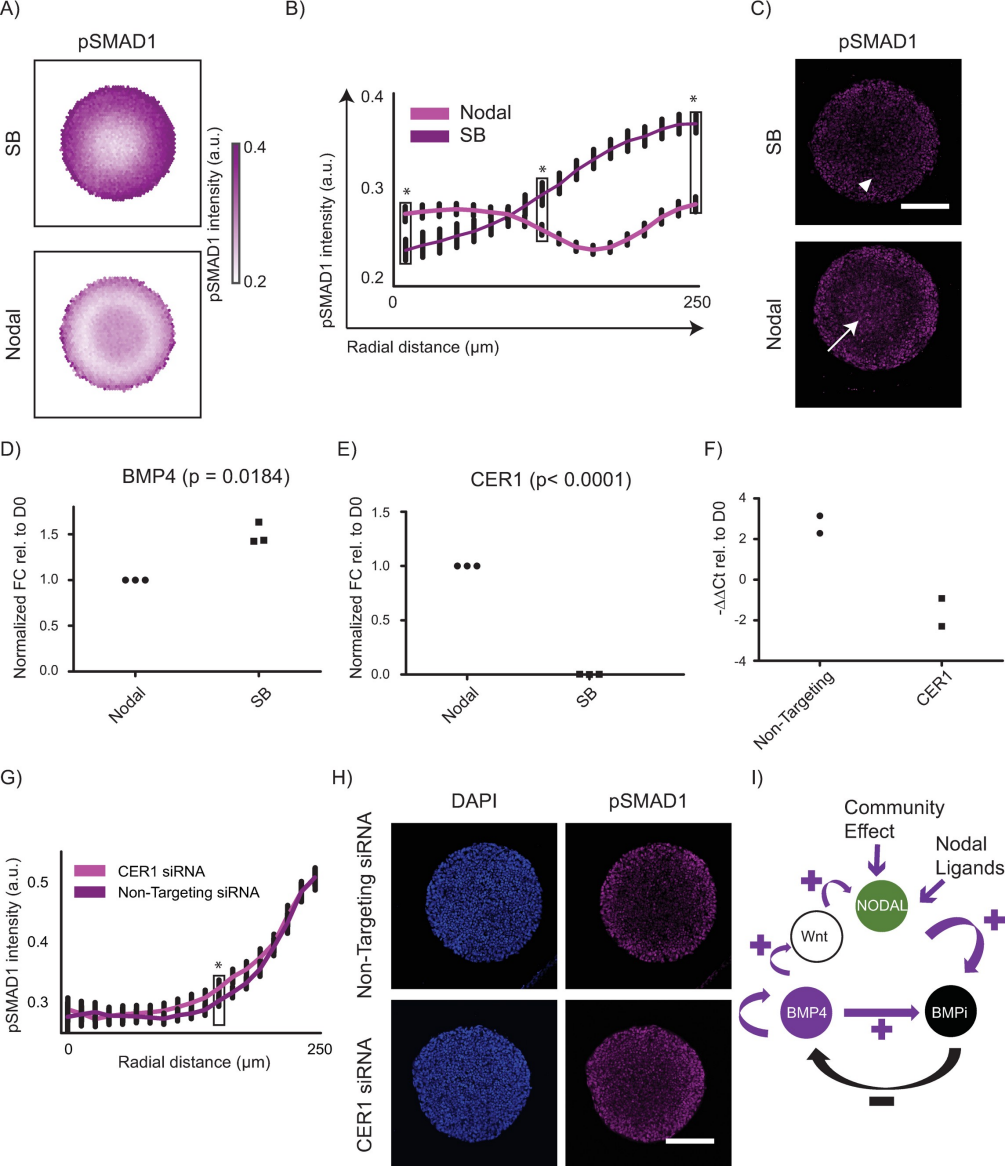
Nodal signalling contributes to the formation of the pSMAD1 gradient. (A–C) Perturbing Nodal signalling results in a significant change in the pSMAD1 organized gradient formation. (A) Average pSMAD1 intensity detected with colonies were treated with 25 ng/ml of BMP4 for 24 h either in the presence of Nodal or SB represented as overlays of 188 and 163 colonies for Nodal and SB conditions, respectively. Data pooled from 2 experiments. (B) The average radial trends of pSMAD1 for SB and Nodal conditions when treated with 25 ng/ml of BMP4 shown as line plots. SDs shown in black. The *p*-values were calculated using Mann-Whitney U test. *p < 0.0001. Underlying numerical data for this figure can be found in the file Fig 5B in OSF repository in the project named after the paper title. (C) Representative immunofluorescence images of 500 μm diameter hPSC colonies stained for pSMAD1 after 24 h of BMP4 treatment (25 ng/ml) in ‘Nodal’ and ‘SB’ conditions (average response shown in panel A). Scalebar represents 200 μm. White arrows indicate regions where second peak of pSMAD1 appears. White triangles indicate regions of discernable pSMAD1 levels that appear to be lower than the levels at the colony periphery. (D–E) Changes in the activator and antagonists of BMP signalling upon perturbation of Nodal signalling. (D) BMP4 expression after 24 h of treatment of hPSCs with BMP4 containing media supplemented with either Nodal or SB. (E) CER1 expression after 24 h of treatment of hPSCs with BMP4 containing media supplemented with either Nodal or SB. Data in panels D and E represent 3 biological replicates and are shown as normalized (with respect to the expression observed in Nodal supplemented media) fold change relative to the Day 0 hPSC population for each biological replicate. The *p*-values were calculated using two-sided paired t-test. (F–H) siRNA mediated inhibition of CER1 increases the level of pSMAD1 detected in an interior band of the hPSC colonies when treated with BMP4 and Nodal. The experiments described in panels F through H were repeated twice. (F) CER1 expression controls for the 2 experiments in panels G and H showing a change in the levels of CER1 transcripts detected in the presence of a CER1 siRNA or a nontargeting siRNA. (G) Average radial trends of pSMAD1 for CER1 siRNA (45 colonies) and nontargeting siRNA (37 colonies) conditions shown as line plots. SDs shown in black. The *p*-values were calculated using Mann-Whitney U test. *p < 0.0001. (H) Representative immunofluorescent images of observed pSMAD1 staining for the condition when the colonies were treated with a CER1 siRNA versus a nontargeting siRNA. (I) Model of involvement of Nodal in the contribution to the pool of BMP signalling antagonists that govern the RD response observed in the BMP signalling pathway. BMP4 activates itself and its inhibitors like Noggin. In addition to Nodal signalling activating in the experiments because of the Nodal ligands present in the induction medium, BMP4 can also activate Nodal through Wnt, or Nodal can be induced because of a community effect in dense cultures. Nodal in turn can activate downstream targets like CER1 that are BMP antagonists, which contribute to a pool of BMP antagonists ‘BMPi’ that together antagonize BMP signalling. Underlying numerical data for this figure can be found in https://osf.io/zrvxj/. BMP4, bone morphogenetic protein 4; CER1, Cerberus; hPSC, human pluripotent stem cell; OSF, open science framework; pSMAD1, phosphorylated SMAD1; RD, reaction-diffusion; SB, SB431542 (Nodal signalling antagonist); siRNA, small interfering RNA.

Our data indicate that the pSMAD1 signalling gradient organizes via an RD network present in the BMP signalling pathway in which Noggin functions as an important inhibitor (Fig 4). Given that Nodal signalling targets include multiple BMP antagonists such as CER1, GDF3, Follistatin (FST), etc. [52], we asked whether Nodal signalling contributed to the formation of the pSMAD1 signalling gradient in BMP4-treated geometrically confined hPSC colonies. To probe the role of Nodal in the observed pSMAD1 organization, we compared the formation of the pSMAD1 gradient in geometrically confined hPSC colonies of 500 μm diameter treated with BMP4 for 24 h in which the induction media either contained Nodal ligands or SB. The pSMAD1 signalling gradients formed in the presence and absence of Nodal signalling were significantly different from each other when treated with either 25 ng/ml or 50 ng/ml of BMP4 in the induction medium demonstrating the involvement of Nodal signalling in the formation of the organized signalling gradient (Fig 5A–5C and S10A–S10E Fig). The results from these studies provided a few notable observations. First, in 500 μm diameter colonies treated for 24 h with 25 ng/ml BMP4 in presence of Nodal ligands, we observed 2 prominent peaks of pSMAD1 expression—one peak was observed at the periphery as expected from previous reports [24–26] and another one at the colony centre that has not been previously reported in colonies of this size (Fig 5A–5C and S10A Fig). This observation provides further support to the proposition that the organization of pSMAD1 is consistent with an RD mechanism that can result in spatial oscillations of morphogen activity [24], while providing evidence of crosstalk between BMP and Nodal signalling pathways in establishing the pSMAD1 signalling gradient. Another notable observation was that, in the instance when the colonies were treated with 50 ng/ml of BMP4, the pSMAD1 expression that declined from the periphery of the colonies dropped rapidly in the presence of Nodal ligands, but the decline was far more gradual in the presence of SB (S10B–S10E Fig). Cells expressing discernible levels of pSMAD1 were observed much farther into the colony from the periphery than observed in the condition when Nodal ligands were present in the induction medium. Finally, in the instance when the colonies were treated with 25 ng/ml of BMP4, the level of pSMAD1-associated immunofluorescence detected at the colony periphery in the presence of SB was significantly higher than the levels detected in the presence of Nodal (Fig 5A–5C). Taken together, these data provides further justification for the hypothesis that the pSMAD1 signalling gradient arises via an RD mechanism and demonstrate that Nodal signalling contributes to the shape of the organized pSMAD1 signalling gradient.

Because an RD network results in the morphogen gradient as a consequence of the expression of both activators and inhibitors of the morphogen [11,12], we hypothesized that this observation could be due to either a change in the amount of activator (change in BMP4 levels) or a change in the amount of inhibitor (change in the level of BMP antagonists) in the system. When we tested gene expression of activators and inhibitors after 24 h of Nodal versus SB treatment on hPSCs which either allowed expression of Nodal and Lefty-A (a Nodal target) or dramatically down-regulated their expression (S10F and S10G Fig), SB treatment provoked an increased positive feedback as indicated by increased detected levels of BMP4 transcripts (Fig 5D) and a reduced negative feedback as indicated by significantly reduced transcript levels of BMP antagonists like CER1 (Fig 5E), GDF3 (S10H Fig), and FST (S10I Fig). To validate our hypothesis that CER1 could function as an inhibitor that participates in the RD-mediated organization of pSMAD1 signalling, we asked whether inhibiting CER1 using siRNA treatment would have an effect in the organization of the pSMAD1 gradient. Consistent with our hypothesis, we found that a modest reduction in the expression of CER1 upon siRNA treatment (Fig 5F) resulted in a significant change in the pSMAD1 profile that organized in the hPSC colonies upon BMP4 treatment (Fig 5G and 5H). Taken together, these data suggest that Nodal signalling can contribute to the RD-mediated organization of the pSMAD1 signalling gradient and that this contribution might occur because of a change in the levels of activators and antagonists of BMP signalling. Notably, the activation of Nodal signalling may occur via various modes including because of the Nodal ligands present in the media (see Materials and methods), through the BMP-Wnt-Nodal axis that arises during gastrulation, or because of community-effect mediated activation in these dense hPSC cultures (Fig 5I). Having established that pSMAD1 activity in the geometrically confined hPSC colonies treated with BMP4 and SB organizes into a signalling gradient, we next focused on investigating whether this gradient induced the expression of fates associated with the differentiating ectoderm.

### Nodal switches BMP signalling–mediated peri-gastrulation–like and preneurulation-like fate patterning

Given that we observed differentiating hPSCs in the absence of Nodal signalling up-regulate a preneurulation-associated gene profile (Fig 3D), we asked whether BMP4 and SB treatment of geometrically confined hPSC colonies resulted in the fate patterning associated with the early stages of the differentiating ectoderm. Consensus studies in vertebrate embryos have identified that after the germ layers segregate from the epiblast, a BMP signalling gradient along the medial-lateral axis in the developing ectoderm patterns the early preneural (PN) tissue at the medial end, and non-neural (NN) tissue at the lateral end appropriately arranging the tissue for the onset of neurulation [8]. The PN tissue gives rise to the neural plate (NP), which later folds to form the neural tube [5,7] and the NN tissue gives rise to the NN ectoderm (NNE) and the neural plate border (NPB) [8]. The NNE subsequently specifies to generate the epidermis, and the NPB is a multipotent tissue that produces the neural crest (NC) and the craniofacial placodes in the anterior ectoderm [8,9,53–55]. Whereas the early PN region maintains the expression of SOX2 that is present in hPSCs, the early NN regions induce expression of markers like GATA3 [8]. Consequently, we employed GATA3 as a marker of differentiation towards cell fates reflecting the early NN regions of the developing ectoderm and given our results of the Nodal-dependent switch in the gastrulation- versus preneurulation-associated gene profiles (Fig 3D), we hypothesized that a pSMAD1 gradient in the presence of Nodal signalling could pattern gastrulation-associated fates and in the absence of Nodal signalling would give rise to preneurulation-associated fates. To test this signalling logic, we sought to characterize the average activation of SMAD1 and SMAD2 (the effector of Nodal signalling) in BMP4 treated geometrically confined colonies in the presence or absence of Nodal signalling and query the correlation with the emergent fates. We treated 500 μm diameter hPSC colonies with 25 ng/ml of BMP4 for 24 h either in the presence of Nodal or SB and stained for either pSMAD1, SMAD2, and BRA (in the case of Nodal supplementation) or pSMAD1, pSMAD2, and GATA3 (in the case of SB supplementation). Consistent with our expected results, we observed that whereas a pSMAD1 signalling gradient organized in both experimental conditions—albeit with different shapes as expected—immunofluorescent data showed that SMAD2 activity was restricted to the condition in which Nodal was supplemented in the induction medium and absent in the presence of SB (S11 Fig). In addition, in conditions in which SMAD2 activity was permitted resulted in emergence of BRA expression at the periphery (S11A Fig, S11B Fig, S11C Fig, S12A Fig and S12B Fig) and inhibition of SMAD2 activity was correlated with the emergence of GATA3 at the colony periphery (S11D–S11F Fig). Notably, although the expression of GATA3 at the colony periphery in the condition when Nodal was inhibited was robust, the expression of BRA in the condition when Nodal signalling was permitted was modest (S11A–S11C Fig). Given that the pSMAD1 levels at the colony periphery are lower when the induction media are supplemented with Nodal relative to SB, these results are consistent with our previous study in which we reported that consistent with the PI paradigm, expression of the peri-gastrulation–associated fates emerge as a function of BMP4 dose and the induction time [24]. Indeed, when we tested the expression of BRA after a 36 h induction in the identical medium composition, we observed robust expression of BRA as expected (S12C–S12E Fig). These data provide further evidence to the notion that the patterned fates arise in a manner consistent with PI whereby patterned fates arise as a function of morphogen concentration and induction time. Furthermore, the observation that GATA3 expression emerged faster than BRA expression is also consistent with the signalling-threshold–dependent fate patterning. This is because although the dose of BMP4 ligands in both these conditions was identical, in the absence of Nodal signalling, the colonies experience higher levels of pSMAD1 activity (Fig 5A–5C) likely due to increased levels of BMP activators (Fig 5D) and reduced levels of BMP antagonists (Fig 5E, and S10H and S10I Fig).

We next asked whether the changes in the shape of the pSMAD1 signalling gradients that we observed as a function of Nodal signalling (Fig 5A–5C, S10A–S10C Fig) could be computationally predicted through a mathematical model of the RD paradigm that we propose. To test this, we employed the mathematical model we previously reported of a simplified model of BMP4-Noggin dependent organization of pSMAD1 signalling [24] and included an inhibitory regulation of BMP activity mediated by BMP antagonists downstream of Nodal signalling (please see S1 Text). We modelled the spatial profile of this inhibitory function after the SMAD2 profile observed at 24 h in media conditions permissive of Nodal signalling (S11A–S11C Fig). Notably, not only was this RD model able to predict the differences in the shape of pSMAD1 between the conditions when Nodal was present versus absent for both doses of BMP4 tested, but the model also predicted the recovery of BMP signalling in the centre of the colony when the geometrically confined colonies were treated with 25 ng/ml of BMP4 in the presence of Nodal ligands (S13 Fig). These data show that a simplified RD model is sufficient to explain the Nodal signalling-dependent changes in shape observed in the pSMAD1 signalling.

To provide further evidence for the role that Nodal might play in the organization of pSMAD1 signalling levels and switching the cell fates that appear in the differentiating hPSC colonies we next asked whether we could observe in situ stains of the key genes (like Nodal and CER1) switch in a manner that mirrored the activation or inhibition of Nodal signalling. Given that we observed robust expression of BRA after a 36 h induction (S12A Fig and S12B Fig) when the hPSC colonies were treated with 25 ng/ml of BMP4, we opted to employ this time point to test for Nodal, CER1, and GDF3 expression between the induction conditions that either permitted Nodal signalling or inhibited it. Consistent with our hypothesis, whereas these genes were robustly expressed in the presence of Nodal at regions that closely mirror the observed repression of pSMAD1 signalling (Fig 5A–5C and S14 Fig) and in close vicinity of the expression of gastrulation-associated fates like BRA (S12A Fig, S12B Fig and S14 Fig), they were not detected when the induction medium was supplemented with SB (S14 Fig).

We next asked if these observations of Nodal signalling-dependent switch in cell fates could be observed for other cardinal peri-gastrulation–and preneurulation-associated markers and sought to assay for markers specific to these developmental stages after 48 h treatment with 25 ng/ml of BMP4 in media supplemented with either Nodal or SB. Consistent with the requirement of Nodal signalling for the specification of gastrulation-associated fates [24], despite the presence of a pSMAD1 signalling gradient in the absence of Nodal signalling after 24 h of BMP4 treatment (Figs 4 and 5), we did not observe expression of gastrulation-associated markers like BRA, EOMES, SOX17, and GATA6 (S15A Fig and S15B Fig). Markers like TFAP2A mark the NN, NNE, and maturing NPB region that marks the NC fate; and SIX1 are expressed in the maturing NPB region, which marks panplacodal competent tissues [8]. Consistent with our observation that BMP4 treatment in the absence of Nodal signalling up-regulated genes associated with preneurulation, we observed spatially segregated expression of SOX2 (PN) and GATA3 (NN) with concomitant expression of TFAP2A (NN, NPB) and SIX1 (panplacodal competent NPB) (S15C Fig). We define this fate patterning as ‘pre-neurulation-like’, and using SOX2 and GATA3 as the markers of the PN and NN tissues, we set out to test whether the fate patterning arose in a manner consistent with the PI paradigm.

#### Preneurulation-like fates pattern in a manner consistent with PI

**Fig 6:**
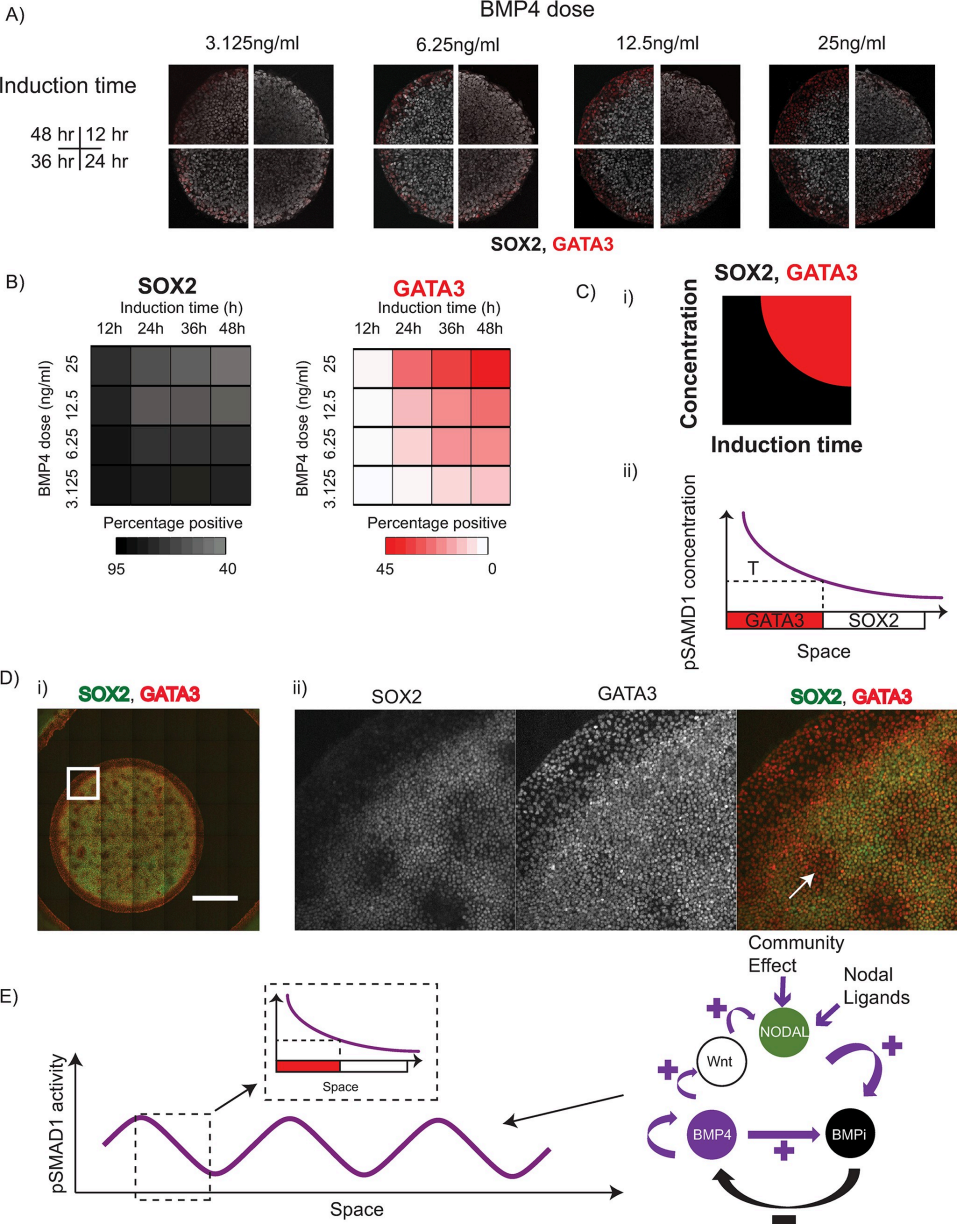
Preneurulation-like fates arise in a manner consistent with PI. (A) Representative immunofluorescence images of 500 μm diameter colonies stained for SOX2 and GATA3 after different doses (6.25 ng/ml, 12.5 ng/ml, 25 ng/ml, and 50 ng/ml) and times of BMP4 treatment. Scale bar represents 200 μm. (B) Mean expression levels of SOX2 and GATA 3 represented as heat maps. Darker shades represent higher expression levels, and lighter shades represent lower levels of expression (for detailed data see S17 Fig). (Ci–ii) Model of GATA3 patterning. (i) Overview of (B) in which GATA3 is expressed as a function of BMP4 dose and induction time. (ii) Fate patterning of GATA3 consistent with PI. ‘T’ indicates the presumptive threshold of fate switch to GATA3. (D) Treatment of geometrically confined hPSC colonies of 3 mm diameter with 200 ng/ml of BMP4 and SB for 48 h results in multiple peaks of GATA3 expressing regions consistent with RD hypothesis. (i) Representative stitched images of 3 mm diameter hPSC colonies differentiated with 200 ng/ml of BMP4 for 48 h. Scale bar represents 1 mm. (ii) Zoomed section outlined by the white square in (i). White arrows indicate regions of high GATA3 and low SOX2 expression indicative of PI-mediated fate patterning due to presumptive localized pSMAD1 expression (please see S22 Fig). The experiment was repeated 3 times. Additional images shown in S25 Fig. (E) Overall model of pSMAD1 signalling organization governed by a reaction-diffusion–like network that contains BMP, Nodal, and BMP antagonists like Noggin, and CER1, and the fate patterning due to the signalling gradient arises in a manner consistent with PI. Underlying numerical data for this figure can be found in https://osf.io/zrvxj/. BMP4, bone morphogenetic protein 4; CER1, Cerberus; GATA3, GATA binding factor 3; hPSC, human pluripotent stem cell; PI, positional information; pSMAD1, phosphorylated SMAD1; RD, reaction-diffusion; SB, SB431542 (Nodal signalling antagonist); SOX2, SRY-box transcription factor 2.

Given that the PI paradigm posits that developmental fates arise because of thresholds of morphogen levels, we asked whether the perturbations of pSMAD1 levels at the colony periphery (S9A–S9C Fig) and the colony centre (S9D Fig and S9E Fig) resulted in pSMAD1 threshold mediated changes in expression of GATA3 (NN) and SOX2 (PN) fates, respectively. Consistent with the idea of a pSMAD1 threshold dependent patterning of the PN and the NN tissues marked by GATA3 and SOX2, we find that reducing the pSMAD1 levels at the colony periphery (S16A Fig) significantly reduced the GATA3 expression at the colony periphery (S16B Fig and S16C Fig) and increasing the pSMAD1 levels at the colony centre (S16D Fig) dramatically reduced the SOX2 expression (S16E Fig and S16F Fig). These data indicate that thresholds of pSMAD1 regulated the patterning of the SOX2 and GATA3 within the geometrically confined hPSC colonies. However, the formalization of the PI paradigm has been updated to include time as a critical parameter that patterns the developmental cell fates. Specifically, fate patterning mediated by PI is known to arise as a function of the morphogen concentration and time of induction [17,18,24,56]. Consequently, we tested 4 different doses of BMP4 (3.125 ng/ml, 6.25 ng/ml, 12.5 ng/ml, and 25 ng/ml) in the induction medium for 4 different induction times (12 h, 24 h, 36 h, and 48 h) and measured the levels of SOX2 and GATA3 detected. We observed that the fate patterning of GATA3 arose as a function of both the concentration of BMP4 in the induction medium and the time of induction (Fig 6A, 6B and 6Ci, and S17 Fig), indicating that the patterning within the geometrically confined colonies arises in a manner consistent with PI (Fig 6Cii).

### A coordinated model of RD and PI governs preneurulation-like fate patterning

Thus far, our data indicate that the pSMAD1 gradient was enforced outside-in within the geometrically confined hPSC colonies via a BMP4-Noggin RD network, and the preneurulation-like fates arose in a manner consistent with PI. In agreement with this idea, perturbing the shapes of the geometrically confined hPSC colonies did not result in fate patterning that deviated from the expected results (S18 Fig). However, a strong test of this overall model is asking whether large colonies are able to generate stereotypical RD-like periodic signalling and fate profile. We previously reported that treatment of large geometrically confined hPSC colonies (3 mm) with high doses of BMP4 would give rise to multiple foci of BMP activity as indicated by pSMAD1 staining and patterned gastrulation-associated fates [24]—consistent with possible spatial oscillations that are predicted to arise in accordance with the RD paradigm. Surprisingly, when we tested the response of pSMAD1 spatial signalling dynamics in 3 mm diameter colonies after BMP4 and SB treatment for 24 h, we did not observe any obvious additional foci at either 50 ng/ml (S19 Fig) or 200 ng/ml (S20 Fig) BMP4 dose. Of note, the medium used for differentiating these geometrically confined hPSC colonies contained knockout serum replacement (SR). An ingredient of SR called AlbumaxII is not well defined and is known to contain lipid-associated proteins that have been shown to have an effect on hPSC biology—mechanisms of which are currently unclear [57,58]. We asked whether using medium devoid of SR would rescue the expected appearance of multiple foci of pSMAD1 activity and GATA3 expression consistent with the predictions of the RD paradigm [24]. Indeed, when we tested N2B27 medium that does not contain any AlbumaxII or SR (see Materials and methods for composition), a 24 h BMP4 and SB treatment of hPSC colonies of 3 mm diameter resulted in rudimentary peaks of pSMAD1 activity at a BMP4 dose of 50 ng/ml (S21 Fig) and prominent peaks of pSMAD1 activity at a dose of 200 ng/ml (S22 Fig). In addition, after 48 h of BMP4 and SB treatment, although we did not note any additional foci of GATA3 in SR medium even when supplemented with doses of 200 ng/ml (S23 Fig), in an N2B27 basal medium supplemented with SB, rudimentary peaks of GATA3 expression were observed at 50 ng/ml of BMP4 (S24 Fig), and robust peaks of GATA3 expression were noted at 200 ng/ml of BMP4 (Fig 6D and S25 Fig). Consistent with the idea that these peaks were due to foci of BMP signalling peaks, regions that were positive for GATA3 were negative for SOX2, which is known to be potently inhibited by BMP signalling [30]. We sought to quantitatively assess whether the observed BMP signalling peaks detected in our experiments had an underlying structure to the profile, or whether these patterns lacked any order and represented random distributions. To do this, we applied information dynamics measures to study the distribution of nearest neighbours of the peaks of BMP signalling activity observed in our experimental data and compared them with 2 test profiles—patterns that were generated using a random distribution generator, and patterns extracted from our RD model. For the peaks in the experimental data, we employed the SOX2 negative regions observed in the 3 mm diameter colonies treated with 200 ng/ml of BMP4 in N2B27 supplemented with SB because of the superior reliability of thresholding the negative and positive staining in immunofluorescent images of SOX2 relative to the staining for pSMAD1 or GATA3. We found that the distributions from the experimental data were significantly different from the distributions observed in the randomly generated profiles indicating that the spatial profile of BMP activity peaks had an inherent order to them. Interestingly, however, the distributions of the distances to the nearest neighbours observed in the experimental data also deviated from exact periodic patterns, as expected from the idealized approximations that we employed in our model. Overall, our analyses indicate that although the underlying structure of the BMP signalling peaks observed in our experimental data do not exactly mirror those observed in our simplified and deterministic mathematical model, they contain an inherent structure and as such are not random (please see S1 Text).

These observations are consistent with the proposition that an RD network organizes BMP signalling activity (Fig 6E) and the patterned fates arise in a manner consistent with PI (Fig 6C), although we note that undefined components (such as AlbumaxII in SR) present in the induction medium may contribute to deviations from the expected results.

### Preneural and NN regions give rise to definitive ectodermal fates

**Fig 7:**
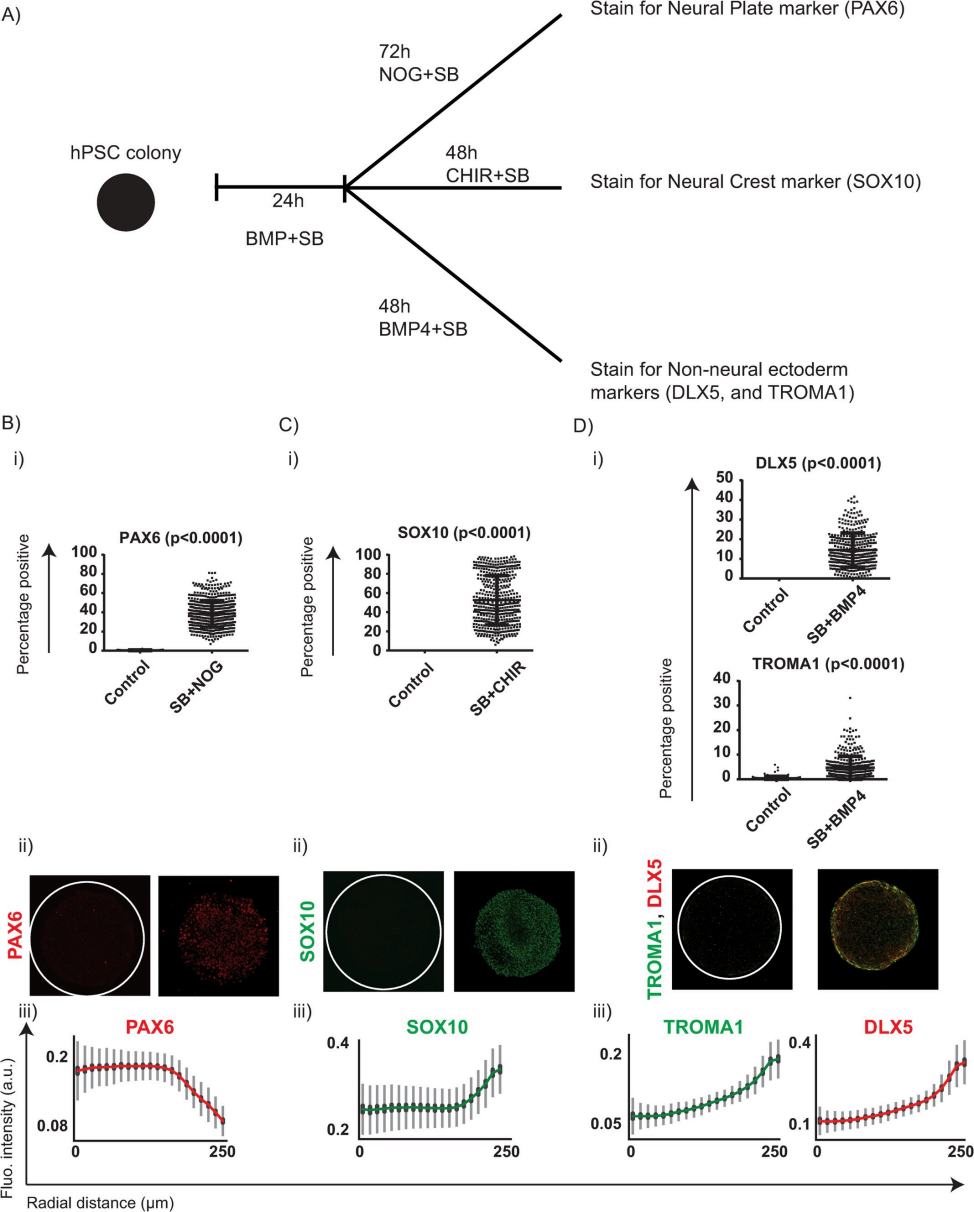
Preneurulation-like platform can give rise to fates associated with the differentiating ectoderm. (A) Overview of the experimental setup. Geometrically confined hPSC colonies were treated with SB and BMP4 for 24 h and then treated with one of the following conditions: SB and Noggin for 72 h and subsequently stained for PAX6; SB and CHIR99021 (CHIR) for 48 h and stained for SOX10; SB and BMP4 for 48 h and stained for DLX5 and TROMA1. (B) Expression of NP marker (PAX6) in colonies differentiated with BMP4 for 24 h and SB + Noggin for 72 h. (i) Quantified expression observed for PAX6 observed in the treated and control conditions. The number of colonies were 76 for control and 606 for treated. (ii) Immunofluorescent images of representative colonies from panel B(i) stained for PAX6, and (iii) the spatial profile of PAX6 observed in the condition of SB + NOG in panel B(i) represented as a line plot. SDs shown in grey, and 95% confidence intervals shown in black. (C) Expression of NC marker (SOX10) in colonies differentiated with BMP4 for 24 h and SB + CHIR for 48 h. (i) Quantified expression observed for SOX10 observed in the treated and control conditions. The number of colonies were 286 for control and 493 for treated. (ii) Immunofluorescent images of representative colonies stained for SOX10 and (iii) the spatial profile of SOX10 observed in the condition of SB + CHIR in panel C(i) represented as a line plot. SDs shown in grey, and 95% confidence intervals shown in black. (D) Expression of NNE markers (DLX5 and TROMA1) in colonies differentiated with BMP4 for 24 h and SB + BMP4 for 48 h. (i) Quantified expression observed for DLX5 and TROMA1 observed in the treated and control conditions. The number of colonies were 163 for control and 376 for treated. (ii) Immunofluorescent images of representative colonies stained for DLX5 and TROMA1 and (iii) the spatial profile of DLX5 and TROMA1 observed in the condition of SB + BMP4 in panel D(i) represented as a line plot. SDs shown in grey, and 95% confidence intervals shown in black. For panels B(i), C(i), and D(i), each data point represents an identified colony (as per the analysis pipeline explained in the Materials and methods), and bars represent mean ± SD. The data were pooled from 2 experiments, and the *p*-values were measured using Mann-Whitney U test. Please see S26 Fig for associated DAPI images. Underlying numerical data for this figure can be found in https://osf.io/zrvxj/. BMP4, bone morphogenetic protein 4; DLX5, Distal-Less Homeobox 5; hPSC, human pluripotent stem cell; NC, neural crest; NNE, NN ectoderm; NOG, Noggin; NP, neural plate; PAX6,; SB, SB431542 (Nodal signalling antagonist); SOX10, SRY-box transcription factor 10; TROMA1, cytokeratin-8 antibody clone.

As a final validation that BMP4 and SB treatment induced preneurulation-associated fates in the differentiating geometrically confined hPSC colonies, we asked whether the patterned fates of the early PN and NN tissues were capable of inducing marker expression of definitive ectodermal fates like the NP, the NC, and the NNE. During embryogenesis, the NNE specifies toward the lateral end of the medial-lateral axis because of sustained levels of high BMP signalling in the ectoderm; the NC fate is specified at regions of intermediate BMP levels that activate Wnt signalling; and the NP is specified at the medial end where the tissue is subject to low/no BMP signalling. To test the competence of the preneurulation-like patterned colonies to give rise to these fates, we treated the colonies with BMP4 and SB for 24 h, then tested 3 different treatments. Specifically, we either treated the colonies for a further 48 h with BMP and SB and stained for keratins using a pan-keratin antibody and DLX5 (markers of NNE) or CHIR99021 (‘CHIR’, a wnt agonist) and SB and stained for SOX10 (a marker of the NC fate) or for a period of 72 h with Noggin and SB and stained for PAX6 (Fig 7A). Consistent with our expected results, we observed that the colonies treated with Noggin and SB expressed PAX6—a bona fide marker of the NP (Fig 7B and S26 Fig); robust SOX10 staining was observed in colonies treated with CHIR and SB (Fig 7C and S26 Fig), and sustained BMP4 and SB treatment resulted in robust expression of DLX5 and showed clear staining of a pan-keratin antibody, indicating acquisition of a NNE identity (Fig 7D and S26 Fig). A recent elegant study, Xue and colleagues reported a protocol of generating hPSC colonies with regionalized fate compartments that expressed bona fide NPB markers like ZIC1, MSX1, and SOX10 [27]. Xue and colleagues developed a 9-day-long protocol in which they relied on biomechanical cues like cell size and cytoskeletal contractility to activate a BMP signalling gradient in geometrically confined hPSC colonies and provided a transient, 24 h pulse of Wnt signalling at day 3. To provide further evidence that the NN compartment in our platform was able to give rise to definitive ectodermal cells, we tested a hybrid protocol between the one we report and the one Xue and colleagues employed and asked whether we could observe the bona fide markers of the NPB in these colonies. We first induced 200 μm diameter hPSC colonies with a short 24 h pulse of BMP4 in medium supplemented with SB to generate a GATA3 positive population in the colony periphery (S27A Fig). Notably, this short pulse robustly induced GATA3 expression at both 12.5 ng/ml and 25 ng/ml of BMP4 significantly higher than the induction of CDX2 observed in these colonies (S27B Fig–S27E Fig). Having established a robust expression of GATA3 in the first 24 h of BMP4 treatment, we next tested an additional 3-day differentiation protocol that contained a 24 h pulse of Wnt signalling (3μM CHIR) between D2 and D3 (S28A Fig). Consistent with the idea that the GATA3 positive population was competent to give rise to the NPB fates, we observed robust expression of neural plate border markers like ZIC1, MSX1, and SOX10 at the end of D4 (S28B Fig and S28C Fig).

**Fig 8:**
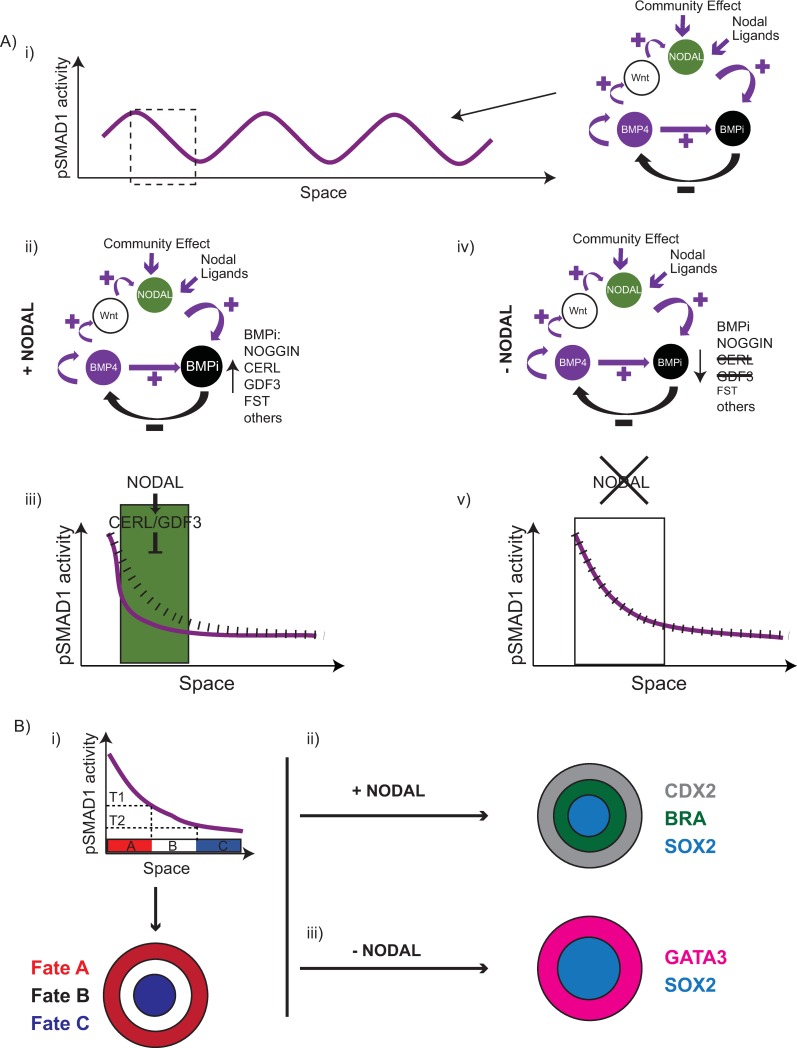
Mechanism of Nodal-dependent fate patterning in the geometrically confined hPSC colonies. (A) Overall proposed model of Nodal-mediated fate patterning regulation in geometrically confined hPSC colonies. (i) Model overview for organization of pSMAD1: An RD network in BMP signalling that comprises BMP ligands, Nodal (via BMP-Wnt-Nodal axis), and BMP antagonists organizes the pSMAD1 gradient within the geometrically confined hPSC colonies. (ii–iii) pSMAD1 organization in the presence of Nodal. (ii) BMP antagonists downstream of Nodal signalling (like CER1, GDF3, etc.) can contribute to the organization of the pSMAD1 gradient resulting in enhanced inhibitor activity. (iii) The presence of BMP antagonists in the Nodal pathway down-regulates pSMAD1 levels enforcing a sharp gradient from the colony periphery. Green region signifies region of active Nodal where down-regulation of BMP signalling is more pronounced. Dashed black line represents gradient that would arise in the absence of BMP antagonists in the Nodal pathway. Purple line represents gradient established due to pronounced inhibition of BMP signalling. (iv–v) pSMAD1 organization in the absence of Nodal. (iv) In the absence of Nodal signalling the overall level of BMP inhibitors is reduced because of removal of CER1, GDF3, and reduction in FST levels. (v) The established gradient is more gradual relative to when Nodal signalling is active. Dashed black line represents gradient that would arise in the absence of BMP antagonists in the Nodal pathway. Purple line represents gradient established independent of BMP antagonists downstream of Nodal signalling. (B) Fate patterning mediated by pSMAD1 signalling gradient. (i) Fate patterning in response to the pSMAD1 signalling gradient is consistent with PI. (ii) In the presence of Nodal signalling the fate patterning gives rise to BRA—a gastrulation-associated fate. (iii) In the absence of Nodal signalling the fate patterning gives rise to GATA3—a preneurulation-associated fate. BMP, bone morphogenetic protein; BMPi, bone morphogenetic protein inhibitors; BRA, Brachyury; CER1, Cerberus; FST, Follistatin; GATA3, GATA binding protein 3; GDF3, growth differentiation factor 3; hPSC, human pluripotent stem cell; pSMAD1, phosphorylated SMAD1; RD, reaction-diffusion.

Taken together, our data are consistent with our hypothesis that a RD network in BMP signalling organizes the pSMAD1 gradient in geometrically confined hPSC colonies and Nodal signalling bisects peri-gastrulation–associated and preneurulation-associated fates (Fig 8) that arise within these colonies in a manner consistent with PI.

## Discussion

### Screening platform for quantitative assessment of hPSC differentiation propensity

To capitalize on the well-established promise of hPSCs [59], many groups and initiatives have banked large numbers of human-induced (hi)PSCs for future use in regenerative medicine applications [28]. Importantly, it is widely recognized that different hiPSC lines—even if derived from identical genetic and tissue backgrounds—significantly vary in their ability to induce certain cell fates [28–30]. Consequently, the field needs an assay that enables rapid, high-content quantification of lineage bias of a starting pool of hPSCs from which an ideal line would be chosen to produce cells of a target fate. The peri-gastrulation–like assay represents a quantitative and rapid option that can assess the propensity of hPSC lines to differentiate to specific lineages.

In addition to the variation in BRA expression observed in the peri-gastrulation–like assay, we demonstrate that Wnt3a and ActivinA treatment of geometrically confined hPSC colonies of different hPSC lines results in variable endoderm induction efficiencies that mirror the predicted propensity both from directed differentiation towards definitive endoderm and as indicated by the temporal dynamics of MIXL1 and EOMES in the EB assay. Notably, this observation parallels the observation reported by Kojima and colleagues who showed that the temporal dynamics of MIXL1 during undirected differentiation of mEpiSC lines predicted endodermal differentiation propensity [47]. Consequently, we both corroborate the approach taken by Kojima and colleagues in the human system and provide proof-of-concept data that indicates that morphogen treatment of geometrically confined hPSC colonies in defined conditions might represent a rapid, quantitative, and inexpensive solution for finger-printing hPSCs.

### Involvement of Nodal in RD and PI associated with the BMP signalling

Although both RD and PI have been studied in a variety of different model systems and have provided much insight into how developmental fate patterning occurs during embryogenesis, the question of how multiple different signalling pathways can work in concert to execute the rules associated with each paradigm remains unclear. In this study, we demonstrate that Nodal signalling works in concert with BMP signalling to not only orchestrate the organization of pSMAD1 activity into a signalling gradient in a manner consistent with RD but also coordinates the interpretation of the gradient into either peri-gastrulation–like or preneurulation-like fate patterns in accordance with PI.

In a recent study, we proposed that the pSMAD1 signalling gradient organizes under the regulation of a BMP4-Noggin RD system [24]. Our data in this study demonstrate that the pSMAD1 gradient formed in the presence and absence of Nodal signalling is significantly different (Fig 5 and S10 Fig), indicating the necessity of updating that proposed RD network topology to include Nodal signalling which is activated downstream of BMP signalling during the onset of mammalian gastrulation. Our data also suggest that the role played by Nodal signalling may, at least in part, be enacted by BMP antagonists that are targets of Nodal signalling, which is consistent with the fact that RD-mediated organization of morphogen signalling relies on the function of the morphogen inhibitors [11,12]. For instance, a Nodal signalling gradient during zebrafish embryogenesis forms because of a Nodal-Lefty RD system [60], and Rodgers and colleagues have demonstrated that removing Lefty in zebrafish embryos results in an increased amount of Nodal signalling and specification of mesendoderm in the embryos during gastrulation [61]. Under experimental conditions in which Nodal signalling is inhibited because of SB supplementation, Noggin, Follistatin (S10I Fig), Gremlin family proteins [26], and possibly others can act as the inhibitors that enforce the pSMAD1 signalling gradient; and in conditions permissive of Nodal signalling, CER1 (Fig 5E) and GDF3 (S10H Fig) and possibly others can further antagonize BMP signalling. In support of this notion, a previous study has reported that siRNA mediated inhibition of CER1 and Lefty in experimental conditions permissive of Nodal signalling dramatically compromises the formation of peri-gastrulation–associated patterns [25]. Furthermore, in our previous study, we observed patterned foci of pSMAD1 at 24 h and of BRA at 48 h in the 3 mm diameter colonies when the induction media contained 200 ng/ml of BMP4 but not when the medium contained 50 ng/ml of BMP4 [24]. However, in this study, when we tested the formation of these patterned foci, we also observed them at 50 ng/ml of BMP4. Given that increasing or decreasing the levels of inhibitors in an RD system would function to, respectively, decrease or increase the function of the activators—which has been shown to be the case in the WNT-DKK mediated patterning of hair follicles [62], we hypothesize that in the absence of Nodal signalling, hPSCs display heightened sensitivity to manifest RD-like peaks of BMP activity due to a reduced pool of BMP antagonists that orchestrate the organization of the BMP signalling gradient. Taken together, the topology of the RD network in BMP signalling needs to incorporate the role played by Nodal and potentially multiple BMP4 antagonists in addition to Noggin (like CER1, GDF3, FST among others). A deeper and more comprehensive understanding of the RD network in the BMP pathway requires further careful studies and computational platforms that enable studying multiple nodes in RD networks will be very valuable [14].

In Nodal-permissive experimental conditions, BMP4 treatment of geometrically confined hPSC colonies results in a gradient that down-regulates sharply (S10B Fig and S10C Fig) [24–26]. This has led to a proposition that in BMP4-treated geometrically confined hPSC colonies, BMP signalling is active exclusively at the colony periphery and inactive everywhere else—a spatial profile that can be modelled as a step-function along the colony radius [63]. These authors claim that this apparent step-function–like response in the pSMAD1 activity occurs because of negative feedback enforced on BMP signalling at 2 different levels. First, by a BMP signalling mediated up-regulation of BMP inhibitors [24,26], and second, due to a cell density mediated relocalization of BMP receptors from being present apically to becoming localized at basolateral regions, rendering them inaccessible for ligand mediated activation, everywhere in the colony except for the periphery [26]. Consistent with their proposed model, we also identify a valuable role of BMP inhibitors in orchestrating the pSMAD1 signalling gradient. However, our data indicate that the underlying mechanism regulating the pSMAD1 organization is inconsistent with a wide-spread dampening of BMP signalling owing to inaccessible BMP receptors. In this study, in addition to the unambiguous RD-like spatial expression patterns of pSMAD1 and differentiation markers like GATA3, in large colonies (3 mm diameter), we identify experimental conditions that result in prominent expression of pSMAD1 at the periphery and the centre of hPSC colonies of 500 μm diameter when treated with 25 ng/ml of BMP4 for 24 h—observations consistent with RD-like behaviour (Fig 5A–5C). Neither of these observed expression profiles would arise in conditions where BMP receptors were inaccessible everywhere except for the colony periphery. Additional insight has been provided by Xue and colleagues [27] in which it was demonstrated that when treated with BMP4 for an extended period of time (4 days), all cells in the hPSC colony expressed nuclear-localized pSMAD1 (Fig 5A and 5B in ref. 27). We propose an alternative hypothesis for both the observed density dependent dampening and the apparent step-function–like activity of BMP signalling observed in this system that invokes the involvement of Nodal signalling. Nodal is known to have a community effect whereby endogenous Nodal levels become more pronounced at higher cell densities [64,65], which would up-regulate BMP antagonists downstream of Nodal signalling [52]. We argue that at increasing cell densities in the differentiating hPSC colonies, along with an increase in levels of BMP antagonists like Noggin and Gremlin family proteins owing to more cells secreting these inhibitors, there would likely be a dramatic increase in the levels of BMP antagonists like CER1, GDF3, FST, which would up-regulate because of pronounced levels of a community-effect mediated increase in endogenous Nodal. Abrogation of the fate patterning in this system in response to siRNA mediated inhibition of CER1 and LEFTY—an experimental condition that in principle should neither interfere with Noggin expression nor change colony density, also supports our interpretation. Finally, our study provides direct evidence for the involvement of Nodal signalling in the apparent step-function–like response in pSMAD1 expression along the colony radius. When colonies of 500 μm diameter were treated with 25 ng/ml of BMP4 and SB, the signalling gradient formed showed no step-like response in the spatial pSMAD1 profile. Instead the signalling gradient gradually decreased in strength (as indicated by pSMAD1 fluorescence levels) from the colony periphery to the colony centre (Fig 5A–5C). However, in the presence of Nodal signalling, the pSMAD1 signalling gradient, indeed, down-regulated sharply—as expected from a step-function-like response. This is consistent with the proposition that in regions within the differentiating geometrically confined hPSC colonies that have active Nodal signalling, which have been shown to be immediately interior to the peripheral cells [26], there would be heightened levels of BMP antagonists like CER1 causing a dramatic reduction of BMP signalling.

Notably, in our study, we report different aspects that can result in variability in experimental results when studying the stereotypic RD-like periodic response in BMP signalling in the hPSC context. One such source of variability is the level of endogenous Nodal signalling between different hPSC lines (Fig 3C). Given the role that Nodal signalling plays in the formation of the pSMAD1 gradient (Fig 5 and S10 Fig), and the critical role it plays in ensuring the peri-gastrulation-associated fate patterning [24], the variability in endogenous levels of Nodal signalling can cause inconsistent responses between different cell lines and culture conditions. Importantly, a recent study has also shown drastically different responses in endogenous Nodal activation within the same hPSC line when cultured under different conditions for routine maintenance [66]—highlighting that even culture conditions for routine hPSC maintenance can have an effect in the response in the peri-gastrulation–like assay. Secondly, we observed that when geometrically confined hPSC colonies of 3 mm diameter were treated with a high dose of BMP4 in SR medium, the stereotypical RD-like spatial periodicity of either pSMAD1 or GATA3 were not readily observed. However, changing the medium to an N2B27-based medium rescued these periodic responses. A key component of SR medium is Knockout Serum Replacement (KSR) which is known to contain lipid-associated proteins like lysophosphatidic acid (LPA), and although the mechanism of action remains unclear, molecules like LPA have been shown to have an inhibitory effect on hPSC differentiation [57,58]. Given the above caveats associated with in vitro experiments, studies aimed at investigating the details of the RD network in BMP signalling in the hPSC context—especially those directed toward investigating the specifics of the spatial periodicity of morphogen activity and fate patterning—would benefit from removing these sources of variability between hPSC lines. Employing basal medium like N2B27 which is devoid of components like AlbumaxII and LPA, avoiding undefined media like those conditioned on MEFs and removing Nodal signalling from their system by SB supplementation represent experimental conditions better suited for these studies.

We developed a mathematical model that robustly predicts our experimental results by using our previously reported computational model of an RD-mediated interaction between BMP4 and Noggin [24] and updated it by incorporating the involvement of Nodal signalling (S1 Text). Nodal activation may occur via multiple independent routes—by the presence of Nodal ligands in the induction medium, activation of Nodal via the BMP-Wnt-Nodal axis associated with gastrulation, or via community effect (Fig 8). As such, rather than disentangle the relative contributions of each route, we chose the spatial profile of Nodal-signalling–mediated inhibition of BMP based on empirical observations (S11A Fig). Notably, much like the formation of the pSMAD1 gradient (Fig 4B–4D), the formation of the SMAD2/pSMAD2 profile is likely also formed in a dynamic manner, and the fact that we opted to employ an empirically determined static profile of Nodal signalling limits generalization of our approach. To address this concern, we tested the response of the model to dynamically changing levels of SMAD2/pSMAD2 and found that the model outputs were robust to the parameters chosen (S1 Text). It is noteworthy, however, that this approach does not provide insight into how the interactions from the 3 activation routes lead to the formation of the Nodal signalling profile. This represents an important question and investigating the complex underlying regulation requires further work.

An alternative possibility to the RD mechanism underlying the formation of the GATA3 positive foci in a larger SOX2 positive space is that the starting populations contain both these cell populations which then separate out owing to differential adhesive properties—a model called ‘phase separation’ [67]. This idea was tested by Cachat and colleagues where they constructed 2 weakly adhesive populations which could be chemically induced to express 2 different cadherins (E-Cadherin and P-Cadherin) [68]. This conferred to each population a preferential affinity for itself instead of the population expressing the other cadherin. They observed the emergence of complex patterns in both 2D colonies and 3D aggregates. Interestingly, however, reducing the size of these clusters resulted in complete separation of the populations into 2 halves containing one population each. These observations are inconsistent with the results from our experiments when we reduce the colony sizes (S16 Fig). Instead, under these experimental conditions, our observations remain consistent with the RD hypothesis. Furthermore, in conditions when the colonies are not treated with BMP4, we do not observe any formation of spots or stripes that would be predicted to emerge whether the patterns were arising because of heterogeneity present in the starting population that spatially separate owing to differential adhesive affinities (Fig 1D) [69–71].

### Conclusions

In conclusion, we report the characterization of a high-throughput microtiter plate that enables robust geometric confinement of a variety of cell types. We employ this platform to screen hPSC lines for their ability to induce gastrulation-associated fate patterning and observe a Nodal-dependent response in the efficiency of BRA (a gastrulation-associated fate) induction, thereby providing a proof of principle of the ability of this platform to be employed for high-throughput screening experiments. In addition, we identify that differentiating hPSCs up-regulate either gastrulation or preneurulation-associated gene profiles in a Nodal signalling-dependent manner. Further, we demonstrate that in BMP4 treated geometrically confined hPSC colonies, Nodal signalling can affect the RD-mediated organization of pSMAD1—the downstream effector of BMP signalling and that it also regulates the switch between peri-gastrulation–like and preneurulation-like identities in PI-mediated fate patterning occurring within the differentiating colonies. We also report a computational model that predicts the effect of Nodal signalling in the formation of the pSMAD1 signalling gradient. Finally, consistent with a previous study that investigated peri-gastrulation–like fate patterning in BMP4 treated hPSC colonies, we demonstrate that the preneurulation-like fate patterning follows a coordinated model of RD and PI, hinting at possible conservation of the underlying mechanism that regulates differentiation of the epiblast and ectoderm in human development.

## Materials and methods

### Human pluripotent stem cell culture

CA1 human embryonic stem cell line was provided by Dr. Andras Nagy (Samuel Lunenfeld Research Institute). H9-1 was provided by Dr. Sean Palecek (University of Wisconsin–Madison). H9-2, HES2, and MEL1 (PDX1-GFP) were provided by Dr. Gordon Keller (McEwen Centre for Regenerative Medicine/University Health Network). HES3-1, and HES3-2 were provided by Dr. Andrew Elefanty (Monash University). H1, H7, H9-3 were acquired from WiCell Research Institute. For routine maintenance, CA1, H9-1, and H9-2 were cultured on diluted (at 1:50) Geltrex (Life Technologies, Carlsbad, California) coated 6-well tissue culture plates using mTeSR1 medium (StemCell Technologies, Vancouver, British Columbia, Canada) as per manufacturer’s instructions. The cells were passaged at a ratio of 1:12 using ReleSR (StemCell Technologies, Vancouver, British Columbia, Canada) per manufacturer’s instructions. For the first 24 h after passage, the cells were cultured in ROCK inhibitor Y-27632 to increase cell viability. The medium was changed every day and passaged every 4 to 5 days or when the cells reached 75% to 80% confluence. For routine maintenance, H1, H7, H9-3, HES3-1, HES3-2, MEL1, and HES2 were cultured on feeder layers of irradiated MEFs in Dulbecco’s Modified Eagle’s Medium (DMEM; Invitrogen, Carlsbad, California), 1% Penicillin/Streptomycin, 1% non- essential amino acids, 0.1mM β-mercaptoethanol, 1% Glutamax, 2% B27 minus retinoic acid, 20% KSR (referred to as ‘SR’ medium), and supplemented with 20 ng ml−1 FGF-2 (PeproTech, Hammersmith, London, United Kingdom). H1, H7, and H9-3 cells were passaged 1:6 every 4 to 5 days and were disassociated into small clumps using 0.1% collagenase IV (Invitrogen, Carlsbad, California). HES3-1 and HES3-2 were passaged 1:24 every 4 to 5 days and dissociated using TryplE Express (Invitrogen, Carlsbad, California). All cell lines were confirmed negative for mycoplasma contamination.

### Preparation of PEG plates

Platform set up and XPS studies were performed using 22 mm × 22 mm borosilicate coverslips (Fisher Scientific, Hampton, New Hampshire), and the 96-well plate platform was developed using custom sized (110 mm × 74 mm) Nexterion-D Borosilicate thin glass coverslips (SCHOTT, Mainz, Germany). The glass coverslips were activated in a plasma cleaner (Herrick Plasma, Ithaca, New York) for 3 minutes at 700 mTorr and incubated with 1 ml of PLL-g-PEG (5 KD) (SUSOS, Dübendorf, Switzerland), at a concentration of 1 mg/ml at 37°C overnight. The glass slides were then rinsed with ddH_2_O and dried. The desired patterns were transferred to the surface of the PEG-coated side of the coverslip by photo-oxidizing select regions of the substrate using Deep UV exposure for 12 minutes through a Quartz photomask in a UV-Ozone cleaner (Jelight, Irvine, California). Multiple photomasks were employed in this study depending on the specific sizes and shapes of the geometrically confined colonies investigated. Bottomless 96-well plates were plasma treated for 3 minutes at 700 mTorr, and the patterned slides were glued to the bottomless plates to produce microtiter plates with patterned cell-culture surfaces. Adhesives validated for biocompatibility standards ISO10993, and USP Class VI were utilized for the assembly of the plates. Prior to seeding cells onto the plates, the wells were activated with N-(3-Dimethylaminopropyl)-N′-ethylcarbodiimide hydrochloride (Sigma, St. Louis, Missouri) and NHS (Sigma, St. Louis, Missouri) for 20 minutes. The plates were thoroughly washed 3 times with ddH_2_O and incubated with Geltrex (diluted 1:150) for 4 h at room temperature on an orbital shaker. After incubation, the plate was washed with Phosphate Buffered Saline (PBS) at least 3 times to get rid of any passively adsorbed extracellular matrix (ECM) and seeded with cells to develop micropatterned hPSC colonies.

### Comparison between PEG plates with μCP plates

PEG plates (as described above) and μCP plates (as reported previously [30]) were generated with patterned islands of 200 μm in diameter with 500 μm separation between adjacent colonies. A single-cell suspension of CA1s was generated by incubating in 1 ml of TryplE (Invitrogen, Carlsbad, California) per well for 3 minutes at 37°C. The TryplE was blocked using in equal volume SR medium (see ‘Human pluripotent stem cell culture’ section above for composition), and the cells were dissociated by pipetting to generate a single-cell suspension. The cells were centrifuged into a pellet, and the supernatant was aspirated to remove any residual TryplE. A single-cell suspension was then generated in SR medium supplemented with 10 μl of ROCKi and 20 ng/ml of bFGF at a cell density of 500,000 cells/ml and 100 μl of the suspension was plated onto the PEG and μCP plates for a period of 2 to 3 h till robust cell attachment was observed. When robust cell attachment was observed, ROCKi was removed from the media, and the cells were then left to make a confluent colony overnight (approximately 12 h). Once confluent colonies were observed, the differentiation was performed using 100 μl per well of the following inductive conditions in Apel (Stem Cell Technologies, Vancouver, British Columbia, Canada) basal media for 48 h—bFGF (40 ng/ml) +SB431542 (10 μM) to induce differentiation into ectodermal fates, BMP4 (10 ng/ml) +ActivinA (100 ng/ml) to induce mesendodermal differentiation, BMP4 (40 ng/ml) to induce extra-embryonic/’other’ fates, and as controls, Nutristem and basal Apel media were used. After 48 h, the colonies were fixed and stained for OCT4 and SOX2. The relative percentages of the colonies that were positive for the 2 markers were used to identify the early fates induced within the colonies. A detailed description of the assay has been previously reported [30].

### Peri-gastrulation–like and preneurulation-like fate patterning induction

Except for the experiments in which we demonstrated RD-like foci of pSMAD1 activity and the preneurulation-like fates in 3 mm diameter colonies, all fate patterning studies were performed in SR medium (see ‘Human pluripotent stem cell culture’ section above for composition) supplemented with 100 ng/ml of bFGF. The studies in which we demonstrate RD-like foci of the morphogen activity and fate patterning were performed in N2B27 medium. N2B27 medium was composed of 93% DMEM (Invitrogen, Carlsbad, California), 1% Penicillin/Streptomycin, 1% non-essential amino acids, 0.1 mM β-mercaptoethanol, 1% Glutamax, 1% N2 Supplement, and 2% B27 Supplement minus retinoic acid.

The hPSC lines that were cultured in feeder-dependent techniques for routine maintenance were first feeder depleted by passaging the cells at 1:3 on Geltrex and cultured on Nutristem. To seed cells onto ECM-immobilized PEG-UV 96-well plates, a single-cell suspension of the hPSC lines was generated as described above. The cells were centrifuged and resuspended at a concentration of 1 × 10^6^ cells/ml in SR medium supplemented with 20 ng/ml bFGF (R&D systems, Minneapolis, Minnesota) and 10 μM ROCK inhibitor Y-27632. Wells were seeded in the PEG-patterned 96-well plates at a density of 60,000 cells/well for plates with colonies of 500 μm diameter, 80,000 cells/well for colonies of 1 mm diameter, and at 120,000 cells/well for plates with colonies of 3 mm diameter and incubated for 2 to 3 h at 37°C. After 2 to 3 h, the medium was changed to SR without ROCKi. When confluent colonies were observed (12–18 h after seeding), the peri-gastrulation–like induction or preneurulation-like induction was initiated as follows. (A) Peri-gastrulation-like induction (Fig 2) was performed in SR medium supplemented with 100 ng/ml of bFGF (R&D) and 50 ng/ml of BMP4. (B) Unless otherwise stated, preneurulation-like induction with 500 μm colonies was performed with SR medium (see ‘Human pluripotent stem cell culture’ section above for composition) supplemented with 100 ng/ml of bFGF with 25 ng/ml of BMP4 and 10 μM SB431542 (‘SB’). (C) Endoderm finger-printing assay (S6 Fig) was performed with N2B27 medium supplemented with 25 ng/ml of Wnt3A and 50 ng/ml of ActivinA. (D) RD-like periodic pattern induction of pSMAD1 activity, and preneurulation-like fates were tested in both SR and N2B27 mediums. In the case of SR, the medium was supplemented with 10 μM SB, 100 ng/ml of bFGF, and either 50 ng/ml or 200 ng/ml of BMP4. In the case of N2B27, the medium was supplemented with 10 μM SB, 10 ng/ml of bFGF, and either 50 ng/ml of 200 ng/ml of BMP4.

### EB differentiation assay

The differentiation media for the EB assay contained 76% DMEM, 20% FBS, 1% Penicillin/Streptomycin, 1% non-essential amino acids, 0.1 mM β-mercaptoethanol, 1% Glutamax (all Invitrogen, Carlsbad, California). A large volume of the medium was prepared with a single batch of FBS and frozen at −80°C and was used to differentiate the EBs made from all the hPSC lines tested. EB formation from the hPSC lines was achieved by generating a single-cell suspension (as described in the section above) directly in the differentiation media supplemented with ROCKi for the first day. The cell suspension was then plated on 24-well microwell plates (Aggrewell, 400 μm, Stem Cell Technologies, Vancouver, British Columbia, Canada). The seeding density was chosen to allow generation of size-controlled EBs (approximately 500 cells/EB) for all hPSC lines. The media was carefully replaced with differentiation media without ROCKi 24 h after seeding to ensure that the EBs were not disturbed. EBs were harvested from the Aggrewell plates each day by adding 1 ml of DMEM into the wells and pipetting till the EBs lifted off from the microwells and frozen as a pellet at −80°C till gene expression was assessed using qPCR.

### CA1 Nog^−/−^ cell line generation

CA1 Noggin knockout lines were generated using a CRISPR/Cas9 mediated donor-free dual knockout using a previously described strategy [72]. The sgRNA design was performed with CRISPRko Azimuth 2.0 (Broad Institute) using human Noggin (NCBI ID9241) as entry data and SpCas9 for the nuclease. The software ranks sgRNAs with high on-target activities and low off-target activities in a combined rank [73]. We chose sgRNA1 (5ʹ-CTGTACGCGTGGAACGACCT-3ʹ) and sgRNA2 (5ʹ–CAAAGGGCTAGAGTTCTCCG-3ʹ) with a combined rank of 4 and 1, respectively. sgRNA1 and 2 can be used individually or applied together to produce Noggin knockout. Latter leads to a deletion of a DNA fragment of 112 bp and to a predetermined stop codon (S8A Fig).

Transfection and evaluation of cutting efficiency: We first evaluated the cutting efficiency of SpCas9 for each individual gRNA on a population level. For this, we seeded CAI hESC into 24-well plates such that they are 50% to 60% confluent at the day of transfection (approximately 24 h after seeding). CmgRNA were generated by mixing 1 μM AltR CRISPR crRNA (IDT, custom oligo entry) with 1 μM AltR CRISPR tacrRNA (IDT, Cat. 1073189), annealed at 95°C for 5 min and cooled down at room temperature. GeneArtTMPlatinumTM Cas9 Nuclease (Invitrogen, B25641) was diluted to 1 μM using OptiMEM (Thermo Fisher Scientific, Waltham, Massachusetts, 31985062). Cas9 and cmgRNA were mixed at a concentration of 0.3 μM each in 25 μl of OptiMEM. After incubation at room temperature for 5 minutes, 1 μl of EditProTM Stem (MTI Globalstem, Waltham, Massachusetts) diluted in 25 μl of OptiMEM was added to the Cas9/cmgRNA complex and incubated for 15 minutes at room temperature. Before adding the reagent, Cas9/cmgRNA mix, to the cells, the medium was replaced with 500 μl/24-well of fresh mTeSR. Medium was replaced 24 h after transfection; 48 h after transfection, cells were harvested by incubation in Gentle Cell Dissociation Reagent (STEMCELL Technologies, 07174) for 7 min. Dissociation reagent was removed and cells were resuspended in cultivation medium, pipetted to single cells, and spin down for 5 min at 200*g*. Cells were resuspended in 25 μl of Cell Lysis Buffer mixed with 1 μl Protein Degrader, both from the GeneArtTM Genomic Cleavage Detection Kit (Invitrogen, A24372). Cells were lysed at 68°C for 15 min, 95°C for 10 min, and kept on ice. PCR was performed using Phusion High Fidelity DNA Polymerase (NEB, M0530) according to manufactures protocol using 2 μl of the cell lysate. Primer for the PCR were the following: (fwd) 5ʹCTACGACCCAGGCTTCATGGCʹ3, (rev) 5ʹGACGGCTTGCACACCATGC3ʹ. PCR product of untransfected and transfected samples were analyzed on 2.5% MetaPhore Agarose Gel (Lonza, Basel, Switzerland) PCR products were analyzed using GeneArt Genomic Cleavage Detection Kit (Invitrogen, Carlsbad, California, A24372) according to manufacturer’s protocol. The cleaved and uncleaved samples were loaded on 2.5% MetaPhore Agarose Gel (Lonza, Basel, Switzerland), and the bands were analyzed using ImageJ. Percentage of gene modification was calculated as described in a previous report (S8B Fig) [74]. Additionally, PCR products were sent for Sanger Sequencing. Chromatograms were analyzed using TIDE [75].

Cell line generation: The cell line was generated using gRNA1 and 2 mixed with Cas9 at 0.3 μM each. The transfection was proceeded with exact same protocol as described above using 6 × 24 wells. After 3 days, cells reached confluency and were seeded to 6-well plates at sufficiently low densities to achieve clonal growth from single cells. Approximately 7 days after seeding, single clones were picked and transferred to 96-well plates; 24 clones were expanded for 2 passages, and PCR was performed on cell lysates as described above (S8C Fig). PCR products were send for Sanger Sequencing and aligned to (NCBI ID9241) and to untransfected WT sequence (S8C Fig). Clones with clear loss of function mutations in both alleles (C1 and C7) were further characterized for their pluripotency marker expression (S8D Fig).

### Quantitative PCR analysis

RNA extraction for all gene expression analysis studies was performed using Qiagen RNAeasy miniprep columns according to the manufacturer’s protocol, and the cDNA was generated using Superscript III reverse transcriptase (Invitrogen, Carlsbad, California) as per the manufacturer’s instructions. The generated cDNA was mixed with primers for the genes of interest and SYBR green mix (Roche, Sigma, St. Louis, Missouri), and the samples were run on an Applied Biosystems QuantStudio 6 flex real-time PCR machine. The relative expression of genes of interest was determined by the delta–delta cycle threshold (ΔΔCt) method with the expression of GAPDH as an internal reference. Primer sequences used are provided in S2 Table.

### Transfection protocol for siRNA transfection

All siRNA transfections were performed with CA1 hPSC line seeded on either PEG plates (500 μm diameter colonies) or 24-well plates for the qPCR control experiments to validate the siRNA specificity. First, the cells seeded in SR medium supplemented with 20 ng/ml bFGF where incubated with transfection reagents for 24 h after which period the medium was changed to the differentiation medium, and the transfection was repeated. Cells were analyzed 24 h after the start of differentiation by qPCR (24 well) for CER1 gene expression or by microscopy (PEG plates) for pSMAD1 spatial trends. The siRNAs used were ON-TARGETplus Human CER1 siRNA SMARTpool and ON-TARGETplus Nontargeting siRNA (Dharmacon, Lafayette, Colorado Cat.No L-012019-00-0005 and D-001810-01-05, respectively). Transfection was performed using siRNAs at the final concentration of 50 nM and Lipofectamine RNAiMAX diluted in OptiMEM medium following manufacturer’s protocol (Thermo Fischer Scientific, Waltham, Massachusetts).

### Immunofluorescent staining, RNAscope multiplex assay, and image analysis

After the peri-gastrulation–like or the preneurulation-like induction was completed, the plates were fixed with 3.7% paraformaldehyde for 20 min, rinsed 3 times with PBS, and then permeabilized with 100% methanol for 3 min. After permeabilization, the patterned colonies were blocked using 10% FBS (Invitrogen, Carlsbad, California) in PBS overnight at 4°C. Primary antibodies were incubated at 4°C overnight (antibody sources and concentrations are shown in S3 Table). The following day, the primary antibodies were removed, and the plates were washed 3 times with PBS followed by incubation with the secondary antibodies and DAPI nuclear antibody at room temperature for 1 h. RNAscope assays were performed as per the detailed instructions provided by the manufacturer (ACD Bio, Newark, California). The positive and negative controls chosen were the standard controls provided by the manufacturer. Single-cell data were acquired by scanning the plates using the Cellomics Arrayscan VTI platform using the ‘TargetActivation.V4’ bioassay algorithm. This algorithm utilizes the expression intensity in the DAPI channel to identify individual nuclei in all fields imaged and acquires the associated intensity of proteins of interest localized within the identified region. As previously described [24], single-cell data extracted from fluorescent images were exported into our custom built software, ContextExplorer [76], which classifies cells into colonies via the DBSCAN algorithm. Cartesian coordinates relative to the colony centroid are computed for every cell within a colony. Hexagonal binning is used to group cells from multiple colonies according to their relative location within a colony. Average protein expression of cells within a bin is represented by the color map, which is normalized to the lowest and highest expressing hexagonal bins. In the line plots of spatial expression trends, cells are grouped in annular bins according to the Euclidean distance between a cell and the colony centroid. For each colony, the mean expression of all cells within an annular bin is computed. The average of all the colony means is displayed in the line plot together with the SD and the 95% confidence interval (CI).

## Supporting information

S1 Fig Characterization of PEG plates. (A–B) C1s spectra acquired using X-Ray Photoelectron Spectroscopy. (A) C1s spectra of glass coverslip incubated with PLL-g-PEG compared to blank glass coverslip. (B) C1s spectra of PLL-g-PEG–coated glass coverslips photo-exposed to Deep-UV light for different times of exposure. Dotted lines signify binding energies associated with untreated glass (285.0 eV), or presence of PEG (286.6 eV). (C) Line plot representation of detected absorbance at 580 nm wavelength of coverslips photo-oxidized for different times of exposure indicating the relative amounts of adsorbed Toluidine Blue-O (assay details in Materials and methods). Data represented as mean (± SD) for 3 technical replicates. The assay was performed once to identify optimal exposure times for our experimental setup. (D–I) Representative images acquired on PEG plates for multiple cell types. (D) MEFs stained for β-actin in green and DAPI in blue. (E) Hemogenic endothelial cells stained for VECAD in green and DAPI in blue. (F) Primary human keratinocytes stained for Keratin 14 in green and Involucrin in red. (G) Mouse embryonic stem cells stained for OCT4 in green. (H) BMP4 treated human induced pluripotent stem cells stained for SOX17 in red. (I) Human endodermal progenitor cells allowed to generate outgrowths stained for NKX6.1 in red and PDX1 in green. Underlying numerical data for this figure can be found in https://osf.io/zrvxj/. BMP4, bone morphogenetic protein 4; C1, carbon 1; MEF, Mouse Embryonic Fibroblasts; NKX6.1, NK6 homeobox 1; OCT4, octamer-binding transcription factor 4; PDX1, pancreatic and duodenal homeobox 1; PEG, Polyethylene Glycol; PLL-g-PEG, Poly-L-Lysine-grafted-Polyethylene Glycol; SOX17, SRY-Box 17; VECAD, vascular endothelial cadherin.
(TIF)

S2 Fig Validation of hPSC patterning in PEG plates. (A) Overview of a previously described micro-patterning based hPSC differentiation assay [30] using OCT4 and SOX2 expression levels as indicators of early fate choices to compare PEG and μCP plates. (B) Quantified compartments of early fate choices as defined in panel A, in both PEG and μCP plates. The media conditions tested were ‘NS’–Nutristem, Apel (vehicle for the following), ‘BMP’ (BMP4), ‘BA’ (BMP4+ActivinA), ‘FSB’ (bFGF+SB431542; see Materials and methods for concentration details). Data represented as mean (+ SD) of 4 independent replicates. The fate choice responses of hPSCs on both the plates were highly correlated (R^2^ > 0.9). (C) Representative immunofluorescent images of hPSC colonies stained for OCT4 and SOX2 in the different media conditions tested. Scale bars indicate 500 μm. (D–E) Comparison of patterning response on PEG plates versus μCP plates. (D) Number of colonies identified per well between PEG and μCP plates. Each dot represents the number of colonies identified per well for 120 randomly chosen wells between the 4 replicates of PEG versus μCP plates. Number of cells identified per colony between PEG and μCP plates. Each dot represents the average number of cells per colony for 120 randomly chosen wells between the 4 replicates of PEG versus μCP plates. (E) Representative images of hPSCs micropatterned in 96-well plates using PEG-based technique versus μCP. Underlying numerical data for this figure can be found in https://osf.io/zrvxj/. hPSC, human pluripotent stem cell; OCT4, octamer-binding transcription factor 4; PEG, Polyethylene Glycol; SOX2, SRY-box 2; μCP, micro-contact printing.
(TIF)

S3 Fig Starting populations of test hPSC lines show high expression of pluripotency associated proteins. (A) FACS plots of OCT4-, SOX2-, and NANOG-expressing cells in the starting populations of H9-1, H9-2, HES2, MEL1, and HES3-1. ‘Secondary-only’ identifies the nonspecific labelling observed due to the secondary antibody. (B) Representative immunofluorescent images from Fig 2 shown with corresponding DAPI staining. FACS, Fluorescence-activated cell sorting; hPSC, human pluripotent stem cell; NANOG, homeobox protein NANOG; OCT4, octamer-binding transcription factor 4; SOX2, SRY-box 2.
(TIF)

S4 Fig Nodal expression dynamics in EB assay of hPSC line panel. Temporal dynamics of Nodal for the test hPSC lines shown for the 3 clusters of Nodal-Strong, Nodal-Intermediate, and Nodal-weak (Fig 3Bii). Each dot represents the detected expression level for a biological replicate. Bar plots represent mean ± SD. Underlying numerical data for this figure can be found in https://osf.io/zrvxj/. EB, embryoid body; hPSC, human pluripotent stem cell.
(TIF)

S5 Fig MIXL1 and EOMES dynamics during EB assay predict endoderm differentiation propensity of hPSC lines. (A) Panel of hPSC lines clustered into 3 groups of ‘Strong’, ‘Medium’, and ‘Weak’ responders for (i) MIXL1 and (ii) EOMES from Fig 3Bii. The expression levels of MIXL1 and EOMES in the pluripotent state (Day 0) shown in the boxes adjacent to the heat maps. (B) Overview of the protocol for directed differentiation towards definitive endoderm. The cells were treated with Wnt3a from 0 h to 24 h and Wnt3a+ActivinA from 24 h to 72 h. (C–D) Efficiency of SOX17 induction in the test hPSCs using the protocol in panel B. (C) Black dash denotes the mean of 3 independent replicates represented by the dots. (D) FACS plots for individual replicates from panel C. (E) FACS plots showing the efficiency of induction of pancreatic progenitors as indicated by the expression of PDX1 and NKX6.1 for candidate hPSC lines from the ‘Strong’ and ‘Weak’ clusters from panel A. The differentiation was performed using a previously described protocol [77]. The data are from 3 independent wells; the experiment was performed twice. The gating was performed using the cells stained with only the secondary antibodies (shown in blue). The samples stained for PDX1 and NKX6.1 are shown in red. Underlying numerical data for this figure can be found in https://osf.io/zrvxj/. EB, embryoid body; EOMES, Eomesodermin; FACS, Fluorescence-activated cell sorting; hPSC, human pluripotent stem cell; MIXL1, Mix Paired-Like Homeobox 1; NKX6.1, NK6 Homeobox 1; PDX1, pancreatic and duodenal homeobox 1; SOX17, SRY-box 17
(TIF)

S6 Fig Peri-gastrulation–like assay predicts endoderm differentiation bias of hPSC lines. (A) Overview of assay for predicting endodermal differentiation bias. Geometrically confined hPSC lines were treated with Wnt+ActivinA for 48 h prior to fixation and staining. (B) Quantified fraction of endodermal cells, defined as double positive for SOX17 and FOXA2, detected within the geometrically confined hPSC colonies. The hPSC lines chosen were one each from the ‘Strong’, ‘Medium’, and ‘Weak’ clusters of MIXL1 and EOMES from S5A Fig. Each data point represents an individual identified colony. Data pooled from 2 different experiments and represented as mean ± SD; *p*-values calculated using one-way ANOVA (Kruskal-Wallis test). (C) Representative immunofluorescent images of colonies from panel B stained for DAPI, SOX17, and FOXA2. Scale bar represents 500 μm. Underlying numerical data for this figure can be found in https://osf.io/zrvxj/. EOMES, Eomesodermin; FOXA2, Forkhead Box A2; hPSC, human pluripotent stem cell; MIXL1, Mix Paired-Like Homeobox 1; SOX17, SRY-box 17
(TIF)

S7 Fig Modulation of Nodal signalling during BMP4 treatment of geometrically-confined hPSC colonies reveals a switch in expression of gastrulation vs pre-neurulation associated genes. Response of modulation of Nodal signalling in expression of gastrulation versus pre-neurulation associated genes in BMP4 treated geometrically-confined CA1 colonies (assay details in Fig 3Di). Individual data points represent biological replicates. Data shown as mean ± SD, and *p*-values calculated using Mann-Whitney U test. Underlying numerical data for this figure can be found in https://osf.io/zrvxj/. BMP4, bone morphogenetic protein 4; hPSC, human pluripotent stem cell.
(TIF)

S8 Fig Generation of CA1 Noggin^−/−^ cell line using CRISPR/Cas9. (A) Schematic of dual knock-out strategy (please see Materials and methods for details) for Noggin (NCBI ID9241). Nucleotide sequence of gRNA binding regions (gRNA 1; green and gRNA2; orange) with expected cutting sites (green nucleotides) and resulting repaired DNA strand. The gRNAs have been selected such that repaired DNA will lead to a predetermined stop codon (TGA). (B) Analysis of gRNA cutting efficiency on transfected hESC CAI population 48 h post-transfection. (Top left) PCR from transfected cell lysate. (Top right) PCR fragments processed with and without T7 Endonuclease (Genomic Cleavage Assay; please see Materials and methods for details). (Bottom) Sanger sequencing of PCR product and subsequent decomposition and analysis using TIDE online analyzing tool. PCR from WT CA1 was used as control. Bar chart shows frequency, type, and position of mutations. (C) A total of 24 clones were analyzed by PCR (top left) and by Sanger Sequencing (top right). Sequencing revealed 2 clones (C1 and C7) with mutations in both alleles (bottom). The clone C1 had homozygous mutations in both alleles (deletions in gRNA1 region and an insertion in gRNA2 region), and C7 had one allele (Allele1) with the whole fragment between gRNA1 and gRNA2 deleted and the other allele (Allele2) with a 14 bp deletion in the gRNA2 region. The 2 bands (2 alleles) of C7 were separately purified from Agarose gel before sequencing. (D) Pluripotency marker staining of C1 and C7 CA1 Noggin^−/−^ lines of CA1s using a conjugated PE-mouse anti-Oct3/4 antibody and a conjugated V450-mouse anti-human SSEA4 antibody with corresponding fluorophore labelled isotype controls (please see Materials and methods). Underlying numerical data for this figure can be found in https://osf.io/zrvxj/. gRNA, guide RNA; hESC, human embryonic stem cell; PCR, Polymerase chain reaction; PE, Phycoerythrin; SSEA4, Stage-specific embryonic antigen-4; TGA, nucleic acid sequence; TIDE, Tracking of Indels by DEcomposition; WT, wild type.
(TIF)

S9 Fig pSMAD1 gradient formation during inhibition of Nodal is consistent with a BMP4-Noggin RD network mediated organization. (Ai–ii) Line plot representation of radial gradient formed in colonies of 500 μm diameter treated with varying doses of BMP4 (3.125 ng/ml, 6.25 ng/ml, 12.5 ng/ml, and 25 ng/ml) in induction medium containing SB. (i) The gradients shown individually. Data pooled from two experiments and represent 299, 293, 302, and 343 colonies for the respective doses. SDs shown in grey and 95% confidence intervals shown in black. (ii) Line plots shown in one graph for comparison of pSMAD1 levels at colony periphery. (B) Average pSMAD1 expression levels shown as overlay of the detected colonies (numbers mentioned in panel A). (C) Representative immunofluorescent images of pSMAD1 for respective conditions. Scale bars represent 200 μm. (D) Average pSMAD1 expression of 987, 528, 280, 182, 107, and 89 colonies for varying colony sizes (200 μm, 300 μm 400 μm, 500 μm, 600 μm, and 700 μm) treated with 25 ng/ml of BMP4 in induction medium containing SB. Data pooled from 2 experiments. SDs shown in grey and 95% confidence intervals shown in black. (E) Representative immunofluorescent images of pSMAD1 for respective conditions. Scale bars represent 200 μm. Underlying numerical data for this figure can be found in https://osf.io/zrvxj/. BMP4, bone morphogenetic protein 4; pSMAD1, phosphorylated SMAD1; RD, reaction-diffusion; SB, SB431542 (Nodal signalling inhibitor)
(TIF)

S10 Fig Nodal signalling contributes to the formation of the pSMAD1 gradient. (A–B) Representative images of 500 m colonies stained for DAPI and pSMAD1 for the experimental results showed in Fig 5A–5C. (A) Representative images for the condition when the hPSC colonies were treated with 25 ng/ml of BMP4 in the presence of Nodal. White arrows mark regions that show a second peak of pSMAD1 activity consistent with the RD paradigm. (B) Representative images for the condition when the hPSC colonies were treated with 25 ng/ml of BMP4 in the presence of SB. White arrowheads show pSMAD1 staining far into the colony from the periphery. (C) Representative immunofluorescence images of 500 μm diameter hPSC colonies stained for pSMAD1 after 24 h of BMP4 (50 ng/ml) treatment. Scalebar represents 200 μm. (D) Average pSMAD1 intensity represented as overlays of 368 and 411 colonies when treated with 50 ng/ml of BMP4 in the presence of either Nodal or SB, respectively. Data pooled from 2 experiments. (E) The average radial trends of pSMAD1 shown as line plots. SDs shown in black. The *p*-values were calculated using Mann-Whitney U-test. *p < 0.0001. (F–I) Gene expression changes in BMP4 treated hPSCs when culture medium was supplemented with either Nodal or SB. The changes in expression are shown for Nodal (F), LEFTY-A (G), GDF3 (H), and FST (I). Data in panels F through I represent 3 biological replicates and are shown as normalized (with respect to the expression observed in Nodal supplemented media) fold change relative to the Day 0 hPSC population for each biological replicate. The *p*-values were calculated using two-sided paired *t* test. Underlying numerical data for this figure can be found in https://osf.io/zrvxj/. BMP4, bone morphogenetic protein 4; FST, Follistatin; GDF3, growth differentiation factor 3; hPSC, human pluripotent stem cell; LEFTY-A, Left-right determining factor A; pSMAD1, phosphorylated SMAD1; RD, reaction-diffusion; SB, SB431542 (Nodal signalling inhibitor)
(TIF)

S11 Fig Validation of signalling logic underlying the fate switch in the peri-gastrulation–associated BRA, and preneurulation-associated GATA3 expression. (A–C) In the condition when Nodal signalling is permitted, after 24 h of BMP4 treatment of 500 μm diameter rudimentary expression of BRA is observed. (A) Line plot representation of spatial profiles of pSMAD1, SMAD2, and BRA expression. SDs shown in grey, 95% confidence intervals shown in black. (B) Average expression of pSMAD1, SMAD2, and BRA from data shown in panel A represented as a 2D intensity map. The data for panels and B were from 349 colonies. The experiment was performed twice. (C) Representative immunofluorescent images for a colony stained for DAPI, pSMAD1, pSMAD2, and BRA. (D–F) In the condition when Nodal signalling is inhibited through SB supplementation, after 24 h of BMP4 treatment of 500 μm diameter expression of GATA3 is observed. (D) Line plot representation of spatial profiles of pSMAD1, pSMAD2, and GATA3 expression. SDs shown in grey, 95% confidence intervals shown in black. (E) Average expression of pSMAD1, pSMAD2, and GATA3 from data shown in panel D represented as a 2D intensity map. The data for panels D and E were from 319 colonies. The experiment was performed twice. (F) Representative immunofluorescent images for a colony stained for DAPI, pSMAD1, pSMAD2, and GATA3. Underlying numerical data for this figure can be found in https://osf.io/zrvxj/. BMP4, bone morphogenetic protein 4; BRA, Brachyury; GATA3, GATA-binding protein 3; pSMAD1, phosphorylated SMAD1
(TIF)

S12 Fig Nodal signalling characterisation and BRA expression in 500 μm colonies treated with 25 ng/ml BMP4. (A–B) pSMAD2 expression profile in 500 μm diameter colonies after 24 h of BMP4 treatment (25 ng/ml) shows a peak similar to the peak observed in SMAD2 expression profile (S11A Fig). Average expression profile extracted from 49 colonies is shown as overlays (A) and line plot (B). Data pooled from 2 experiments. SDs shown in grey and 95% confidence intervals shown in black. Percentage of cells expressing BRA in 500 μm colonies when the colonies were induced to differentiate at 25 ng/ml of BMP4 for either 24 h or 36 h in the presence of Nodal. Each data point represents an identified colony. Data pooled from 2 experiments. The number of colonies were 388 for 24 h treatment and 456 for 36 h treatment with BMP4. Bars represent mean ± SD. The *p*-value was calculated using Kruskal-Wallis test. (B) Average expression of BRA at 36 h after BMP4 treatment shown as (i) line plots and (ii) 2D intensity map. The standard deviations in panel B(i) are shown in grey and the 95% confidence intervals are shown in black. (C) Representative immunofluorescent images for a colony stained for DAPI and BRA. Underlying numerical data for this figure can be found in https://osf.io/zrvxj/. BMP4, bone morphogenetic protein 4; BRA, Brachyury; pSMAD2, phosphorylated SMAD1; SMAD2, SMAD family member 2.
(TIF)

S13 Fig Computational model predicts Nodal dependent changes in organized pSMAD1 signalling gradient. (A–B) Predictions from the computational model (please see S1 Text for details) in terms of the response of perturbing Nodal signalling along with the doses of BMP4. The modelled predictions are consistent with the experimentally observed results (Fig 5A–5C, S10A–S10C Fig). (A) The predicted organization of BMP signalling activity with or without Nodal signalling at a dose of 25 ng/ml of BMP4. (B) The predicted organization of BMP signalling activity with or without Nodal signalling at a dose of 50 ng/ml of BMP4. Underlying numerical data for this figure can be found in https://osf.io/zrvxj/. BMP4, bone morphogenetic protein 4; pSMAD1, phosphorylated SMAD1
(TIF)

S14 Fig In situ staining for expression profile of Nodal and BMP antagonists downstream of Nodal signalling. (A) Spatial profile of the mean of Nodal (in green) and CER1 (in yellow) expression detected in 184 colonies for ‘Nodal’ (in solid lines) and 191 colonies for ‘SB’ (in dashed lines). The experiment was performed once. The variance between the colonies shown in black. (B) Representative immunofluorescent images of Nodal, and BMP antagonists downstream of Nodal signalling shown for the conditions when the colonies were treated with BMP4 either in the presence of Nodal ligands (‘Nodal’) or in the presence of SB (‘SB’). The representative images for the positive and negative control images for all channels imaged are also shown. The control probes were POLR2A (green), PPIB (yellow), and UBC (red); the negative controls target bacterial DapB gene in all 3 channels. Underlying numerical data for this figure can be found in https://osf.io/zrvxj/. BMP, bone morphogenetic protein; CER1, Cerberus; DapB, RNA Polymerase II Subunit A; POLR2A, RNA Polymerase II Subunit A; PPIB, Peptidylprolyl Isomerase B; SB, SB431542; UBC, Ubiquitin C.
(TIF)

S15 Fig Nodal inhibition during BMP4 treatment of hPSC colonies abrogates peri-gastrulation–associated fates and induces preneurulation-associated fates. (Ai–ii) Overview of experimental setup. (i) Geometrically confined hPSC colonies were treated with BMP4 for 48 h. (ii) Media tested. ‘Vehicle’ indicated SR medium (see Materials and methods for composition) supplemented with BMP4 and bFGF. ‘SB’ indicated vehicle supplemented with 10 μM SB431542. (Bi–ii) Response of gastrulation-associated fate patterning in Vehicle and SB conditions. (i) Representative immunofluorescent images for BRA, GATA6, EOMES, and SOX17 for Vehicle and SB conditions. White triangle represents nonspecific background staining for EOMES. Scale bar represents 200 μm. (ii) Quantified expression of gastrulation-associated fates. Each data point represents an identified colony. The total number of colonies were (373, 248), (325, 317), (81, 72), and (506, 329) for BRA, GATA6, EOMES, and SOX17, respectively, for (Vehicle and SB treatments). The data are pooled from 2 experiments except for EOMES, which was performed once. (Ci–ii) Preneurulation-like fate patterning observed in the presence of SB. (i) Representative immunofluorescent images of TFAP2A, SIX1, OTX2, and co-stained image of SOX2 and GATA3. Scale bar represents 200 μm. (ii) Average radial expression intensity of the preneurulation-associated fates represented as line plots. SD shown in grey, and 95% confidence intervals shown in black. Underlying numerical data for this figure can be found in https://osf.io/zrvxj/. bFGF,; BMP4, bone morphogenetic protein 4; BRA, Brachyury; EOMES, Eomesodermin; GATA6, GATA binding protein 6; hPSC, human pluripotent stem cell; OTX2, Orthodenticle Homeobox 2; SB, SB431542; SIX1, SIX Homeobox 1; SOX17, SRY-box 17; SR, serum replacement; TFAP2A, transcription factor AP2-alpha.
(TIF)

S16 Fig SOX2 and GATA3 expression is consistent with a pSMAD1 dose-dependent fate patterning. (A) Overview of experimental setup. Perturbing BMP4 dose in induction medium while maintaining colony size varies pSMAD1 concentration levels at the colony periphery (see S9A–S9C Fig for details). (B) Percentage of cells in each identified colony expressing GATA3 when colonies of 500 μm in diameter were treated with varying doses of BMP4 (3.125 ng/ml, 6.25 ng/ml, 12.5 ng/ml, and 25 ng/ml) in induction medium. Each data point represents an identified colony. The total number of colonies were 131, 208, 215, and 244 for the respective doses. Data pooled from 2 experiments. Bars represent mean ± SD. The *p*-value was calculated using Kruskal-Wallis test. (C) Representative immunofluorescent images of colonies stained for SOX2 and GATA3. Scale bar represents 200 μm. (D) Overview of experimental setup. Perturbing the colony size while maintaining the BMP4 dose constant in the induction medium varies the pSMAD1 levels at the colony center (see S9D Fig and S9E Fig for details). (E) Percentage of cells in each identified colony expressing SOX2 when colonies of varying sizes (200 μm, 300 μm, 400 μm, 500 μm, 600 μm, and 700 μm in diameter) were treated with 25 ng/ml of BMP4 in induction medium. Each data point represents an identified colony. The total number of colonies were 932, 439, 256, 175, 122, and 45 for the respective sizes. Data pooled from 2 experiments. Bars represent mean ± SD. The *p*-value was calculated using Kruskal-Wallis test. (F) Representative immunofluorescent images of colonies stained for SOX2 and GATA3. Scale bar represents 200 μm. Underlying numerical data for this figure can be found in https://osf.io/zrvxj/. BMP4, bone morphogenetic protein 4; GATA3, GATA binding protein 3; pSMAD1, phosphorylated SMAD1; SOX2, SRY box 2.
(TIF)

S17 Fig GATA3 expression arises as a function of BMP4 dose and induction time. Percentage of cells expressing SOX2 and GATA in 500 μm colonies induced to differentiate at varying concentrations of BMP4 (3.125 ng/ml, 6.25 ng/ml, 12.5 ng/ml, and 25 ng/ml) and induction times (12 h, 24 h, 36 h, and 48 h). Each data point represents an identified colony, and each condition had over 100 colonies. Data pooled from 2 experiments. Bars represent mean ± SD. Underlying numerical data for this figure can be found in https://osf.io/zrvxj/. BMP4, bone morphogenetic protein 4
(TIF)

S18 Fig Changing shapes does not affect outside-in spatial patterning. Representative images of various shapes of geometrically-confined hPSC colonies treated with BMP4 and SB in SR medium. Varying colony shapes does not result in any deviation from anticipated fate patterning. The experiment was performed once. Scale bar represents 200 μm. BMP4, bone morphogenetic protein 4; hPSC, human pluripotent stem cell; SR, serum replacement.
(TIF)

S19 Fig No pSMAD1 staining observed when large geometrically confined hPSC colonies are treated with 50 ng/ml BMP4 and SB in SR medium. (A–B) No discernable pSMAD1 expression detected with geometrically confined hPSC colonies of 3 mm diameter were treated with 50 ng/ml of BMP4 and SB for 24 h in SR medium. (A) Stitched images of the entire colony stained for pSMAD1 shown in greyscale for ease of visibility. (B) Enlarged fields that are indicated by white squares in panel A. White arrows indicate regions that contain cells with positive pSMAD1 expression. Scale bar represents 1 mm. BMP4, bone morphogenetic protein 4; hPSC, human pluripotent stem cell; pSMAD1, phosphorylated SMAD1; SR, serum replacement.
(TIF)

S20 Fig Inconclusive staining of pSMAD1 observed when large geometrically confined hPSC colonies are treated with 200 ng/ml BMP4 and SB in SR medium. (A–B) Inconclusive staining of pSMAD1 expression detected with geometrically confined hPSC colonies of 3 mm diameter were treated with 200 ng/ml of BMP4 and SB for 24 h in SR medium. (A) Stitched images of the entire colony stained for pSMAD1 shown in greyscale for ease of visibility. (B) Enlarged fields that are indicated by white squares in panel A. White arrows at the colony periphery indicate regions that contain cells with positive pSMAD1 expression. White arrows with accompanying question marks indicate regions that possibly show expression of pSMAD1; however, the staining in these regions is inconclusive. Scale bar represents 1 mm. BMP4, bone morphogenetic protein 4; hPSC, human pluripotent stem cell; pSMAD1, phosphorylated SMAD1; SB, SB431542; SR, serum replacement.
(TIF)

S21 Fig RD-like foci of pSMAD1 detected when large geometrically confined hPSC colonies are treated with 50 ng/ml BMP4 and SB in N2B27 medium. RD-like foci of pSMAD1 expression detected with geometrically confined hPSC colonies of 3 mm diameter were treated with 50 ng/ml of BMP4 and SB for 24 h in N2B27 medium. Representative stitched images of the entire colony stained for pSMAD1 shown in greyscale for ease of visibility along with DAPI staining. Enlarged fields that are indicated by white squares in stitched images. White arrows indicate regions that contain cells with positive pSMAD1 expression. Scale bar represents 1 mm. BMP4, bone morphogenetic protein 4; hPSC, human pluripotent stem cell; pSMAD1, phosphorylated SMAD1; RD, reaction-diffusion; SB, SB431542.
(TIF)

S22 Fig Treatment of large geometrically confined hPSC colonies with 200 ng/ml BMP4 and SB in N2B27 medium results in prominent expression of RD-like foci of pSMAD1 activity. RD-like foci of pSMAD1 expression detected with geometrically confined hPSC colonies of 3 mm diameter were treated with 50 ng/ml of BMP4 and SB for 24 h in N2B27 medium. Representative stitched images of the entire colony stained for pSMAD1 shown in greyscale for ease of visibility along with DAPI staining. Enlarged fields that are indicated by white squares in stitched images. White arrows indicate regions that contain cells with positive pSMAD1 expression. Scale bar represents 1 mm. BMP4, bone morphogenetic protein 4; hPSC, human pluripotent stem cell; pSMAD1, phosphorylated SMAD1; RD, reaction-diffusion; SB, SB431542.
(TIF)

S23 Fig No foci of early NN fates detected when large geometrically confined hPSC colonies are treated with 200 ng/ml BMP4 and SB in SR medium. Treatment of geometrically confined hPSC colonies with 200 ng/ml of BMP4 and SB for 48 h does not display obvious RD-like foci of GATA3 expression. Representative stitched images of 3 mm diameter hPSC colonies differentiated in SR medium supplemented with 200 ng/ml of BMP4 for 48 h. Scale bar represents 1 mm. Zoomed section outlined by the white square shown below stitched colonies. The experiment was repeated 2 times. BMP4, bone morphogenetic protein 4; GATA3, GATA binding protein 3; hPSC, human pluripotent stem cell; NN, non-neural; RD, reaction-diffusion; SB, SB431542; SR, serum replacement.
(TIF)

S24 Fig Minor foci of early NN fates detected when large geometrically confined hPSC colonies are treated with 50 ng/ml BMP4 and SB in N2B27 medium. Treatment of geometrically confined hPSC colonies with 50 ng/ml of BMP4 and SB for 48 h results in RD-like foci of GATA3 expression. Representative stitched images of 3 mm diameter hPSC colonies differentiated with 50 ng/ml of BMP4 for 48 h. Scale bar represents 1 mm. Zoomed section outlined by the white square shown below stitched images. While arrows indicate GATA3 positive and SOX2 negative regions. The experiment was repeated 2 times. BMP4, bone morphogenetic protein 4; GATA3, GATA binding protein 3; hPSC, human pluripotent stem cell; NN, non-neural; RD, reaction-diffusion; SB, SB431542; SOX2, SRY box 2.
(TIF)

S25 Fig RD-like foci of early NN fates detected when large geometrically confined hPSC colonies are treated with 200 ng/ml BMP4 and SB in N2B27 medium. Representative immunofluorescent images of geometrically confined hPSC colonies of 3 mm diameter stained for SOX2 and GATA3. The colonies were treated with 200 ng/ml of BMP4 and SB for 48 h. Scale bar represents 1 mm. BMP4, bone morphogenetic protein 4; GATA3, GATA binding protein 3; hPSC, human pluripotent stem cell; NN, non-neural; RD, reaction-diffusion; SB, SB431542; SOX2, SRY box 2.
(TIF)

S26 Fig Representative images with associated DAPI stains for colonies shown in Fig 7B–7D. (A) Representative image of colony treated with BMP4 and SB for 24 h and NOG + SB for 72 h (see Fig 7A) stained for DAPI and PAX6. (B) Representative image of colony treated with BMP4 and SB for 24 h and CHIR + SB for 48 h (see Fig 7A) stained for DAPI and SOX10. (C) Representative image of colony treated with BMP4 and SB for 72 h (see Fig 7A) stained for DAPI, TROMA1, and DLX5. BMP4, bone morphogenetic protein 4; CHIR, CHIR99021; DLX5, Distal-Less Homeobox 5; NOG, Noggin; PAX6, Paired Box 6; SB, SB431542; SOX10, SRY-box 10; TROMA1, Cytokeratin-8 antibody clone.
(TIF)

S27 Fig GATA3 expression surpasses CDX2 expression in colonies treated with BMP4 and SB. (A) Overview of experimental setup. Geometrically confined colonies of 200 μm diameter were treated with BMP4 and SB at concentrations of 12.5 ng/ml and 25 ng/ml for 24 h and stained for CDX2 and GATA3. (B–C) Quantified expression of CDX2 and GATA3 detected in the geometrically confined colonies treated with BMP4 and SB reveals that GATA3 expression surpasses that of CDX2. (B) A total of 220 colonies treated with 12.5 ng/ml of BMP4. (C) A total of 141 colonies treated with 25 ng/ml of BMP4. (D–E) Representative immunofluorescent images of colonies (from B and C) stained for DAPI, GATA3, and CDX2. Scale bar represents 500 μm. Underlying numerical data for this figure can be found in https://osf.io/zrvxj/. BMP4, bone morphogenetic protein 4; CDX2, Caudal Type Homeobox 2; GATA3, GATA binding protein 3; SB, SB431542.
(TIF)

S28 Fig Preneurulation-like platform can give rise to bona fide NPB markers. (A) Overview of experimental setup. Geometrically confined hPSC colonies were treated with SB and 25 ng/ml of BMP4 for 24 h and then were differentiated with an adapted protocol that has been reported to generate NPB identities (Xue and colleagues, 2018). The differentiation protocol consisted of treating with SR medium supplemented with SB for 3 days with a transient 24 h pulse of CHIR (3 μM). At D4, the colonies were fixed and stained for ZIC1, MSX1, and SOX10. (B) Average expression of ZIC1, MSX1, and SOX10 shown as line plots in red, green, and yellow, respectively. The SDs are shown in black, and the 95% confidence intervals are shown in grey. The number of colonies included in the overlays were 119 for ZIC1, 181 for MSX1, and 116 for SOX10. (C) Representative immunofluorescent images for ZIC1, MSX1, and SOX10. Scale bar represents 500 μm. Underlying numerical data for this figure can be found in https://osf.io/zrvxj/. BMP4, bone morphogenetic protein 4; CHIR, CHIR99021; hPSC, human pluripotent stem cell; MSX1, Msh homeobox 1; NPB, NP border; SOX10, SRY box 10; SR, serum replacement; ZIC1, Zic Family Member 1.
(TIF)

S1 Table hPSC lines utilized in this study. Complete list of hPSC lines used for the study and the culture conditions employed for their maintenance. The Wnt reporter line was generated using a puromycin selection cassette. We did not perform any selection, and, consequently, the Wnt activity reporter was undetectable in any of our experiments. hPSC, human pluripotent stem cell.
(XLSX)

S2 Table List of primers used in this study. (XLSX)

S3 Table List of antibodies used in this study. (XLSX)

S1 Text Description of the mathematical modelling and image analysis. (DOCX)

## Abbreviations

BMP4: bone morphogenetic protein 4
BMPi: bone morphogenetic protein inhibitors
BRA: Brachyury (also known as T)
C1: carbon 1
CER1: Cerberus
CHIR: CHIR99021
DLX5: distal-less homeobox 5
DUV: Deep UV
EB: embryoid body
ECM: extracellular matrix
EDC: 1-Ethyl-3-(3-dimethylaminopropyl)carbodiimide
EOMES: Eomesodermin
GDF3: growth differentiation factor 3
FOXA2: forkhead box protein A2
FST: Follistatin
GATA3: GATA binding protein 3
HAND1: Heart- and neural crest derivatives-expressed protein 1
hiPSC: human-induced pluripotent stem cell
hPSC: human pluripotent stem cell
KDR: Kinase insert domain receptor
KSR: knockout Serum Replacement
LPA: lysophosphatidic acid
MEF: Mouse Embryonic Fibroblasts
mEpiSC: mouse epiblast stem cell
MESP1: mesoderm posterior bHLH transcription factor 1
MIXL1: Mix paired-like homeobox protein 1
MSX1: Msh Homeobox 1
MYB: Myb proto-oncogene protein
NANOG: Homeobox protein NANOG
NC: neural crest
NHS: N-HydroxySuccinimide
NKX6.1: NK6 homeobox 1
NN: non-neural
NNE: NN ectoderm
NP: neural plate
NPB: NP border
OTX2: Orthodenticle homeobox 2
PAX3: paired box gene 3
PDX1: pancreatic and duodenal homeobox 1
EG: Polyethylene Glycol
PI: positional information
PLL-g-PEG: Poly-L-Lysine-grafted-Polyethylene Glycol
PN: preneural
POU5F1: POU domain, class 5, transcription factor 1 (also known as OCT4)
pSMAD1: phosphorylated SMAD1
RD: reaction-diffusion
RUNX1: Runt-related transcription factor 1
SB: SB431542
siRNA: small interfering RNA
SIX1: Sineoculis homeobox homolog 1
SIX3: Sineoculis homeobox homolog 3
SOX1: SRY-box transcription factor 1
SOX10: SRY-box transcription factor 10
SOX17: SRY-box transcription factor 17
SOX9: SRY-box transcription factor 9
SR: serum replacement
T: T box transcription factor T (also known as Brachyury)
TAL1: T-cell acute lymphocytic leukemia protein 1
TBO: Toluidine blue-O
TFAP2A: transcription factor AP2-alpha
TGFβ: transforming growth factor beta
TROMA1: cytokeratin-8 antibody clone
VECAD: vascular endothelial cadherin
XPS: X-Ray Photoelectron Spectroscopy
ZIC1: Zic Family Member 1
μCP: micro-contact printing
