# Supplementary figures and images for: High-throughput micropatterning platform reveals Nodal-dependent bisection of peri-gastrulation–associated versus preneurulation-associated fate patterning
Source: PLoS Biol. 2019 Oct 21;17(10):e3000081. doi: 10.1371/journal.pbio.3000081 (PMC6822778; doi:10.1371/journal.pbio.3000081)

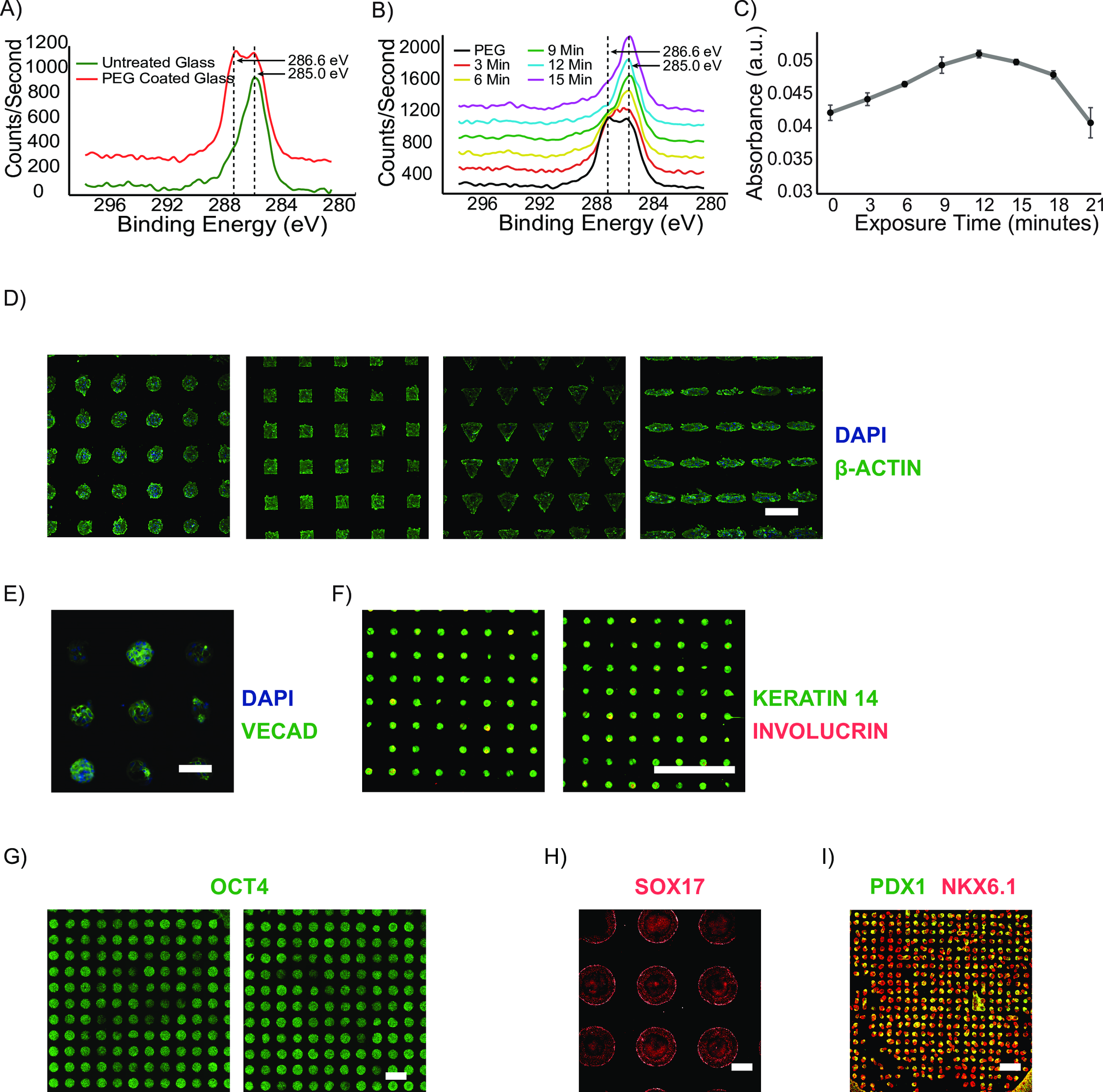

Supplement: S1 Fig — (A–B) C1s spectra acquired using X-Ray Photoelectron Spectroscopy. (A) C1s spectra of glass coverslip incubated with PLL-g-PEG compared to blank glass coverslip. (B) C1s spectra of PLL-g-PEG–coated glass coverslips photo-exposed to Deep-UV light for different times of exposure. Dotted lines signify binding energies associated with untreated glass (285.0 eV), or presence of PEG (286.6 eV). (C) Line plot representation of detected absorbance at 580 nm wavelength of coverslips photo-oxidized for different times of exposure indicating the relative amounts of adsorbed Toluidine Blue-O (assay details in Materials and methods). Data represented as mean (± SD) for 3 technical replicates. The assay was performed once to identify optimal exposure times for our experimental setup. (D–I) Representative images acquired on PEG plates for multiple cell types. (D) MEFs stained for β-actin in green and DAPI in blue. (E) Hemogenic endothelial cells stained for VECAD in green and DAPI in blue. (F) Primary human keratinocytes stained for Keratin 14 in green and Involucrin in red. (G) Mouse embryonic stem cells stained for OCT4 in green. (H) BMP4 treated human induced pluripotent stem cells stained for SOX17 in red. (I) Human endodermal progenitor cells allowed to generate outgrowths stained for NKX6.1 in red and PDX1 in green. Underlying numerical data for this figure can be found in https://osf.io/zrvxj/. BMP4, bone morphogenetic protein 4; C1, carbon 1; MEF, Mouse Embryonic Fibroblasts; NKX6.1, NK6 homeobox 1; OCT4, octamer-binding transcription factor 4; PDX1, pancreatic and duodenal homeobox 1; PEG, Polyethylene Glycol; PLL-g-PEG, Poly-L-Lysine-grafted-Polyethylene Glycol; SOX17, SRY-Box 17; VECAD, vascular endothelial cadherin. (TIF) [file pbio.3000081.s001.tif]

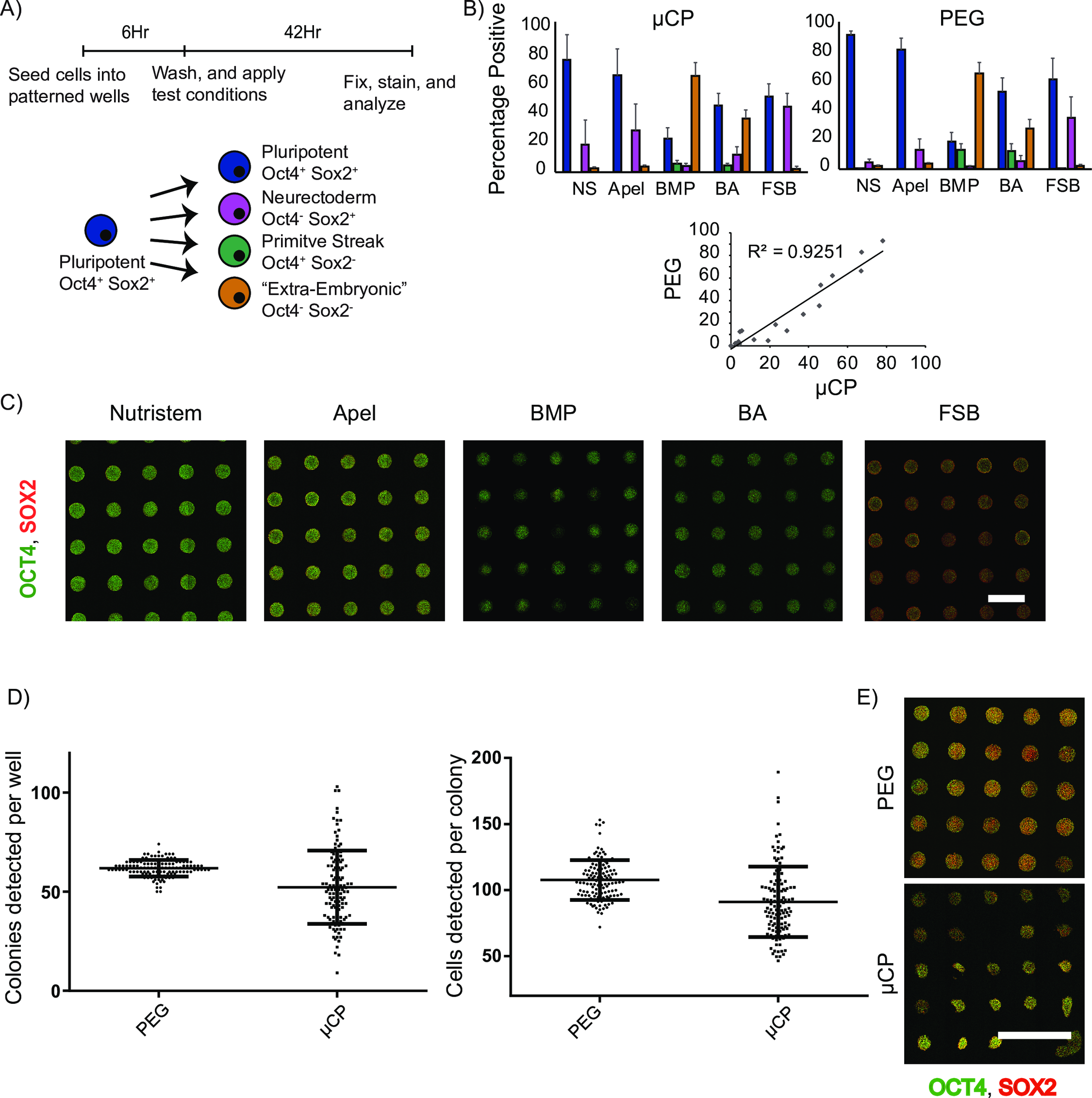

Supplement: S2 Fig — (A) Overview of a previously described micro-patterning based hPSC differentiation assay [30] using OCT4 and SOX2 expression levels as indicators of early fate choices to compare PEG and μCP plates. (B) Quantified compartments of early fate choices as defined in panel A, in both PEG and μCP plates. The media conditions tested were ‘NS’–Nutristem, Apel (vehicle for the following), ‘BMP’ (BMP4), ‘BA’ (BMP4+ActivinA), ‘FSB’ (bFGF+SB431542; see Materials and methods for concentration details). Data represented as mean (+ SD) of 4 independent replicates. The fate choice responses of hPSCs on both the plates were highly correlated (R2 > 0.9). (C) Representative immunofluorescent images of hPSC colonies stained for OCT4 and SOX2 in the different media conditions tested. Scale bars indicate 500 μm. (D–E) Comparison of patterning response on PEG plates versus μCP plates. (D) Number of colonies identified per well between PEG and μCP plates. Each dot represents the number of colonies identified per well for 120 randomly chosen wells between the 4 replicates of PEG versus μCP plates. Number of cells identified per colony between PEG and μCP plates. Each dot represents the average number of cells per colony for 120 randomly chosen wells between the 4 replicates of PEG versus μCP plates. (E) Representative images of hPSCs micropatterned in 96-well plates using PEG-based technique versus μCP. Underlying numerical data for this figure can be found in https://osf.io/zrvxj/. hPSC, human pluripotent stem cell; OCT4, octamer-binding transcription factor 4; PEG, Polyethylene Glycol; SOX2, SRY-box 2; μCP, micro-contact printing. (TIF) [file pbio.3000081.s002.tif]

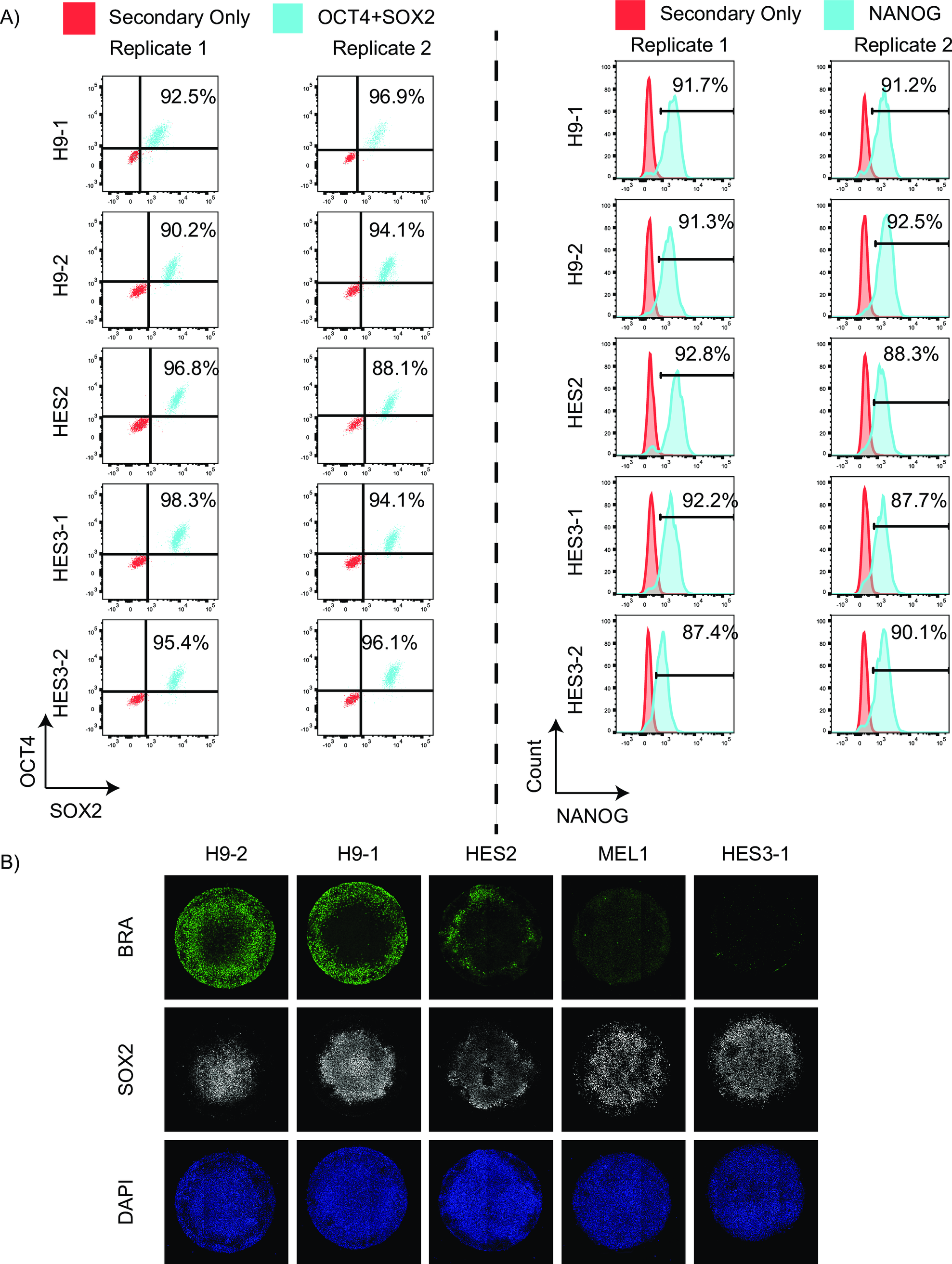

Supplement: S3 Fig — (A) FACS plots of OCT4-, SOX2-, and NANOG-expressing cells in the starting populations of H9-1, H9-2, HES2, MEL1, and HES3-1. ‘Secondary-only’ identifies the nonspecific labelling observed due to the secondary antibody. (B) Representative immunofluorescent images from Fig 2 shown with corresponding DAPI staining. FACS, Fluorescence-activated cell sorting; hPSC, human pluripotent stem cell; NANOG, homeobox protein NANOG; OCT4, octamer-binding transcription factor 4; SOX2, SRY-box 2. (TIF) [file pbio.3000081.s003.tif]

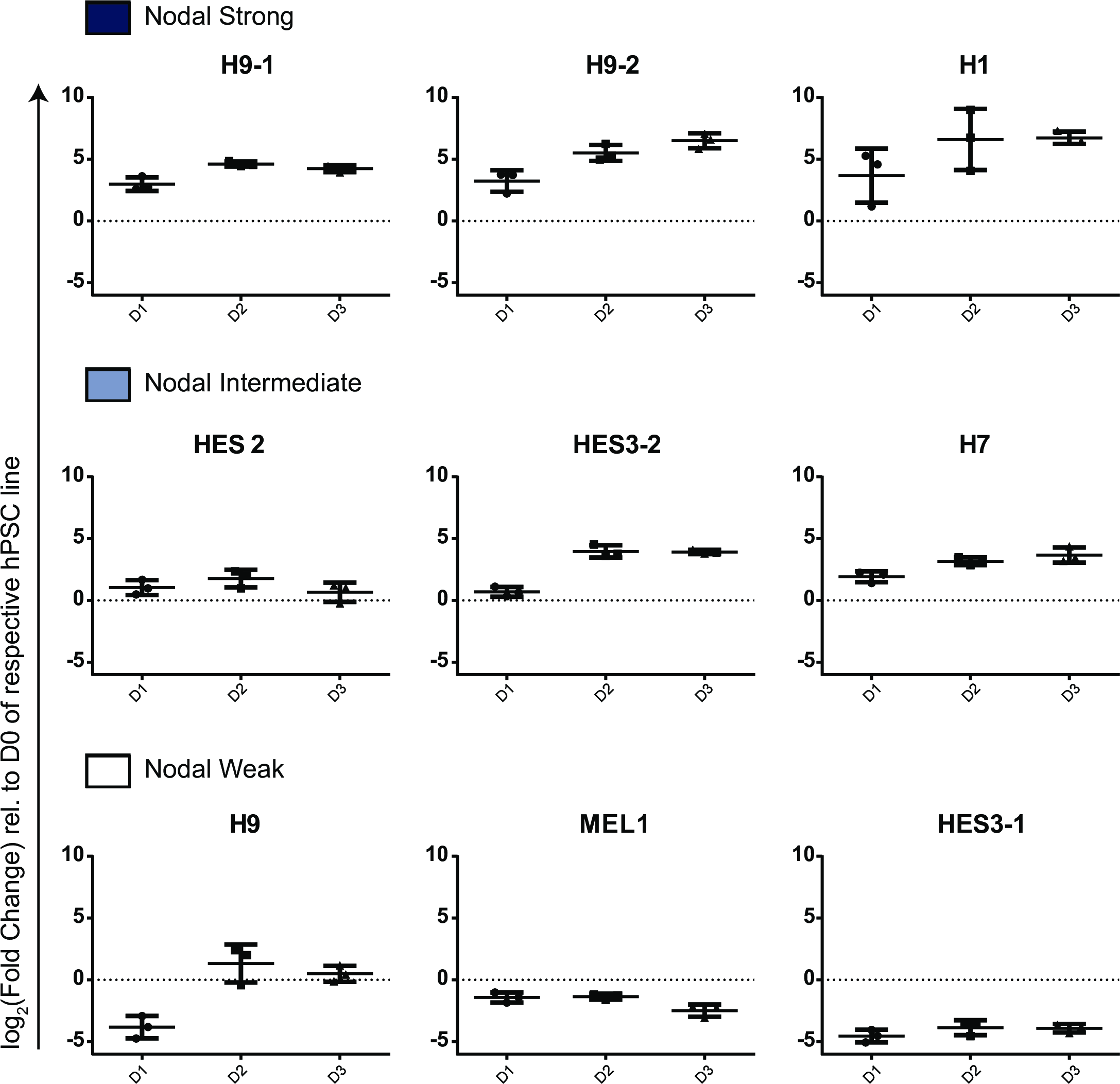

Supplement: S4 Fig — Temporal dynamics of Nodal for the test hPSC lines shown for the 3 clusters of Nodal-Strong, Nodal-Intermediate, and Nodal-weak (Fig 3Bii). Each dot represents the detected expression level for a biological replicate. Bar plots represent mean ± SD. Underlying numerical data for this figure can be found in https://osf.io/zrvxj/. EB, embryoid body; hPSC, human pluripotent stem cell. (TIF) [file pbio.3000081.s004.tif]

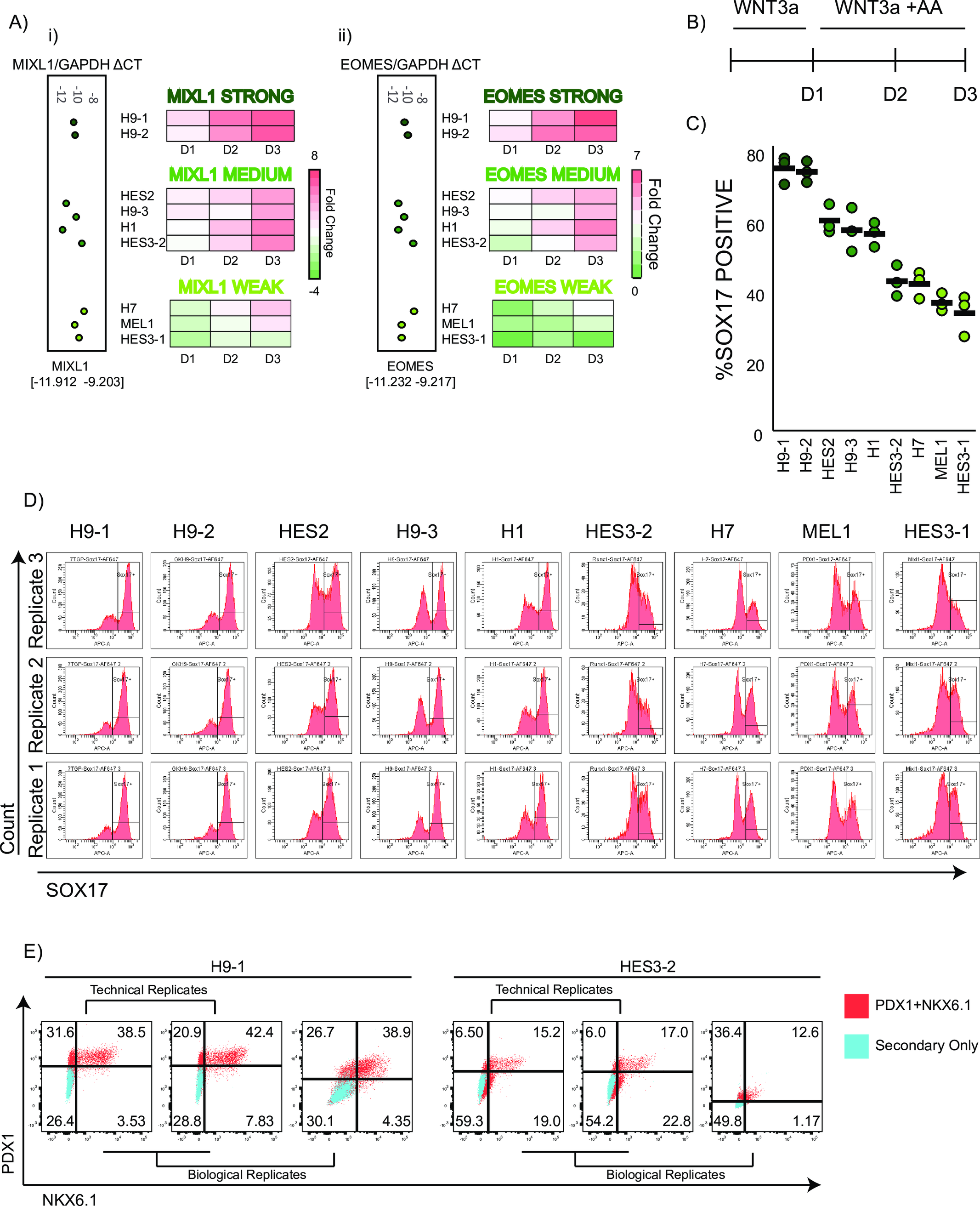

Supplement: S5 Fig — (A) Panel of hPSC lines clustered into 3 groups of ‘Strong’, ‘Medium’, and ‘Weak’ responders for (i) MIXL1 and (ii) EOMES from Fig 3Bii. The expression levels of MIXL1 and EOMES in the pluripotent state (Day 0) shown in the boxes adjacent to the heat maps. (B) Overview of the protocol for directed differentiation towards definitive endoderm. The cells were treated with Wnt3a from 0 h to 24 h and Wnt3a+ActivinA from 24 h to 72 h. (C–D) Efficiency of SOX17 induction in the test hPSCs using the protocol in panel B. (C) Black dash denotes the mean of 3 independent replicates represented by the dots. (D) FACS plots for individual replicates from panel C. (E) FACS plots showing the efficiency of induction of pancreatic progenitors as indicated by the expression of PDX1 and NKX6.1 for candidate hPSC lines from the ‘Strong’ and ‘Weak’ clusters from panel A. The differentiation was performed using a previously described protocol [77]. The data are from 3 independent wells; the experiment was performed twice. The gating was performed using the cells stained with only the secondary antibodies (shown in blue). The samples stained for PDX1 and NKX6.1 are shown in red. Underlying numerical data for this figure can be found in https://osf.io/zrvxj/. EB, embryoid body; EOMES, Eomesodermin; FACS, Fluorescence-activated cell sorting; hPSC, human pluripotent stem cell; MIXL1, Mix Paired-Like Homeobox 1; NKX6.1, NK6 Homeobox 1; PDX1, pancreatic and duodenal homeobox 1; SOX17, SRY-box 17 (TIF) [file pbio.3000081.s005.tif]

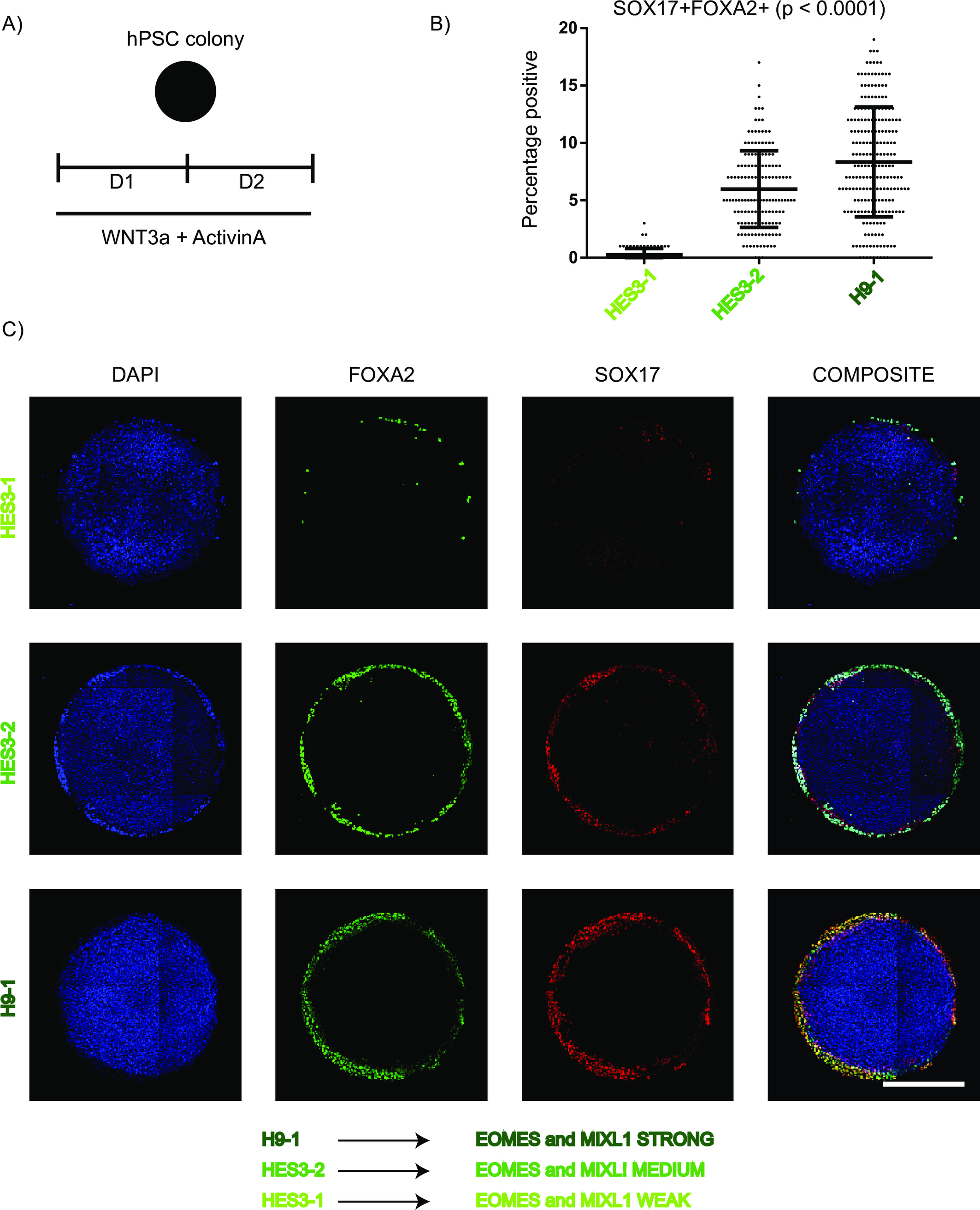

Supplement: S6 Fig — (A) Overview of assay for predicting endodermal differentiation bias. Geometrically confined hPSC lines were treated with Wnt+ActivinA for 48 h prior to fixation and staining. (B) Quantified fraction of endodermal cells, defined as double positive for SOX17 and FOXA2, detected within the geometrically confined hPSC colonies. The hPSC lines chosen were one each from the ‘Strong’, ‘Medium’, and ‘Weak’ clusters of MIXL1 and EOMES from S5A Fig. Each data point represents an individual identified colony. Data pooled from 2 different experiments and represented as mean ± SD; p-values calculated using one-way ANOVA (Kruskal-Wallis test). (C) Representative immunofluorescent images of colonies from panel B stained for DAPI, SOX17, and FOXA2. Scale bar represents 500 μm. Underlying numerical data for this figure can be found in https://osf.io/zrvxj/. EOMES, Eomesodermin; FOXA2, Forkhead Box A2; hPSC, human pluripotent stem cell; MIXL1, Mix Paired-Like Homeobox 1; SOX17, SRY-box 17 (TIF) [file pbio.3000081.s006.tif]

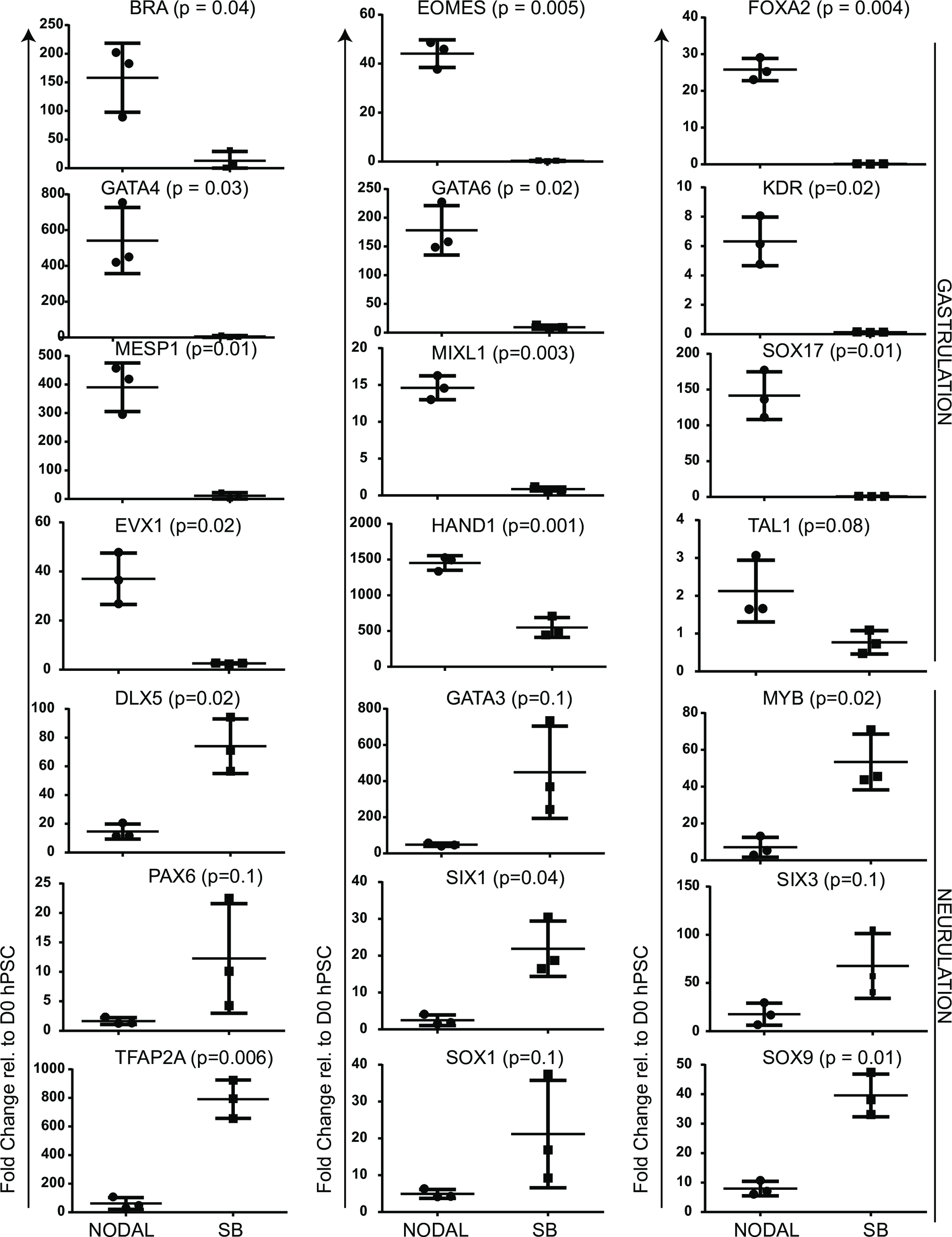

Supplement: S7 Fig — Response of modulation of Nodal signalling in expression of gastrulation versus pre-neurulation associated genes in BMP4 treated geometrically-confined CA1 colonies (assay details in Fig 3Di). Individual data points represent biological replicates. Data shown as mean ± SD, and p-values calculated using Mann-Whitney U test. Underlying numerical data for this figure can be found in https://osf.io/zrvxj/. BMP4, bone morphogenetic protein 4; hPSC, human pluripotent stem cell. (TIF) [file pbio.3000081.s007.tif]

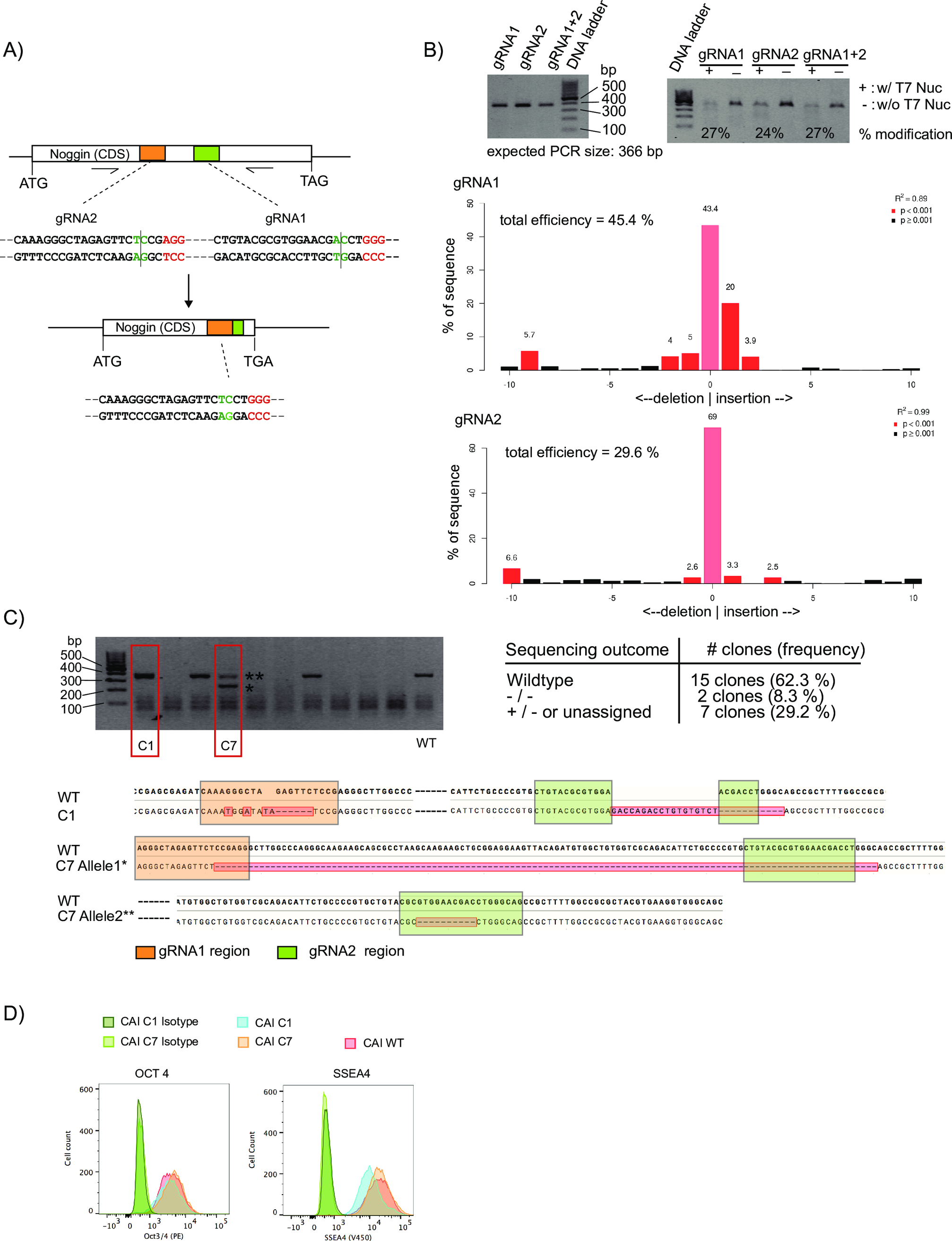

Supplement: S8 Fig — (A) Schematic of dual knock-out strategy (please see Materials and methods for details) for Noggin (NCBI ID9241). Nucleotide sequence of gRNA binding regions (gRNA 1; green and gRNA2; orange) with expected cutting sites (green nucleotides) and resulting repaired DNA strand. The gRNAs have been selected such that repaired DNA will lead to a predetermined stop codon (TGA). (B) Analysis of gRNA cutting efficiency on transfected hESC CAI population 48 h post-transfection. (Top left) PCR from transfected cell lysate. (Top right) PCR fragments processed with and without T7 Endonuclease (Genomic Cleavage Assay; please see Materials and methods for details). (Bottom) Sanger sequencing of PCR product and subsequent decomposition and analysis using TIDE online analyzing tool. PCR from WT CA1 was used as control. Bar chart shows frequency, type, and position of mutations. (C) A total of 24 clones were analyzed by PCR (top left) and by Sanger Sequencing (top right). Sequencing revealed 2 clones (C1 and C7) with mutations in both alleles (bottom). The clone C1 had homozygous mutations in both alleles (deletions in gRNA1 region and an insertion in gRNA2 region), and C7 had one allele (Allele1) with the whole fragment between gRNA1 and gRNA2 deleted and the other allele (Allele2) with a 14 bp deletion in the gRNA2 region. The 2 bands (2 alleles) of C7 were separately purified from Agarose gel before sequencing. (D) Pluripotency marker staining of C1 and C7 CA1 Noggin−/− lines of CA1s using a conjugated PE-mouse anti-Oct3/4 antibody and a conjugated V450-mouse anti-human SSEA4 antibody with corresponding fluorophore labelled isotype controls (please see Materials and methods). Underlying numerical data for this figure can be found in https://osf.io/zrvxj/. gRNA, guide RNA; hESC, human embryonic stem cell; PCR, Polymerase chain reaction; PE, Phycoerythrin; SSEA4, Stage-specific embryonic antigen-4; TGA, nucleic acid sequence; TIDE, Tracking of Indels by DEcomposition; WT, wild type. [file pbio.3000081.s008.tif]

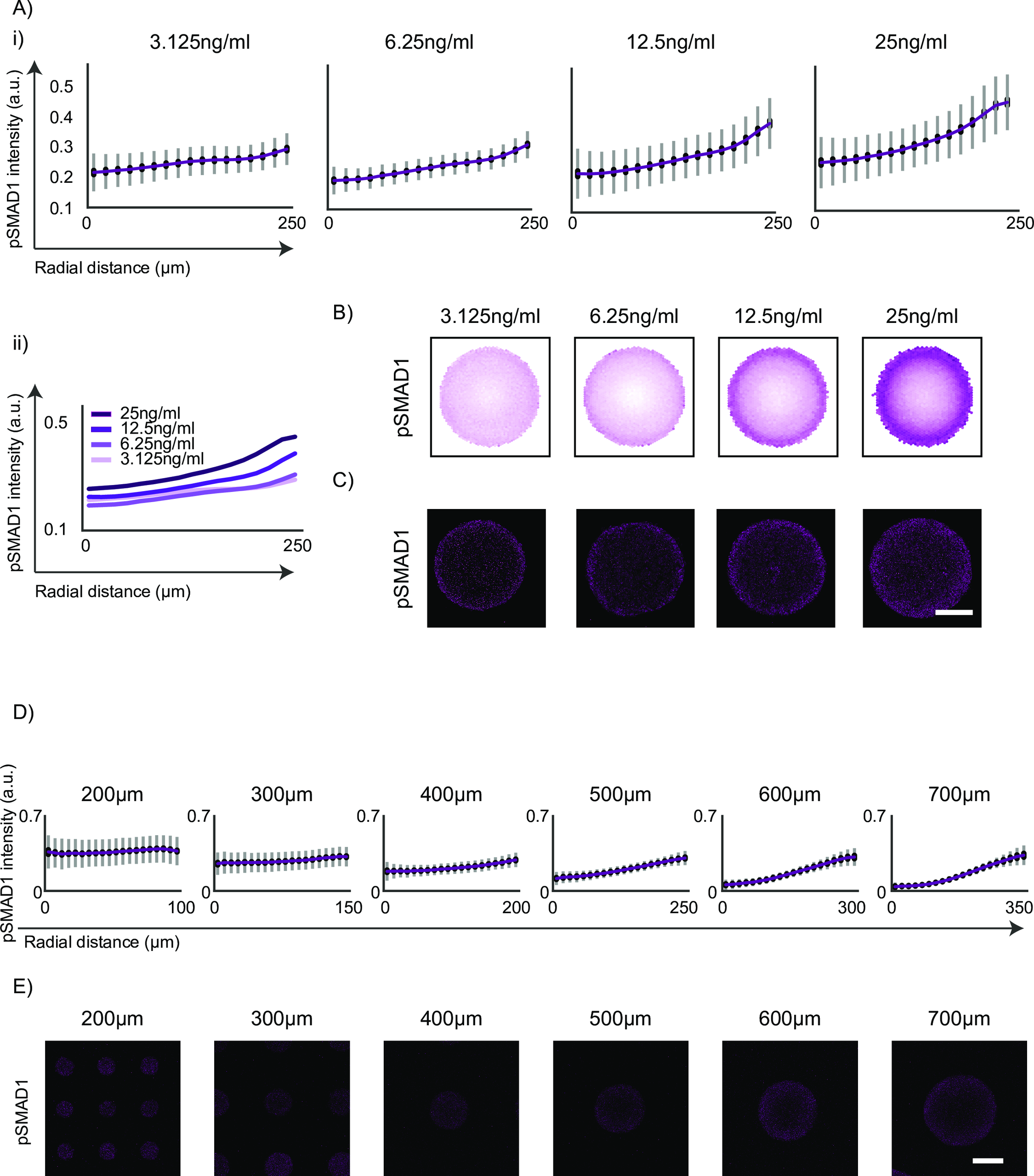

Supplement: S9 Fig — (Ai–ii) Line plot representation of radial gradient formed in colonies of 500 μm diameter treated with varying doses of BMP4 (3.125 ng/ml, 6.25 ng/ml, 12.5 ng/ml, and 25 ng/ml) in induction medium containing SB. (i) The gradients shown individually. Data pooled from two experiments and represent 299, 293, 302, and 343 colonies for the respective doses. SDs shown in grey and 95% confidence intervals shown in black. (ii) Line plots shown in one graph for comparison of pSMAD1 levels at colony periphery. (B) Average pSMAD1 expression levels shown as overlay of the detected colonies (numbers mentioned in panel A). (C) Representative immunofluorescent images of pSMAD1 for respective conditions. Scale bars represent 200 μm. (D) Average pSMAD1 expression of 987, 528, 280, 182, 107, and 89 colonies for varying colony sizes (200 μm, 300 μm 400 μm, 500 μm, 600 μm, and 700 μm) treated with 25 ng/ml of BMP4 in induction medium containing SB. Data pooled from 2 experiments. SDs shown in grey and 95% confidence intervals shown in black. (E) Representative immunofluorescent images of pSMAD1 for respective conditions. Scale bars represent 200 μm. Underlying numerical data for this figure can be found in https://osf.io/zrvxj/. BMP4, bone morphogenetic protein 4; pSMAD1, phosphorylated SMAD1; RD, reaction-diffusion; SB, SB431542 (Nodal signalling inhibitor) (TIF) [file pbio.3000081.s009.tif]

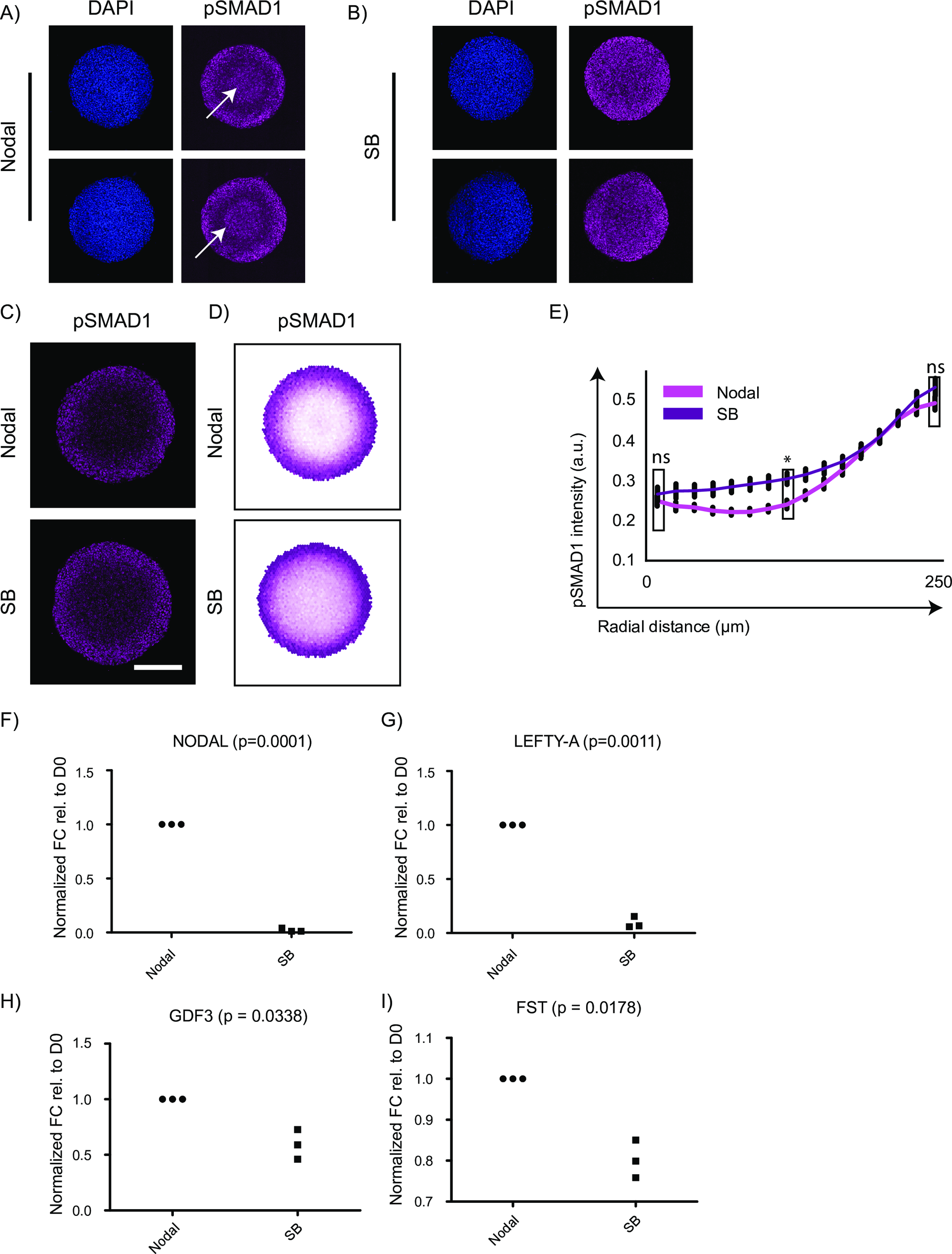

Supplement: S10 Fig — (A–B) Representative images of 500 m colonies stained for DAPI and pSMAD1 for the experimental results showed in Fig 5A–5C. (A) Representative images for the condition when the hPSC colonies were treated with 25 ng/ml of BMP4 in the presence of Nodal. White arrows mark regions that show a second peak of pSMAD1 activity consistent with the RD paradigm. (B) Representative images for the condition when the hPSC colonies were treated with 25 ng/ml of BMP4 in the presence of SB. White arrowheads show pSMAD1 staining far into the colony from the periphery. (C) Representative immunofluorescence images of 500 μm diameter hPSC colonies stained for pSMAD1 after 24 h of BMP4 (50 ng/ml) treatment. Scalebar represents 200 μm. (D) Average pSMAD1 intensity represented as overlays of 368 and 411 colonies when treated with 50 ng/ml of BMP4 in the presence of either Nodal or SB, respectively. Data pooled from 2 experiments. (E) The average radial trends of pSMAD1 shown as line plots. SDs shown in black. The p-values were calculated using Mann-Whitney U-test. *p < 0.0001. (F–I) Gene expression changes in BMP4 treated hPSCs when culture medium was supplemented with either Nodal or SB. The changes in expression are shown for Nodal (F), LEFTY-A (G), GDF3 (H), and FST (I). Data in panels F through I represent 3 biological replicates and are shown as normalized (with respect to the expression observed in Nodal supplemented media) fold change relative to the Day 0 hPSC population for each biological replicate. The p-values were calculated using two-sided paired t test. Underlying numerical data for this figure can be found in https://osf.io/zrvxj/. BMP4, bone morphogenetic protein 4; FST, Follistatin; GDF3, growth differentiation factor 3; hPSC, human pluripotent stem cell; LEFTY-A, Left-right determining factor A; pSMAD1, phosphorylated SMAD1; RD, reaction-diffusion; SB, SB431542 (Nodal signalling inhibitor) (TIF) [file pbio.3000081.s010.tif]

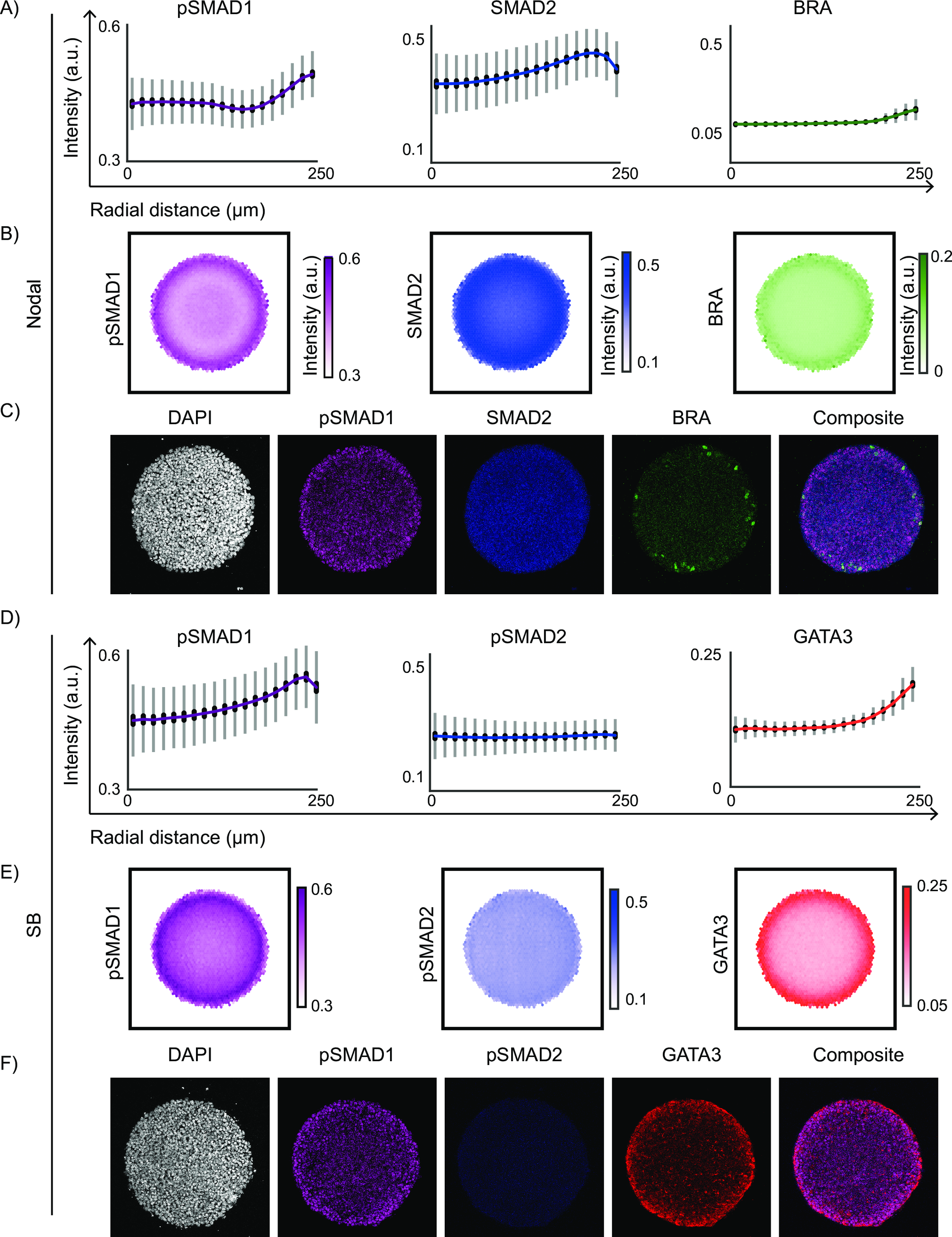

Supplement: S11 Fig — (A–C) In the condition when Nodal signalling is permitted, after 24 h of BMP4 treatment of 500 μm diameter rudimentary expression of BRA is observed. (A) Line plot representation of spatial profiles of pSMAD1, SMAD2, and BRA expression. SDs shown in grey, 95% confidence intervals shown in black. (B) Average expression of pSMAD1, SMAD2, and BRA from data shown in panel A represented as a 2D intensity map. The data for panels and B were from 349 colonies. The experiment was performed twice. (C) Representative immunofluorescent images for a colony stained for DAPI, pSMAD1, pSMAD2, and BRA. (D–F) In the condition when Nodal signalling is inhibited through SB supplementation, after 24 h of BMP4 treatment of 500 μm diameter expression of GATA3 is observed. (D) Line plot representation of spatial profiles of pSMAD1, pSMAD2, and GATA3 expression. SDs shown in grey, 95% confidence intervals shown in black. (E) Average expression of pSMAD1, pSMAD2, and GATA3 from data shown in panel D represented as a 2D intensity map. The data for panels D and E were from 319 colonies. The experiment was performed twice. (F) Representative immunofluorescent images for a colony stained for DAPI, pSMAD1, pSMAD2, and GATA3. Underlying numerical data for this figure can be found in https://osf.io/zrvxj/. BMP4, bone morphogenetic protein 4; BRA, Brachyury; GATA3, GATA-binding protein 3; pSMAD1, phosphorylated SMAD1 (TIF) [file pbio.3000081.s011.tif]

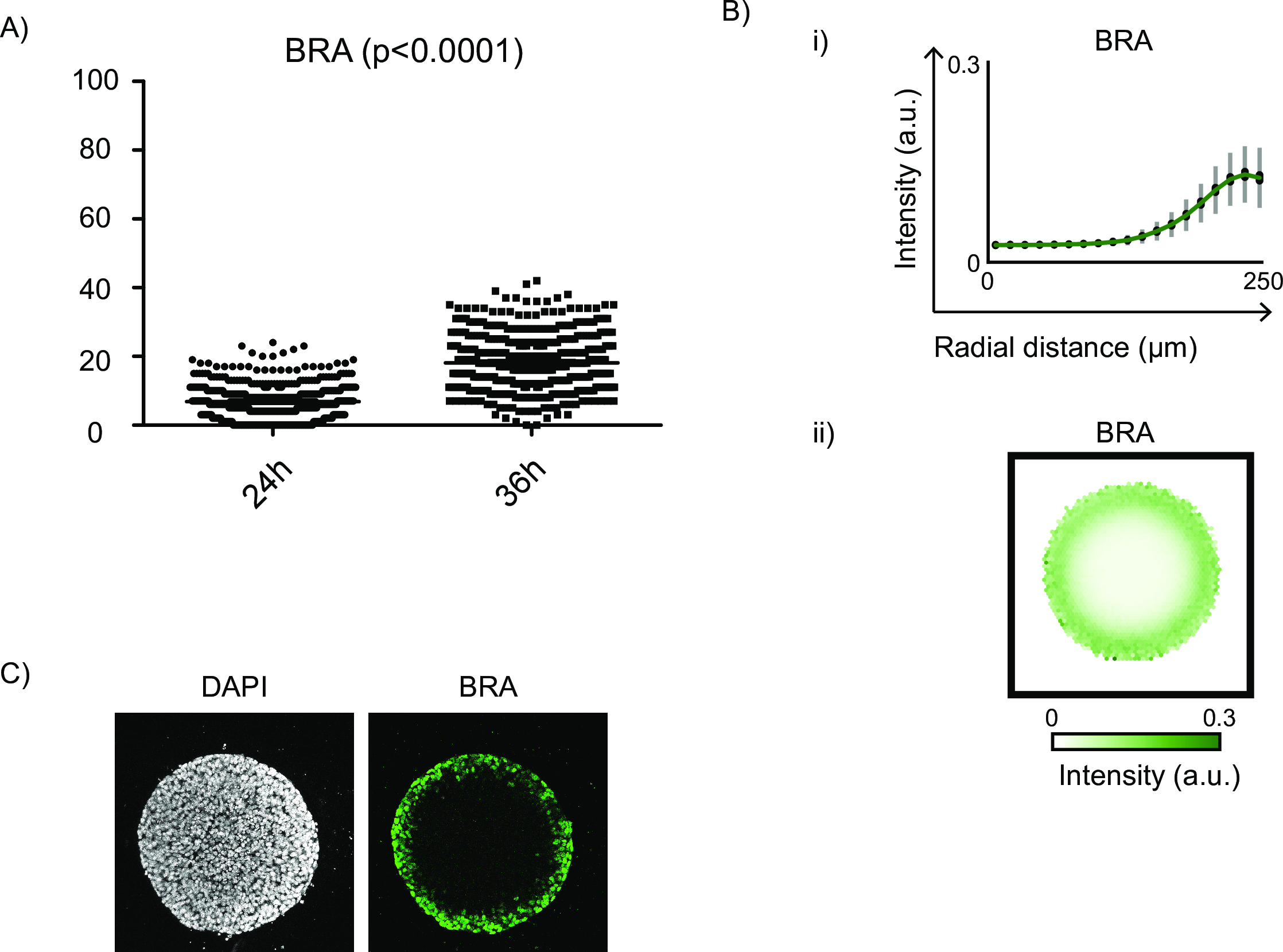

Supplement: S12 Fig — (A–B) pSMAD2 expression profile in 500 μm diameter colonies after 24 h of BMP4 treatment (25 ng/ml) shows a peak similar to the peak observed in SMAD2 expression profile (S11A Fig). Average expression profile extracted from 49 colonies is shown as overlays (A) and line plot (B). Data pooled from 2 experiments. SDs shown in grey and 95% confidence intervals shown in black. Percentage of cells expressing BRA in 500 μm colonies when the colonies were induced to differentiate at 25 ng/ml of BMP4 for either 24 h or 36 h in the presence of Nodal. Each data point represents an identified colony. Data pooled from 2 experiments. The number of colonies were 388 for 24 h treatment and 456 for 36 h treatment with BMP4. Bars represent mean ± SD. The p-value was calculated using Kruskal-Wallis test. (B) Average expression of BRA at 36 h after BMP4 treatment shown as (i) line plots and (ii) 2D intensity map. The standard deviations in panel B(i) are shown in grey and the 95% confidence intervals are shown in black. (C) Representative immunofluorescent images for a colony stained for DAPI and BRA. Underlying numerical data for this figure can be found in https://osf.io/zrvxj/. BMP4, bone morphogenetic protein 4; BRA, Brachyury; pSMAD2, phosphorylated SMAD1; SMAD2, SMAD family member 2. (TIF) [file pbio.3000081.s012.tif]

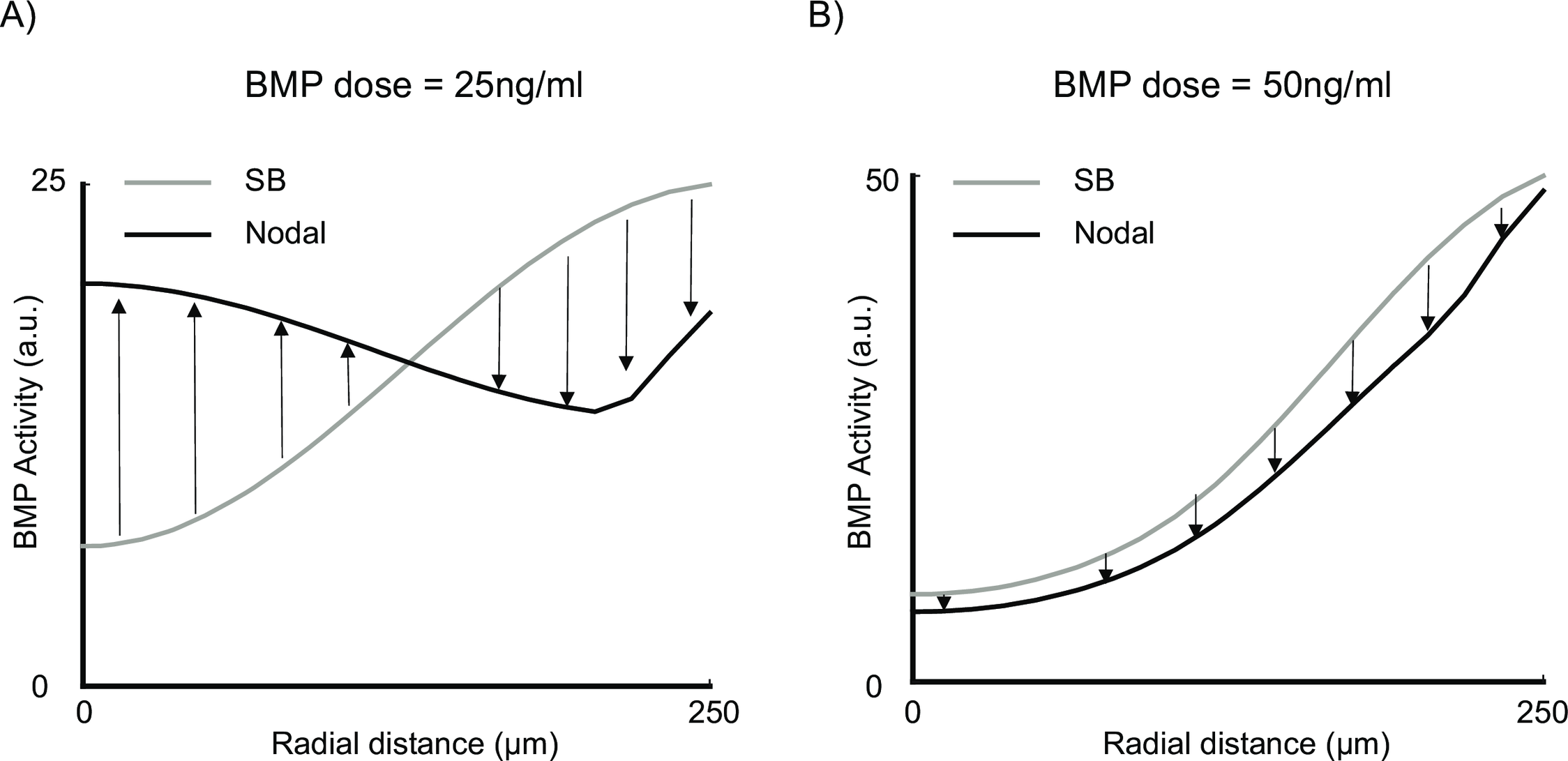

Supplement: S13 Fig — (A–B) Predictions from the computational model (please see S1 Text for details) in terms of the response of perturbing Nodal signalling along with the doses of BMP4. The modelled predictions are consistent with the experimentally observed results (Fig 5A–5C, S10A–S10C Fig). (A) The predicted organization of BMP signalling activity with or without Nodal signalling at a dose of 25 ng/ml of BMP4. (B) The predicted organization of BMP signalling activity with or without Nodal signalling at a dose of 50 ng/ml of BMP4. Underlying numerical data for this figure can be found in https://osf.io/zrvxj/. BMP4, bone morphogenetic protein 4; pSMAD1, phosphorylated SMAD1 (TIF) [file pbio.3000081.s013.tif]

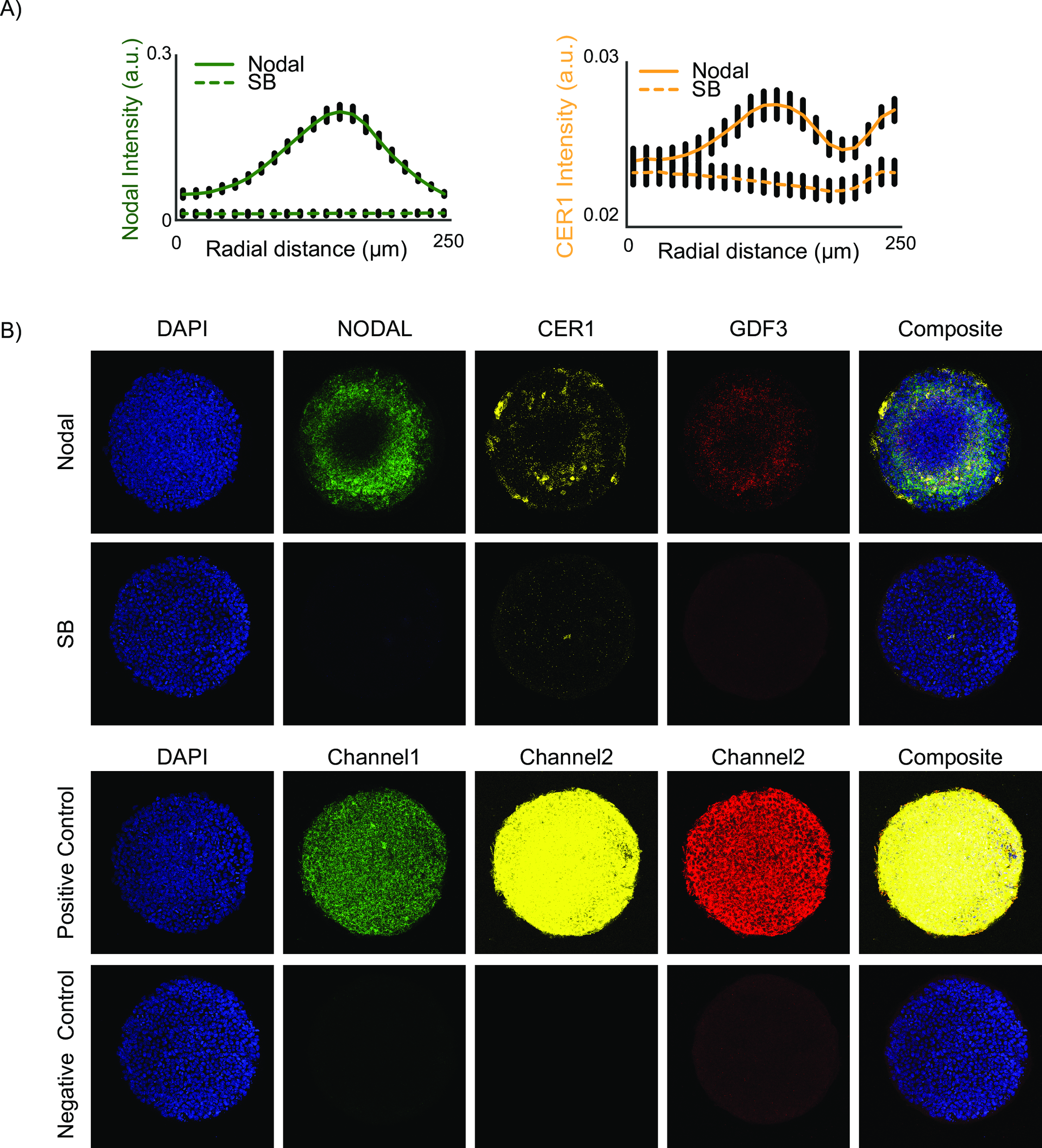

Supplement: S14 Fig — (A) Spatial profile of the mean of Nodal (in green) and CER1 (in yellow) expression detected in 184 colonies for ‘Nodal’ (in solid lines) and 191 colonies for ‘SB’ (in dashed lines). The experiment was performed once. The variance between the colonies shown in black. (B) Representative immunofluorescent images of Nodal, and BMP antagonists downstream of Nodal signalling shown for the conditions when the colonies were treated with BMP4 either in the presence of Nodal ligands (‘Nodal’) or in the presence of SB (‘SB’). The representative images for the positive and negative control images for all channels imaged are also shown. The control probes were POLR2A (green), PPIB (yellow), and UBC (red); the negative controls target bacterial DapB gene in all 3 channels. Underlying numerical data for this figure can be found in https://osf.io/zrvxj/. BMP, bone morphogenetic protein; CER1, Cerberus; DapB, RNA Polymerase II Subunit A; POLR2A, RNA Polymerase II Subunit A; PPIB, Peptidylprolyl Isomerase B; SB, SB431542; UBC, Ubiquitin C. (TIF) [file pbio.3000081.s014.tif]

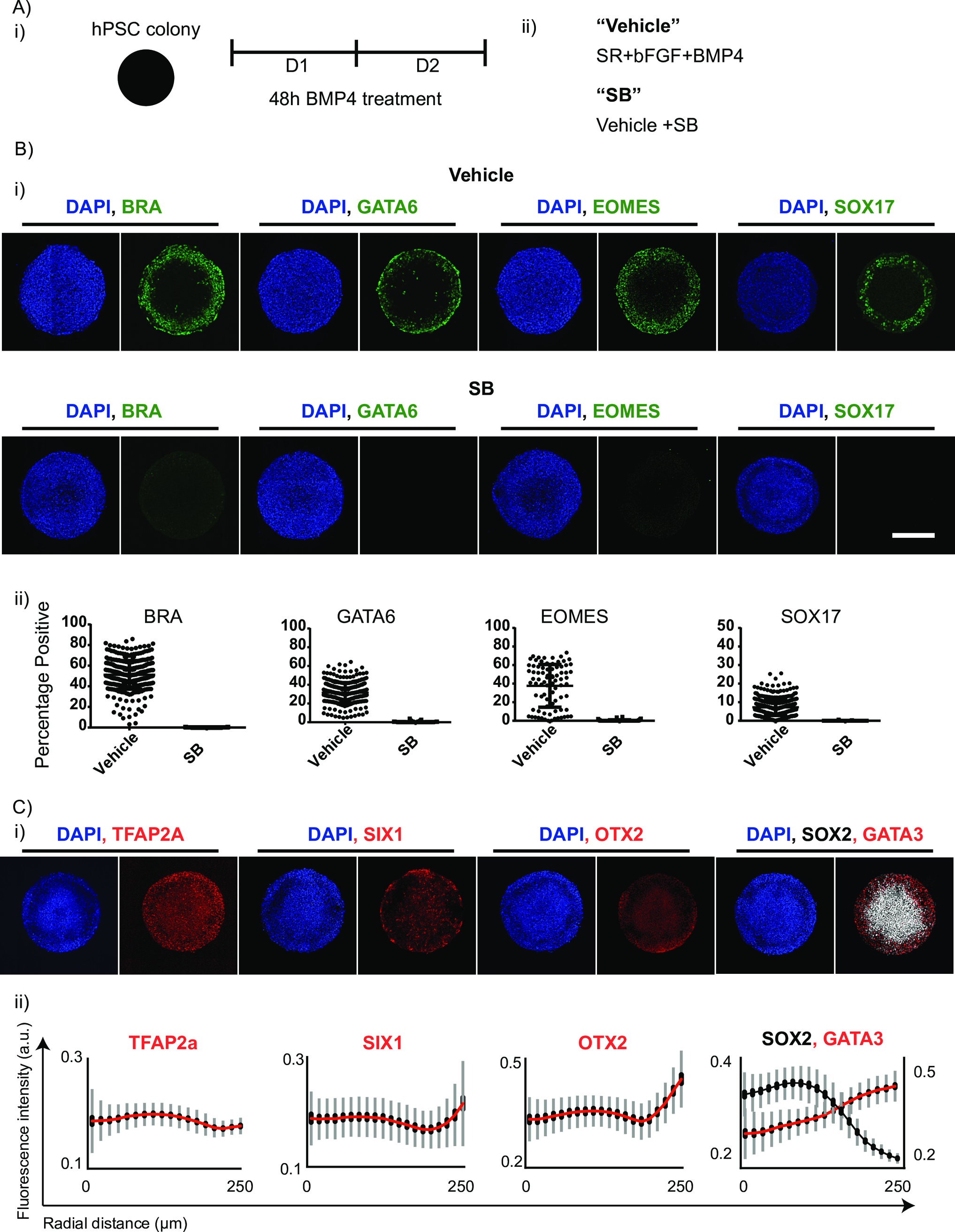

Supplement: S15 Fig — (Ai–ii) Overview of experimental setup. (i) Geometrically confined hPSC colonies were treated with BMP4 for 48 h. (ii) Media tested. ‘Vehicle’ indicated SR medium (see Materials and methods for composition) supplemented with BMP4 and bFGF. ‘SB’ indicated vehicle supplemented with 10 μM SB431542. (Bi–ii) Response of gastrulation-associated fate patterning in Vehicle and SB conditions. (i) Representative immunofluorescent images for BRA, GATA6, EOMES, and SOX17 for Vehicle and SB conditions. White triangle represents nonspecific background staining for EOMES. Scale bar represents 200 μm. (ii) Quantified expression of gastrulation-associated fates. Each data point represents an identified colony. The total number of colonies were (373, 248), (325, 317), (81, 72), and (506, 329) for BRA, GATA6, EOMES, and SOX17, respectively, for (Vehicle and SB treatments). The data are pooled from 2 experiments except for EOMES, which was performed once. (Ci–ii) Preneurulation-like fate patterning observed in the presence of SB. (i) Representative immunofluorescent images of TFAP2A, SIX1, OTX2, and co-stained image of SOX2 and GATA3. Scale bar represents 200 μm. (ii) Average radial expression intensity of the preneurulation-associated fates represented as line plots. SD shown in grey, and 95% confidence intervals shown in black. Underlying numerical data for this figure can be found in https://osf.io/zrvxj/. bFGF,; BMP4, bone morphogenetic protein 4; BRA, Brachyury; EOMES, Eomesodermin; GATA6, GATA binding protein 6; hPSC, human pluripotent stem cell; OTX2, Orthodenticle Homeobox 2; SB, SB431542; SIX1, SIX Homeobox 1; SOX17, SRY-box 17; SR, serum replacement; TFAP2A, transcription factor AP2-alpha. (TIF) [file pbio.3000081.s015.tif]

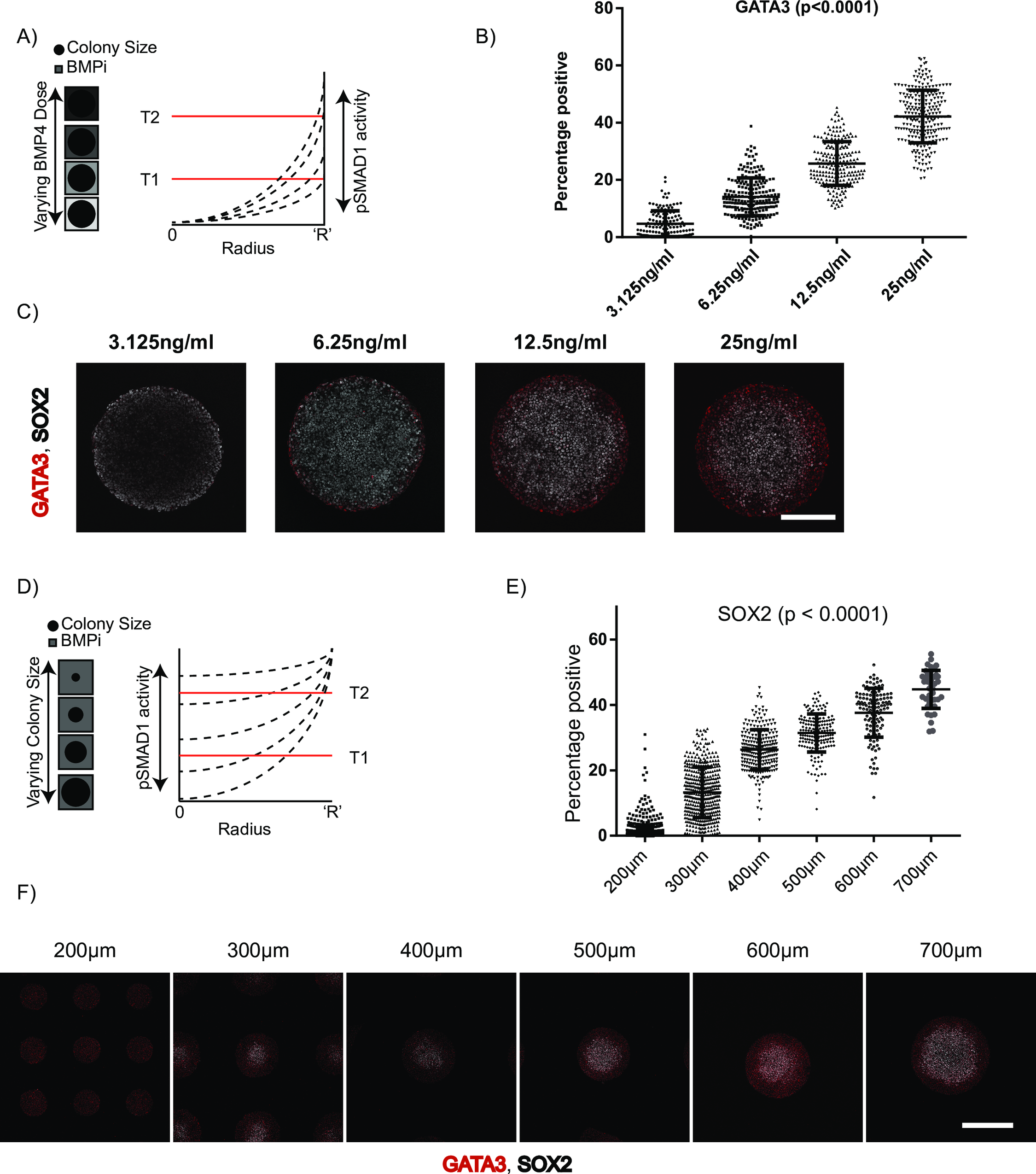

Supplement: S16 Fig — (A) Overview of experimental setup. Perturbing BMP4 dose in induction medium while maintaining colony size varies pSMAD1 concentration levels at the colony periphery (see S9A–S9C Fig for details). (B) Percentage of cells in each identified colony expressing GATA3 when colonies of 500 μm in diameter were treated with varying doses of BMP4 (3.125 ng/ml, 6.25 ng/ml, 12.5 ng/ml, and 25 ng/ml) in induction medium. Each data point represents an identified colony. The total number of colonies were 131, 208, 215, and 244 for the respective doses. Data pooled from 2 experiments. Bars represent mean ± SD. The p-value was calculated using Kruskal-Wallis test. (C) Representative immunofluorescent images of colonies stained for SOX2 and GATA3. Scale bar represents 200 μm. (D) Overview of experimental setup. Perturbing the colony size while maintaining the BMP4 dose constant in the induction medium varies the pSMAD1 levels at the colony center (see S9D Fig and S9E Fig for details). (E) Percentage of cells in each identified colony expressing SOX2 when colonies of varying sizes (200 μm, 300 μm, 400 μm, 500 μm, 600 μm, and 700 μm in diameter) were treated with 25 ng/ml of BMP4 in induction medium. Each data point represents an identified colony. The total number of colonies were 932, 439, 256, 175, 122, and 45 for the respective sizes. Data pooled from 2 experiments. Bars represent mean ± SD. The p-value was calculated using Kruskal-Wallis test. (F) Representative immunofluorescent images of colonies stained for SOX2 and GATA3. Scale bar represents 200 μm. Underlying numerical data for this figure can be found in https://osf.io/zrvxj/. BMP4, bone morphogenetic protein 4; GATA3, GATA binding protein 3; pSMAD1, phosphorylated SMAD1; SOX2, SRY box 2. (TIF) [file pbio.3000081.s016.tif]

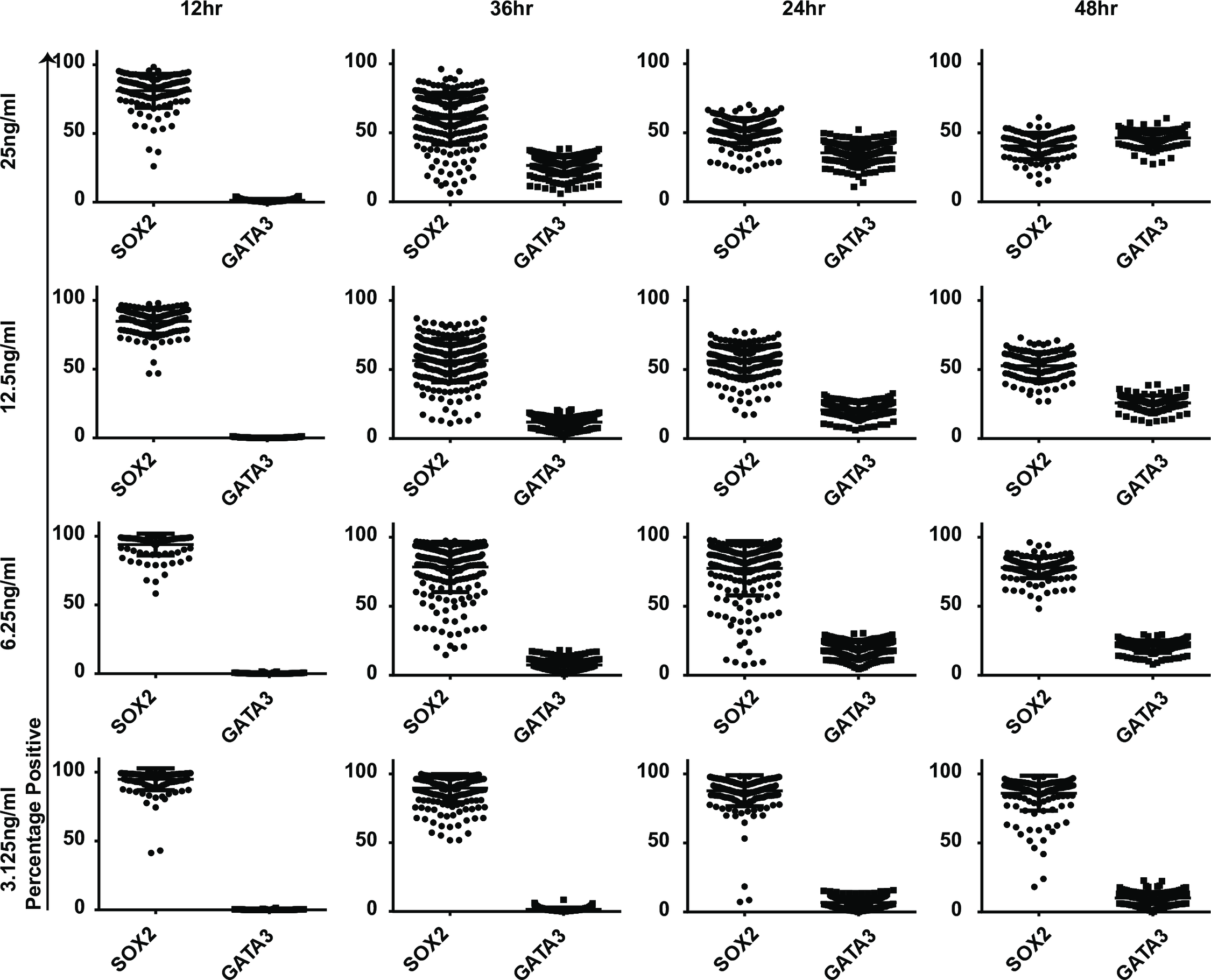

Supplement: S17 Fig — Percentage of cells expressing SOX2 and GATA in 500 μm colonies induced to differentiate at varying concentrations of BMP4 (3.125 ng/ml, 6.25 ng/ml, 12.5 ng/ml, and 25 ng/ml) and induction times (12 h, 24 h, 36 h, and 48 h). Each data point represents an identified colony, and each condition had over 100 colonies. Data pooled from 2 experiments. Bars represent mean ± SD. Underlying numerical data for this figure can be found in https://osf.io/zrvxj/. BMP4, bone morphogenetic protein 4 (TIF) [file pbio.3000081.s017.tif]

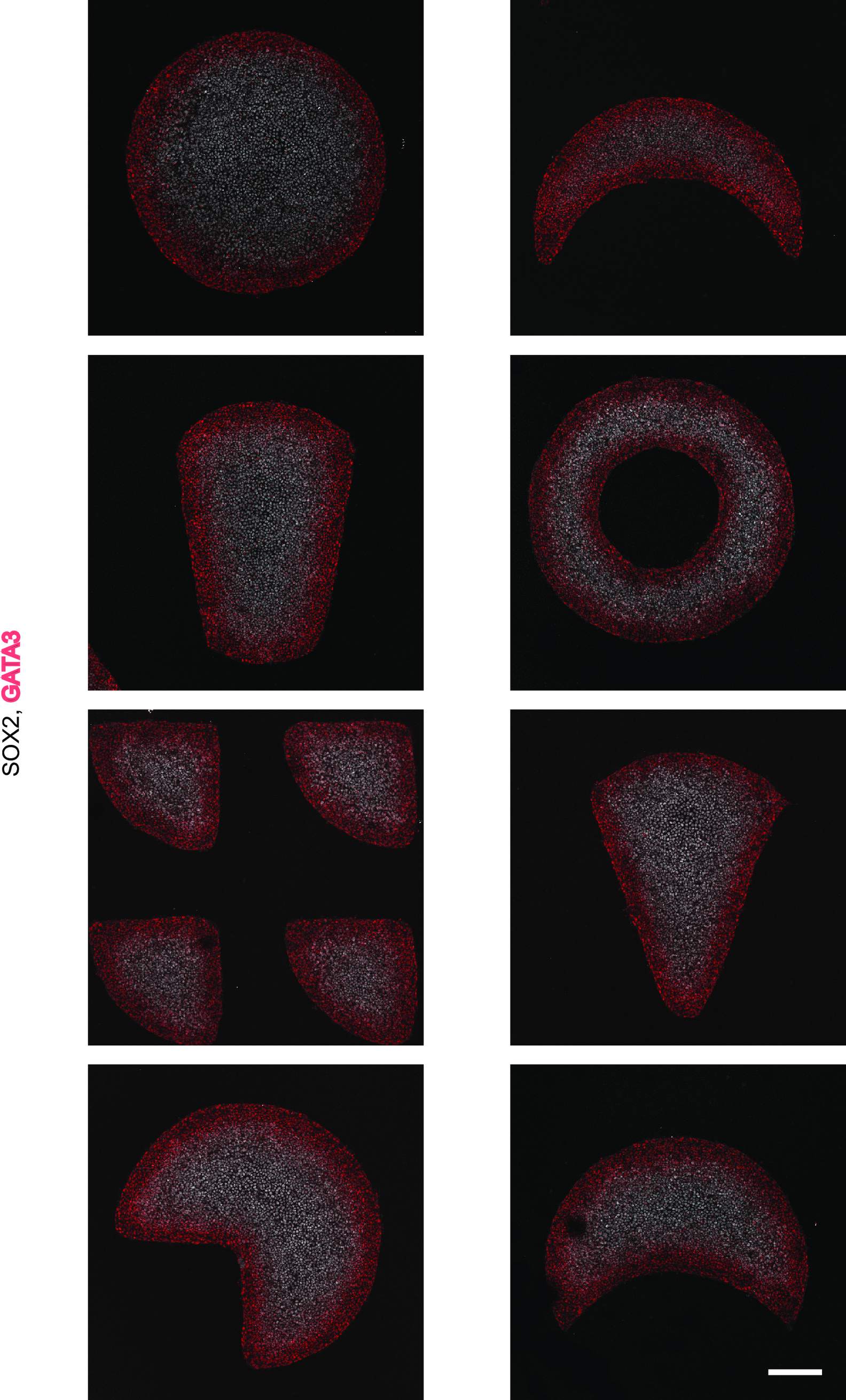

Supplement: S18 Fig — Representative images of various shapes of geometrically-confined hPSC colonies treated with BMP4 and SB in SR medium. Varying colony shapes does not result in any deviation from anticipated fate patterning. The experiment was performed once. Scale bar represents 200 μm. BMP4, bone morphogenetic protein 4; hPSC, human pluripotent stem cell; SR, serum replacement. (TIF) [file pbio.3000081.s018.tif]

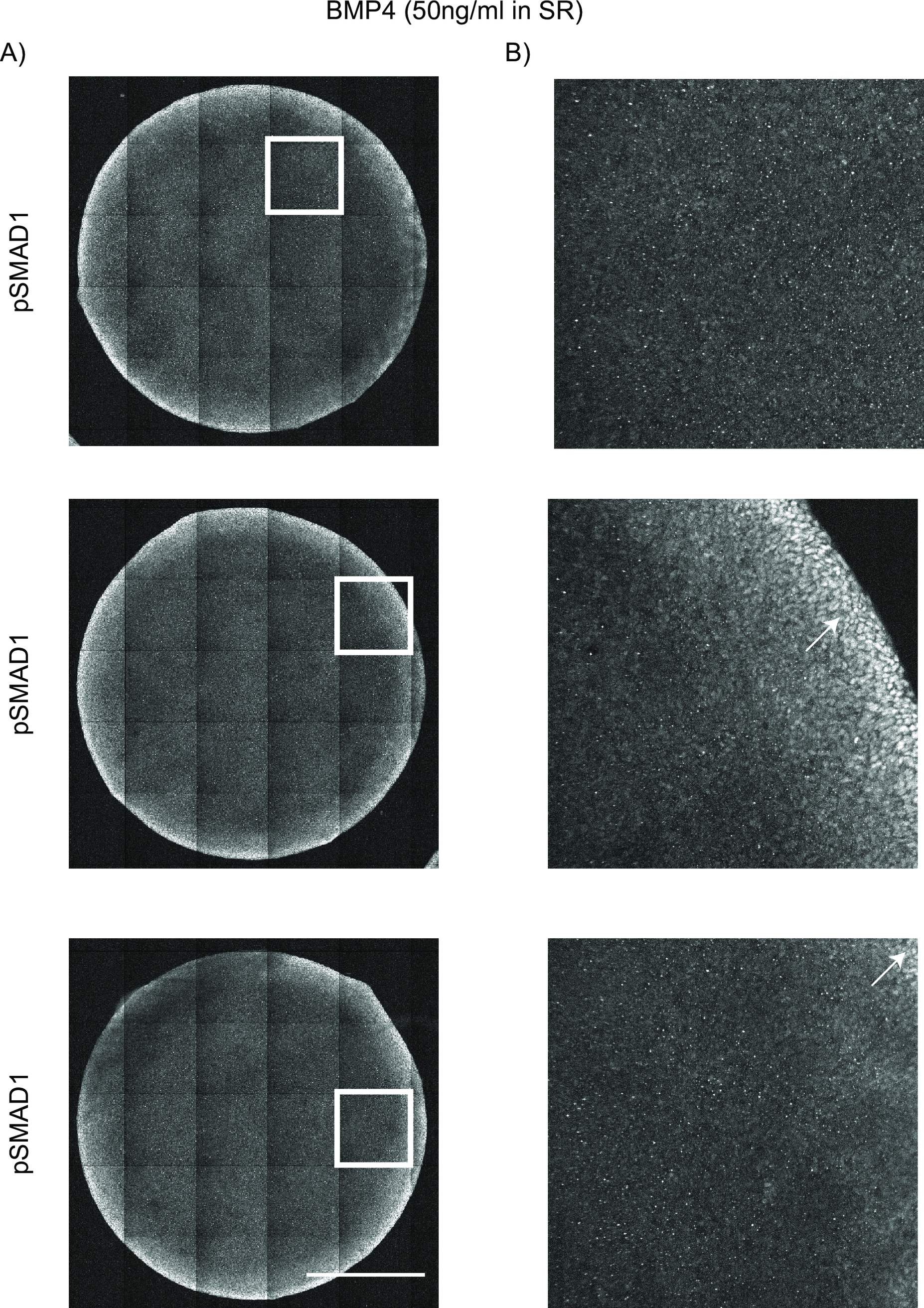

Supplement: S19 Fig — (A–B) No discernable pSMAD1 expression detected with geometrically confined hPSC colonies of 3 mm diameter were treated with 50 ng/ml of BMP4 and SB for 24 h in SR medium. (A) Stitched images of the entire colony stained for pSMAD1 shown in greyscale for ease of visibility. (B) Enlarged fields that are indicated by white squares in panel A. White arrows indicate regions that contain cells with positive pSMAD1 expression. Scale bar represents 1 mm. BMP4, bone morphogenetic protein 4; hPSC, human pluripotent stem cell; pSMAD1, phosphorylated SMAD1; SR, serum replacement. (TIF) [file pbio.3000081.s019.tif]

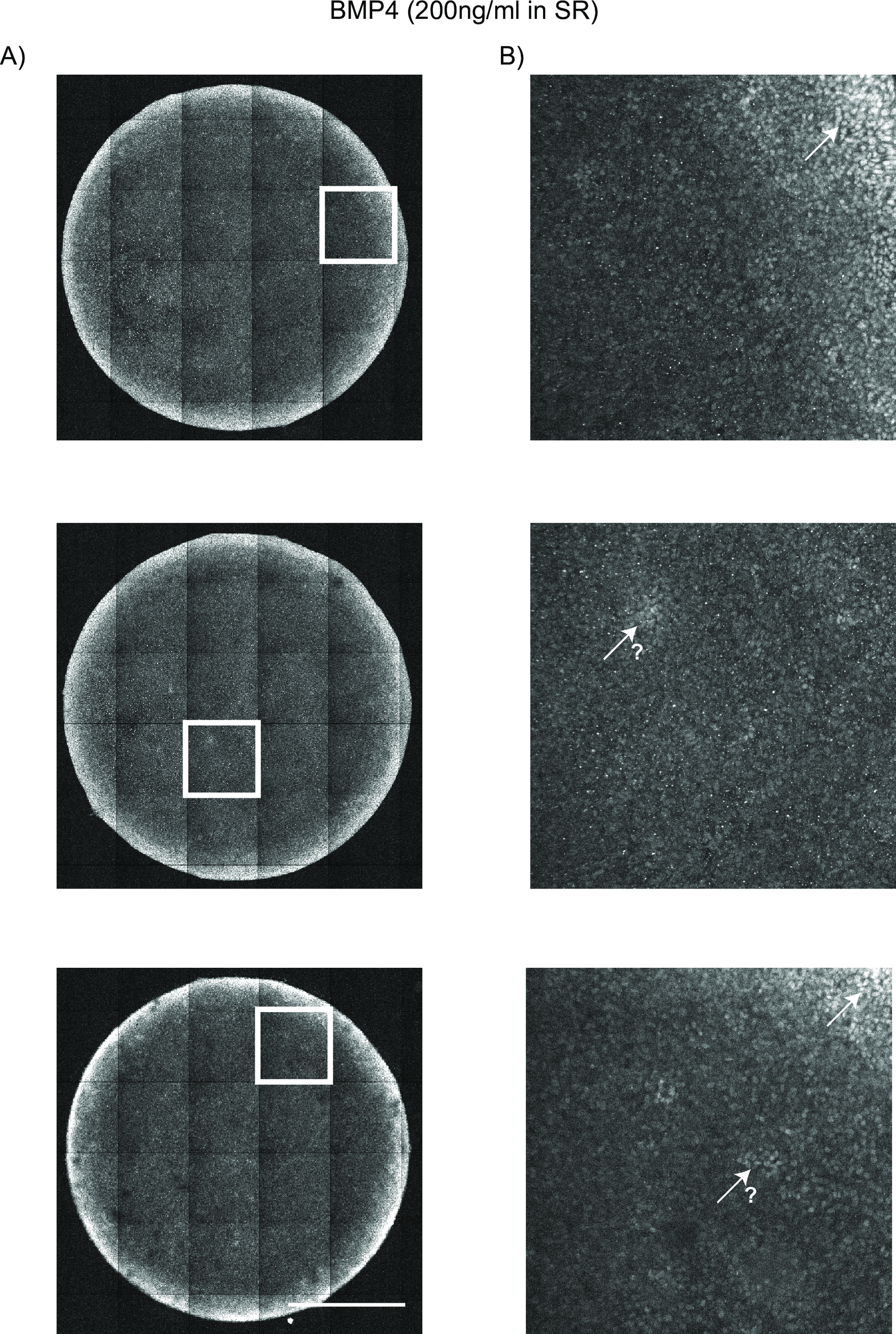

Supplement: S20 Fig — (A–B) Inconclusive staining of pSMAD1 expression detected with geometrically confined hPSC colonies of 3 mm diameter were treated with 200 ng/ml of BMP4 and SB for 24 h in SR medium. (A) Stitched images of the entire colony stained for pSMAD1 shown in greyscale for ease of visibility. (B) Enlarged fields that are indicated by white squares in panel A. White arrows at the colony periphery indicate regions that contain cells with positive pSMAD1 expression. White arrows with accompanying question marks indicate regions that possibly show expression of pSMAD1; however, the staining in these regions is inconclusive. Scale bar represents 1 mm. BMP4, bone morphogenetic protein 4; hPSC, human pluripotent stem cell; pSMAD1, phosphorylated SMAD1; SB, SB431542; SR, serum replacement. (TIF) [file pbio.3000081.s020.tif]

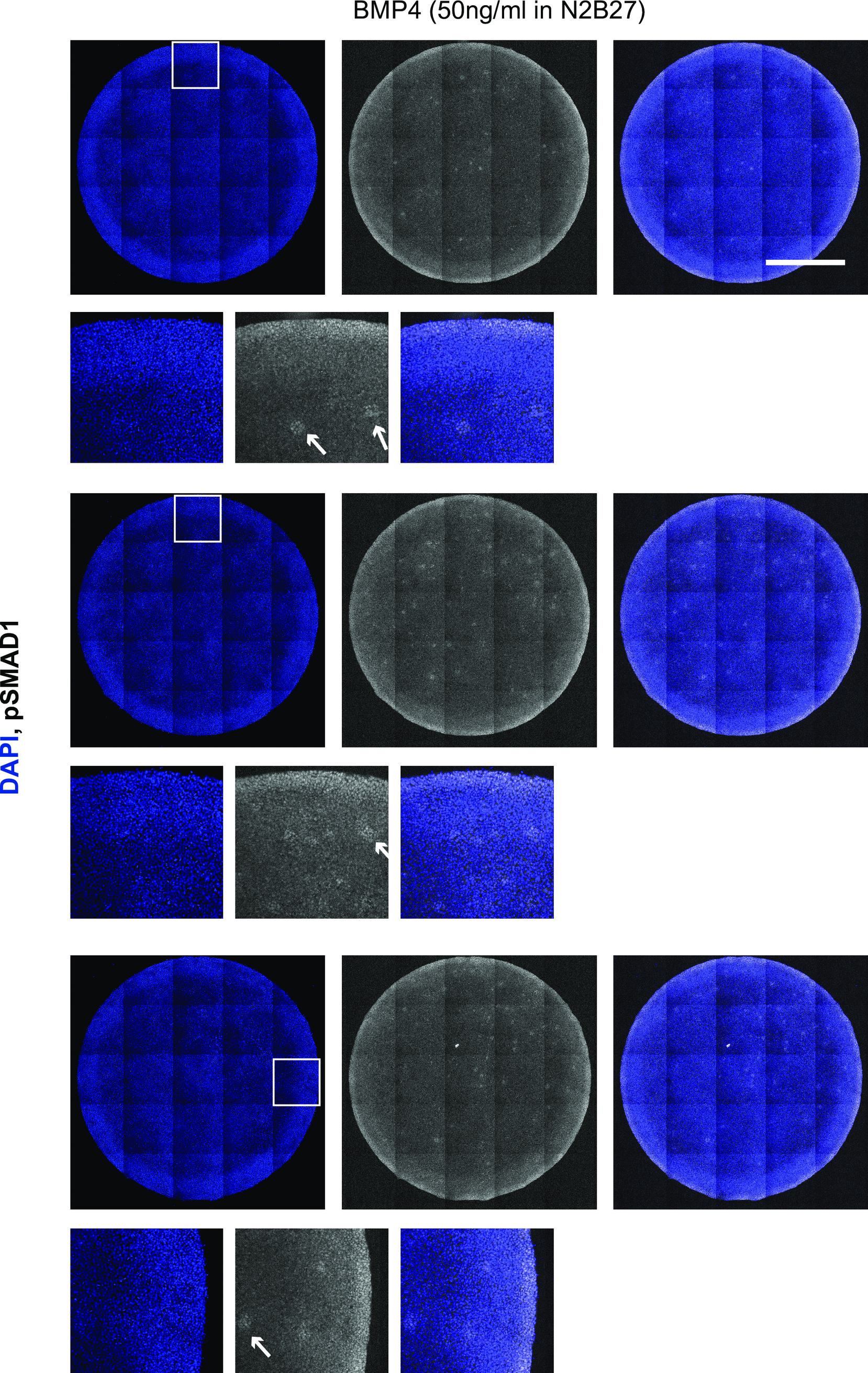

Supplement: S21 Fig — RD-like foci of pSMAD1 expression detected with geometrically confined hPSC colonies of 3 mm diameter were treated with 50 ng/ml of BMP4 and SB for 24 h in N2B27 medium. Representative stitched images of the entire colony stained for pSMAD1 shown in greyscale for ease of visibility along with DAPI staining. Enlarged fields that are indicated by white squares in stitched images. White arrows indicate regions that contain cells with positive pSMAD1 expression. Scale bar represents 1 mm. BMP4, bone morphogenetic protein 4; hPSC, human pluripotent stem cell; pSMAD1, phosphorylated SMAD1; RD, reaction-diffusion; SB, SB431542. (TIF) [file pbio.3000081.s021.tif]

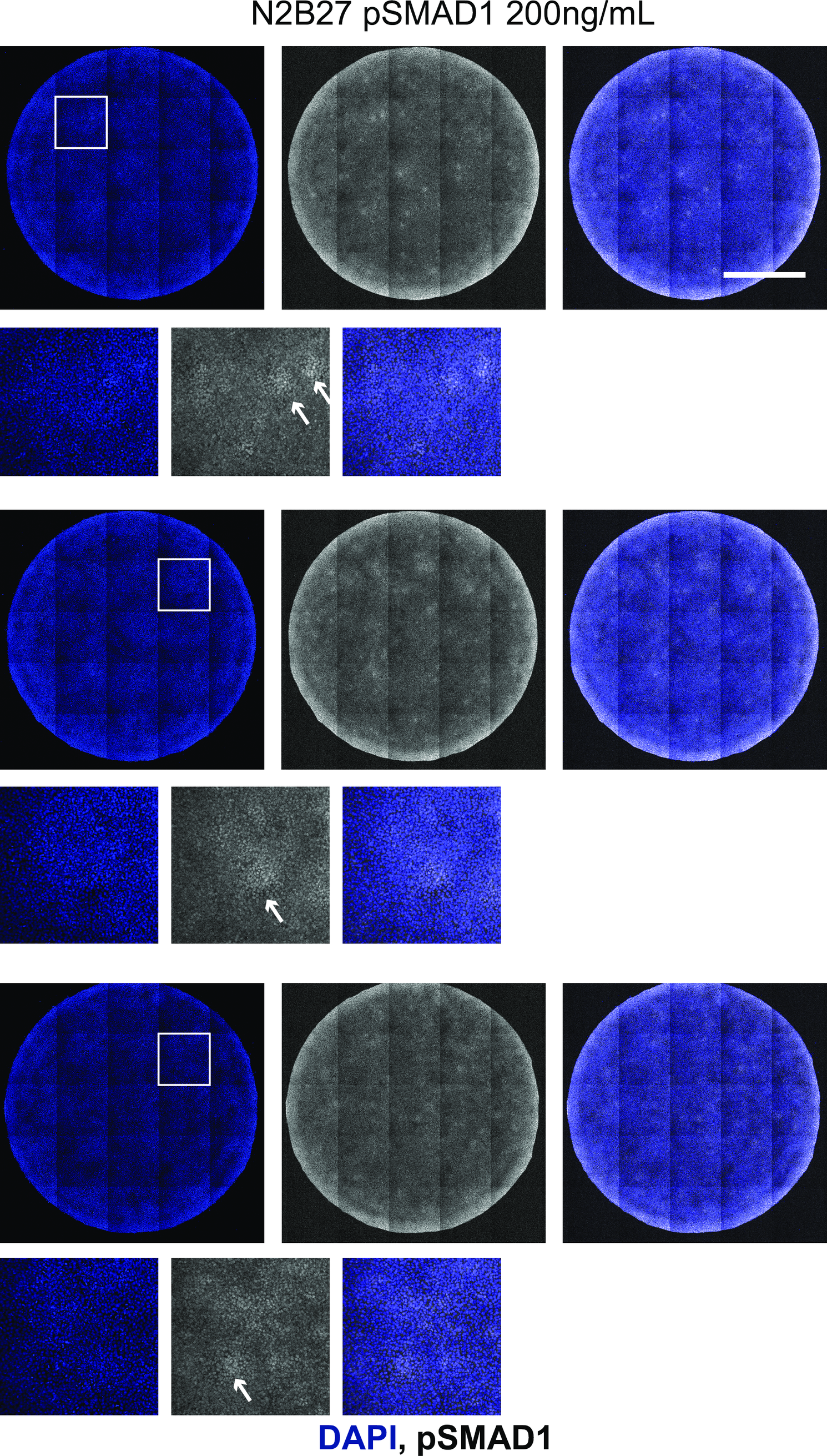

Supplement: S22 Fig — RD-like foci of pSMAD1 expression detected with geometrically confined hPSC colonies of 3 mm diameter were treated with 50 ng/ml of BMP4 and SB for 24 h in N2B27 medium. Representative stitched images of the entire colony stained for pSMAD1 shown in greyscale for ease of visibility along with DAPI staining. Enlarged fields that are indicated by white squares in stitched images. White arrows indicate regions that contain cells with positive pSMAD1 expression. Scale bar represents 1 mm. BMP4, bone morphogenetic protein 4; hPSC, human pluripotent stem cell; pSMAD1, phosphorylated SMAD1; RD, reaction-diffusion; SB, SB431542. (TIF) [file pbio.3000081.s022.tif]

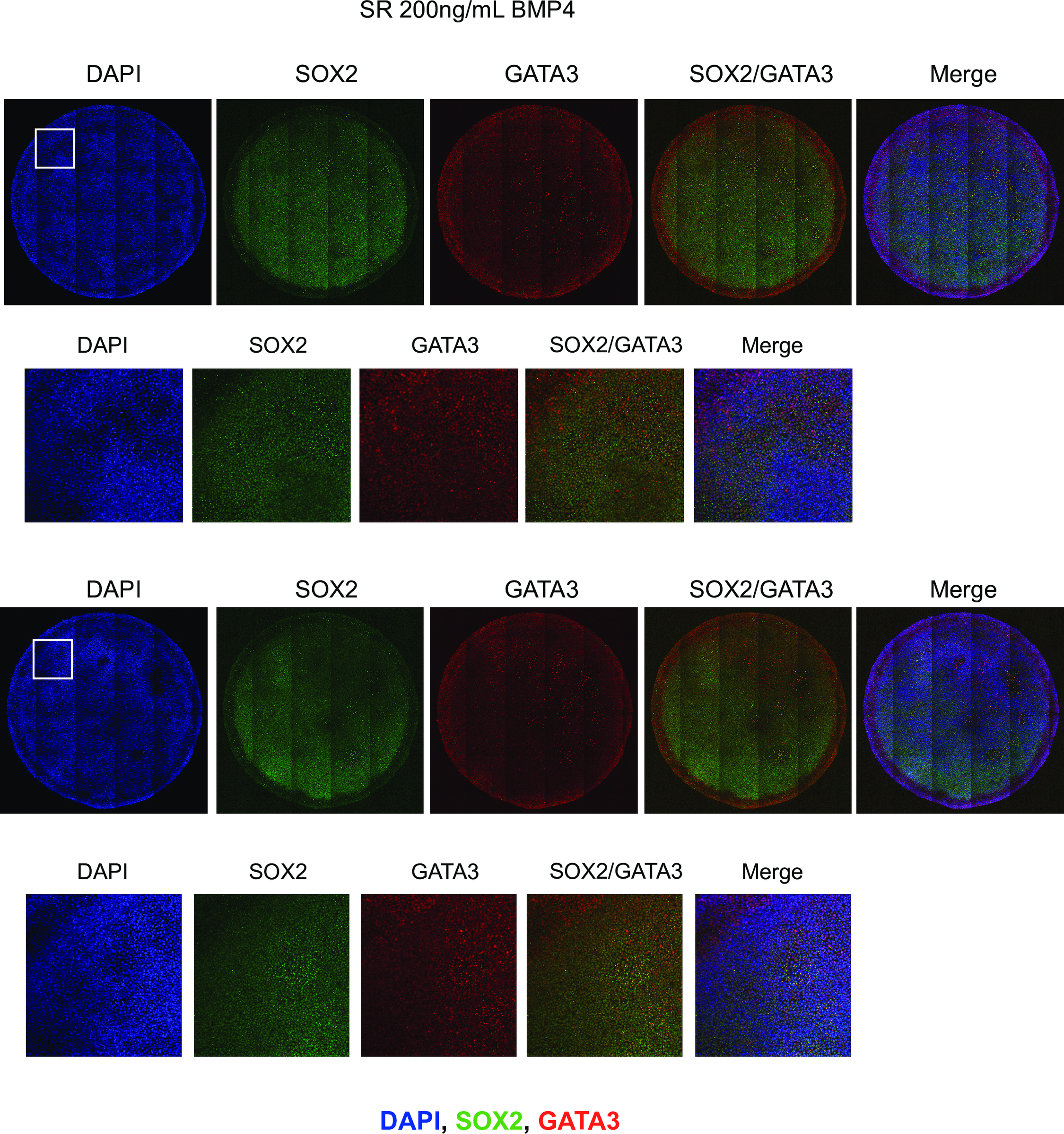

Supplement: S23 Fig — Treatment of geometrically confined hPSC colonies with 200 ng/ml of BMP4 and SB for 48 h does not display obvious RD-like foci of GATA3 expression. Representative stitched images of 3 mm diameter hPSC colonies differentiated in SR medium supplemented with 200 ng/ml of BMP4 for 48 h. Scale bar represents 1 mm. Zoomed section outlined by the white square shown below stitched colonies. The experiment was repeated 2 times. BMP4, bone morphogenetic protein 4; GATA3, GATA binding protein 3; hPSC, human pluripotent stem cell; NN, non-neural; RD, reaction-diffusion; SB, SB431542; SR, serum replacement. (TIF) [file pbio.3000081.s023.tif]

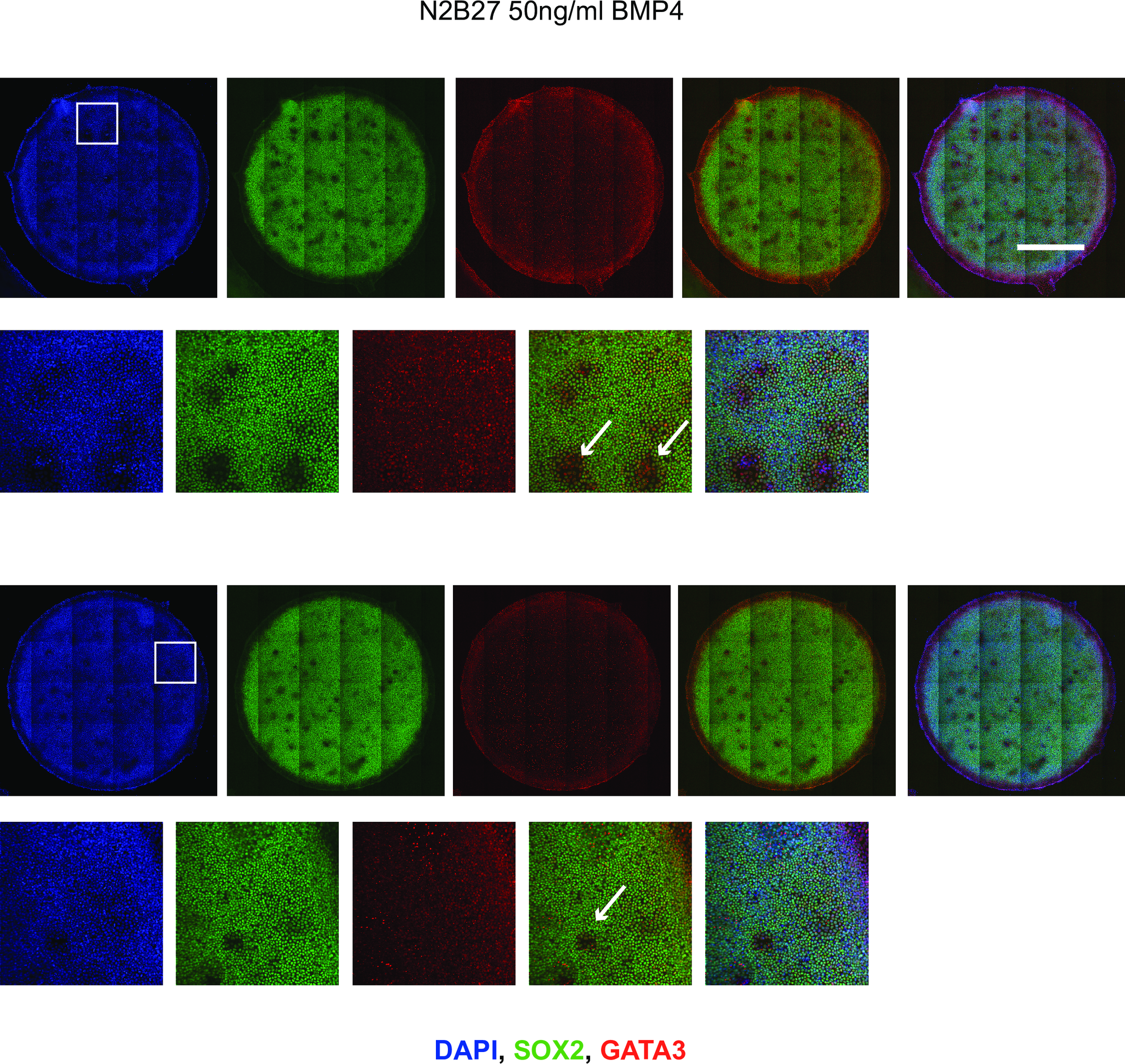

Supplement: S24 Fig — Treatment of geometrically confined hPSC colonies with 50 ng/ml of BMP4 and SB for 48 h results in RD-like foci of GATA3 expression. Representative stitched images of 3 mm diameter hPSC colonies differentiated with 50 ng/ml of BMP4 for 48 h. Scale bar represents 1 mm. Zoomed section outlined by the white square shown below stitched images. While arrows indicate GATA3 positive and SOX2 negative regions. The experiment was repeated 2 times. BMP4, bone morphogenetic protein 4; GATA3, GATA binding protein 3; hPSC, human pluripotent stem cell; NN, non-neural; RD, reaction-diffusion; SB, SB431542; SOX2, SRY box 2. (TIF) [file pbio.3000081.s024.tif]

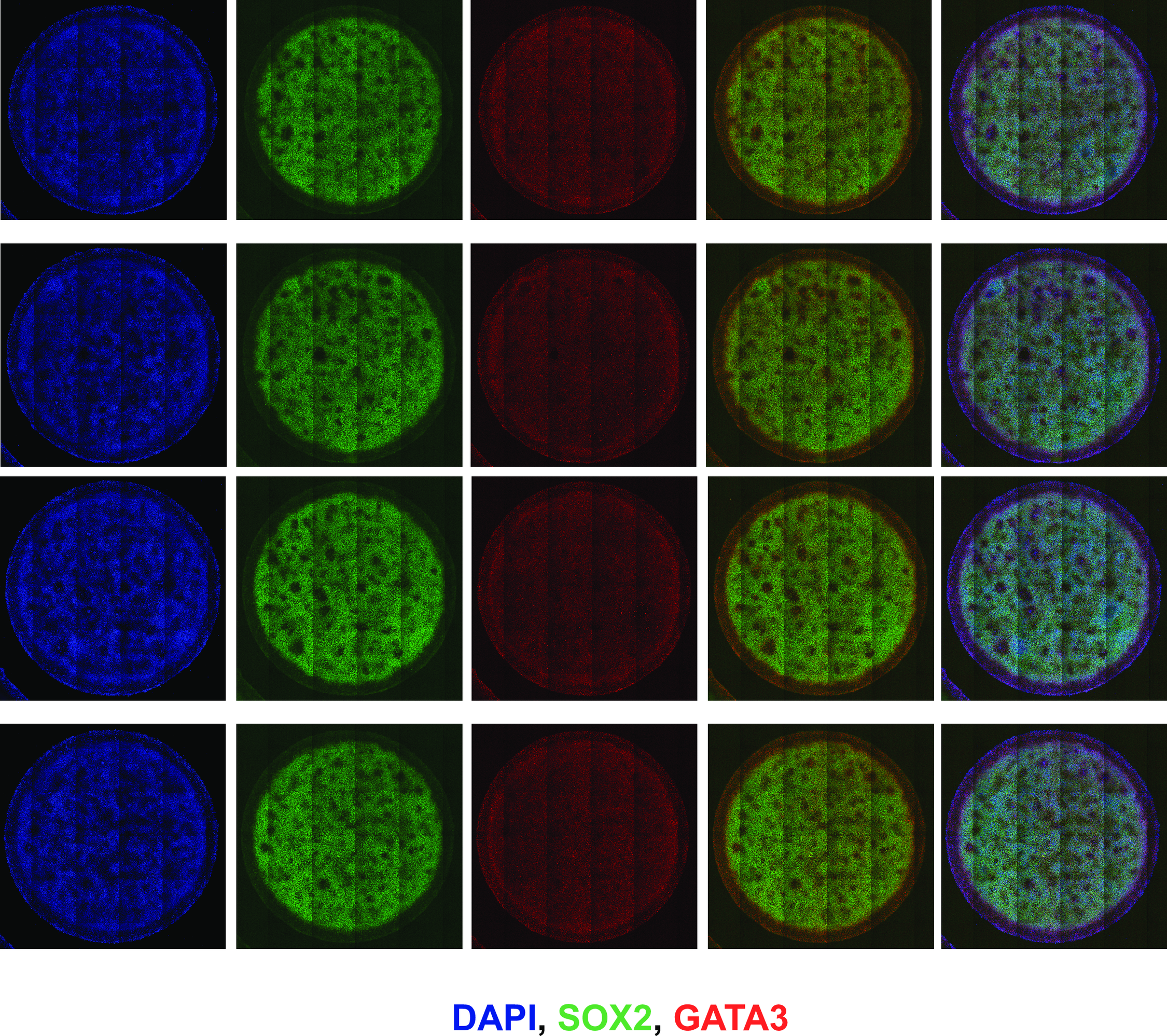

Supplement: S25 Fig — Representative immunofluorescent images of geometrically confined hPSC colonies of 3 mm diameter stained for SOX2 and GATA3. The colonies were treated with 200 ng/ml of BMP4 and SB for 48 h. Scale bar represents 1 mm. BMP4, bone morphogenetic protein 4; GATA3, GATA binding protein 3; hPSC, human pluripotent stem cell; NN, non-neural; RD, reaction-diffusion; SB, SB431542; SOX2, SRY box 2. (TIF) [file pbio.3000081.s025.tif]

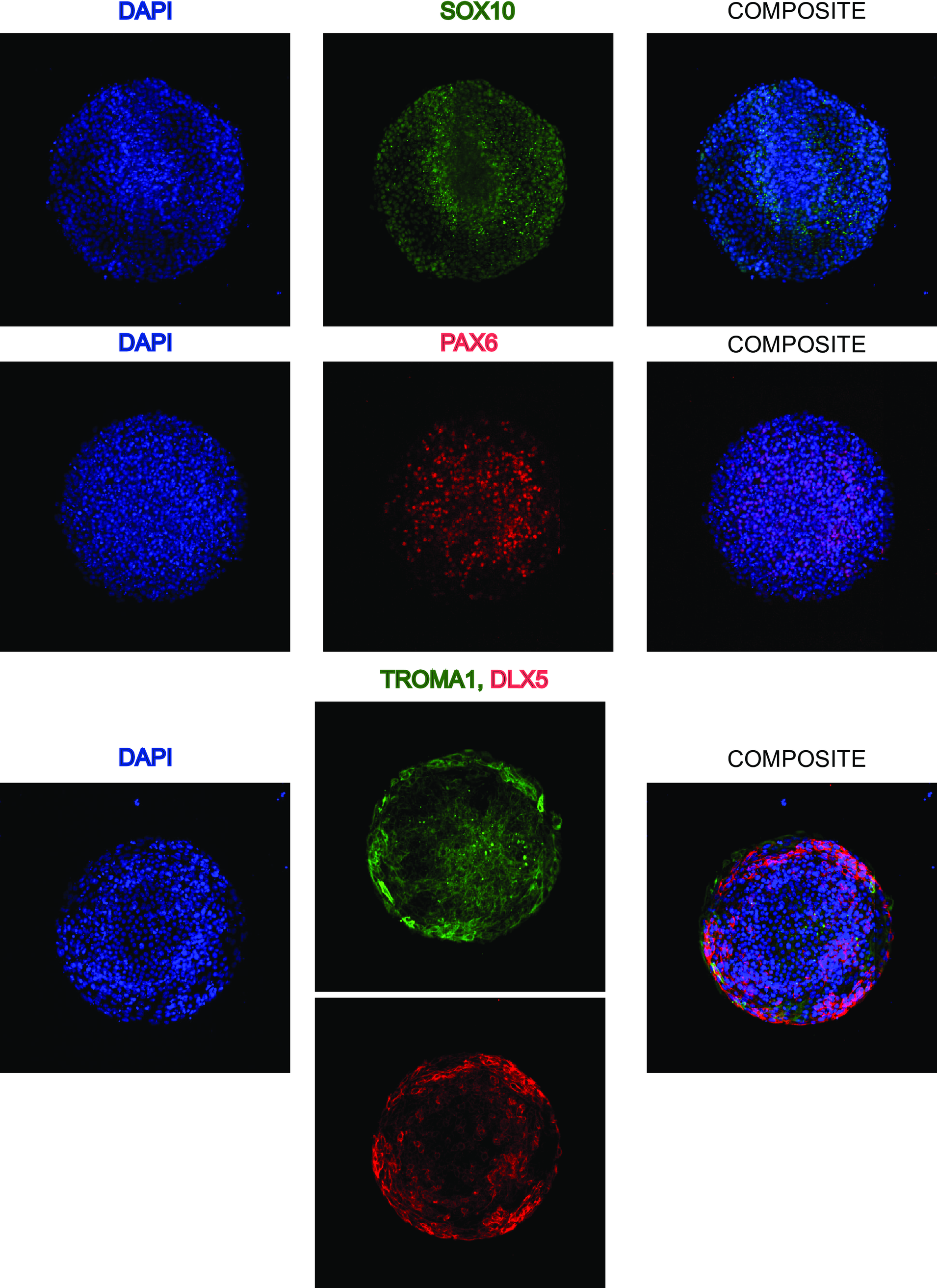

Supplement: S26 Fig — (A) Representative image of colony treated with BMP4 and SB for 24 h and NOG + SB for 72 h (see Fig 7A) stained for DAPI and PAX6. (B) Representative image of colony treated with BMP4 and SB for 24 h and CHIR + SB for 48 h (see Fig 7A) stained for DAPI and SOX10. (C) Representative image of colony treated with BMP4 and SB for 72 h (see Fig 7A) stained for DAPI, TROMA1, and DLX5. BMP4, bone morphogenetic protein 4; CHIR, CHIR99021; DLX5, Distal-Less Homeobox 5; NOG, Noggin; PAX6, Paired Box 6; SB, SB431542; SOX10, SRY-box 10; TROMA1, Cytokeratin-8 antibody clone. (TIF) [file pbio.3000081.s026.tif]

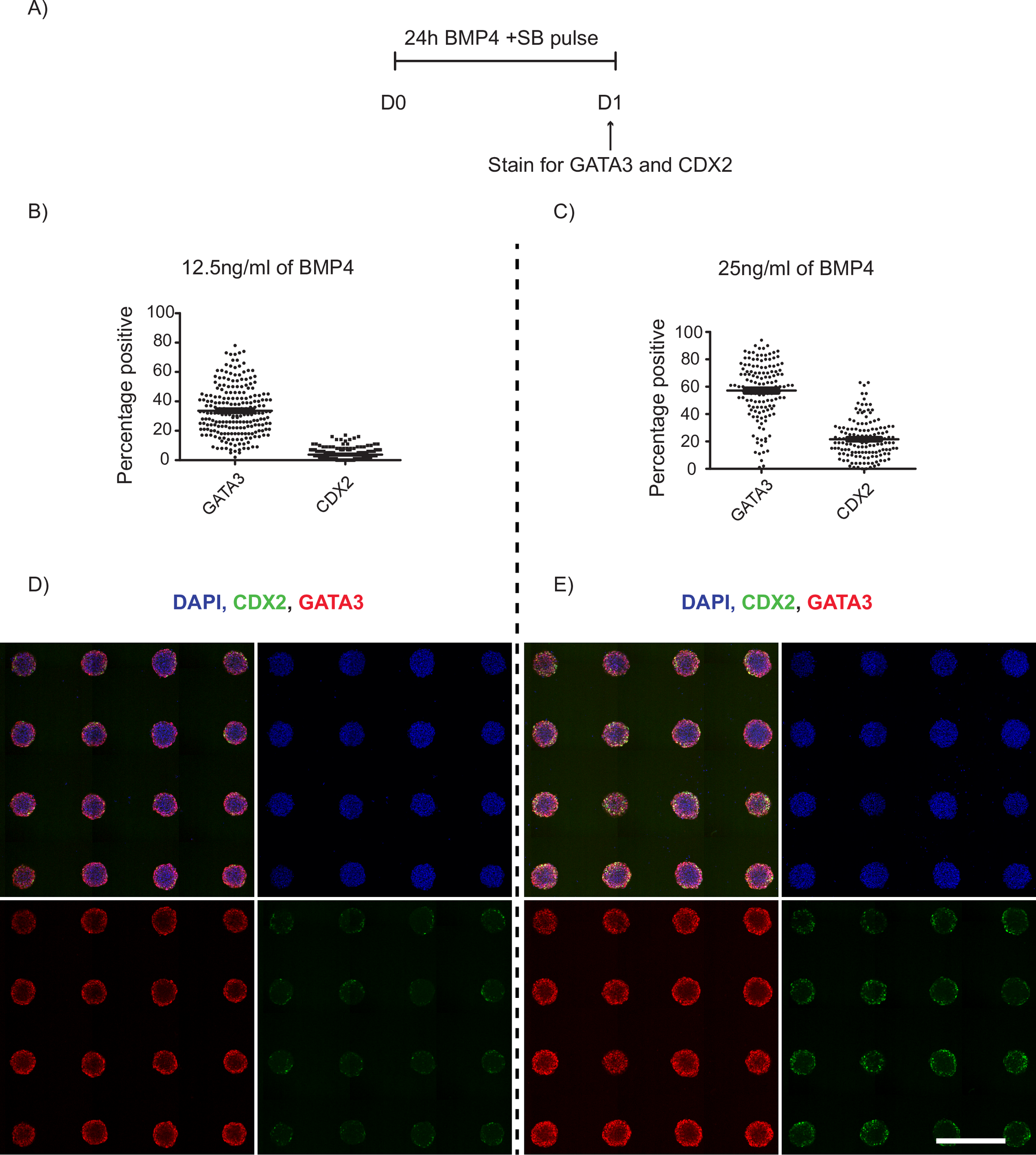

Supplement: S27 Fig — (A) Overview of experimental setup. Geometrically confined colonies of 200 μm diameter were treated with BMP4 and SB at concentrations of 12.5 ng/ml and 25 ng/ml for 24 h and stained for CDX2 and GATA3. (B–C) Quantified expression of CDX2 and GATA3 detected in the geometrically confined colonies treated with BMP4 and SB reveals that GATA3 expression surpasses that of CDX2. (B) A total of 220 colonies treated with 12.5 ng/ml of BMP4. (C) A total of 141 colonies treated with 25 ng/ml of BMP4. (D–E) Representative immunofluorescent images of colonies (from B and C) stained for DAPI, GATA3, and CDX2. Scale bar represents 500 μm. Underlying numerical data for this figure can be found in https://osf.io/zrvxj/. BMP4, bone morphogenetic protein 4; CDX2, Caudal Type Homeobox 2; GATA3, GATA binding protein 3; SB, SB431542. (TIF) [file pbio.3000081.s027.tif]

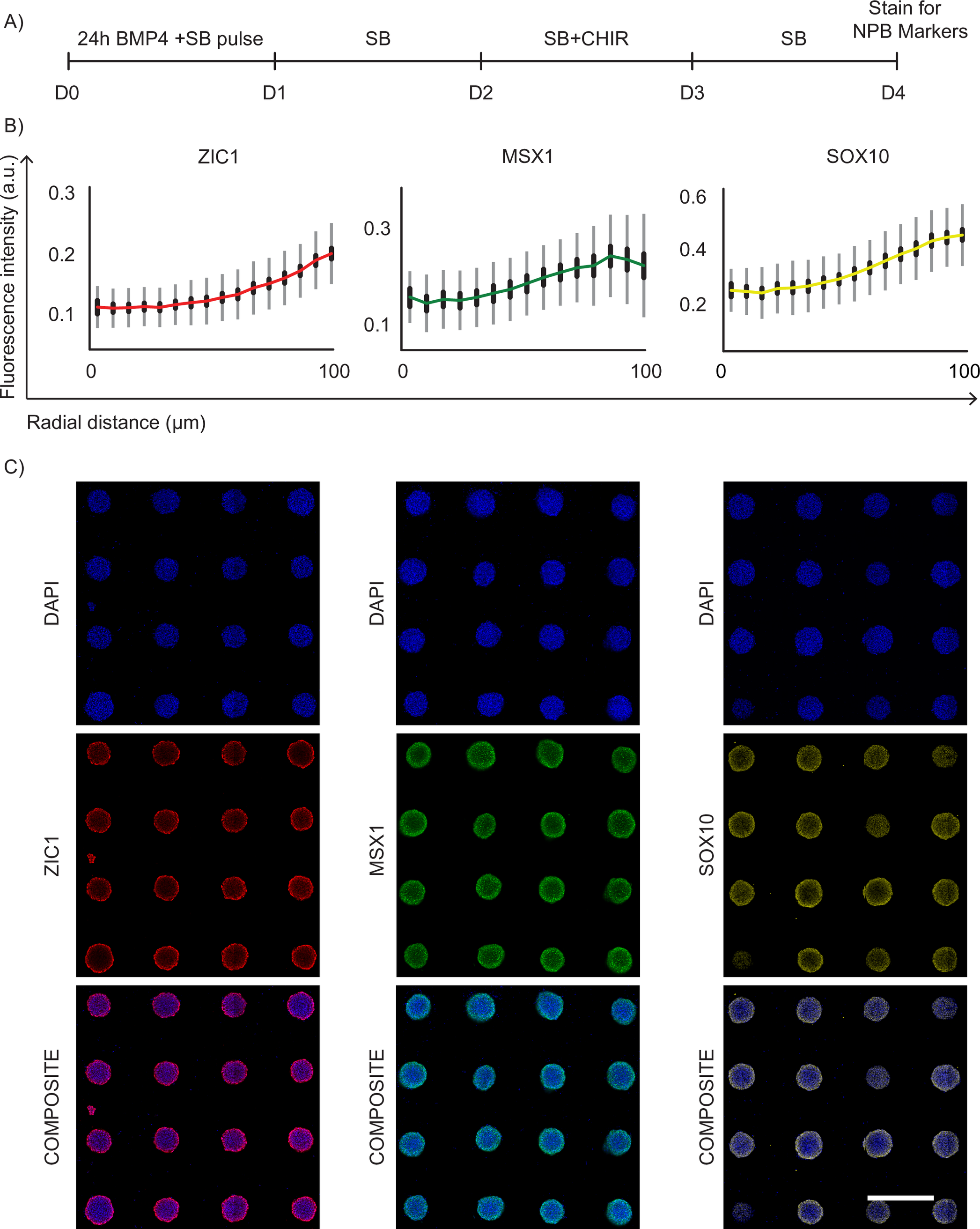

Supplement: S28 Fig — (A) Overview of experimental setup. Geometrically confined hPSC colonies were treated with SB and 25 ng/ml of BMP4 for 24 h and then were differentiated with an adapted protocol that has been reported to generate NPB identities (Xue and colleagues, 2018). The differentiation protocol consisted of treating with SR medium supplemented with SB for 3 days with a transient 24 h pulse of CHIR (3 μM). At D4, the colonies were fixed and stained for ZIC1, MSX1, and SOX10. (B) Average expression of ZIC1, MSX1, and SOX10 shown as line plots in red, green, and yellow, respectively. The SDs are shown in black, and the 95% confidence intervals are shown in grey. The number of colonies included in the overlays were 119 for ZIC1, 181 for MSX1, and 116 for SOX10. (C) Representative immunofluorescent images for ZIC1, MSX1, and SOX10. Scale bar represents 500 μm. Underlying numerical data for this figure can be found in https://osf.io/zrvxj/. BMP4, bone morphogenetic protein 4; CHIR, CHIR99021; hPSC, human pluripotent stem cell; MSX1, Msh homeobox 1; NPB, NP border; SOX10, SRY box 10; SR, serum replacement; ZIC1, Zic Family Member 1. (TIF) [file pbio.3000081.s028.tif]
