# Supplementary material for: High-throughput micropatterning platform reveals Nodal-dependent bisection of peri-gastrulation–associated versus preneurulation-associated fate patterning
Source: PLoS Biol. 2019 Oct 21;17(10):e3000081. doi: 10.1371/journal.pbio.3000081 (PMC6822778; doi:10.1371/journal.pbio.3000081)
Supplement: S1 Text — (DOCX) [file pbio.3000081.s032.docx]

**Supplementary Materials:**

**RD model**

To model the effect of Nodal signalling in the regulation of the pSMAD1 organization, we employed the RD model reported in our previous study[1] which described a simplified activator-inhibitor system regulating the interaction between BMP4 (activator) and Noggin (inhibitor) (model overview shown in **Fig. 4H**). We updated this model by supplementing an inhibitory term added to the BMP production function (*e_BMP_* in *F* – described below in **Fig. SM1**) in the case when Nodal signalling was permitted. This term allowed us to incorporate the role played by BMP antagonists downstream of Nodal signalling (overview shown in **Fig. 8**) in the organization of the pSMAD1 signalling gradient within the geometrically confined hPSC colonies. The experimentally determined Nodal profile (**Fig. S11A**) was employed as a guide to describe the static spatial profile of the BMP inhibitors (***inh(x)***) that would arise downstream of Nodal signalling. This inhibitory term (**Fig. SM2**) was defined as a function of space and time and mirrored the SMAD2 expression profile observed (**Fig. S11A**). A dynamic version of the profile – ***inh(x,t)*** (shown below) – was included in the production functions only in the case when we sought to model the response of BMP signalling organization in the presence of Nodal activity. In the case when Nodal was inhibited, the term was not included in the production function.

$$inh\left( x,t \right)=inh\left( x \right)\times(1- e^{\frac{(t-t_{0})}{\tau}})\times\theta(t-t_{0})$$

In the equation above, t_0_ and $\tau$ are the initiation time and the timescale of the gradient formation, respectively, and θ is the Heaviside-theta function. We tested the robustness of this dynamical effect by scanning $\tau$ over six orders of magnitude (20 values for $\tau$ equally distributed in the range 0-106 seconds), and a wide range of t_0_ (4 values equally distributed in the range 0-106 seconds). We found that the output profiles of pSMAD1 attained were robust to these dynamics parameters.

Notably, the previous model employed the BMP dose in the induction medium as a boundary condition (“***BMP_out_***”). Given our observations of variations in the pSMAD1 levels at the periphery of the colonies as a function of Nodal activity (**Fig. 5A-C**), we updated the definition of the boundary condition as BMPout in the based on empirically determined values for pSMAD1 staining. Please see **Fig. SM1** below for mathematical description of the boundary condition.


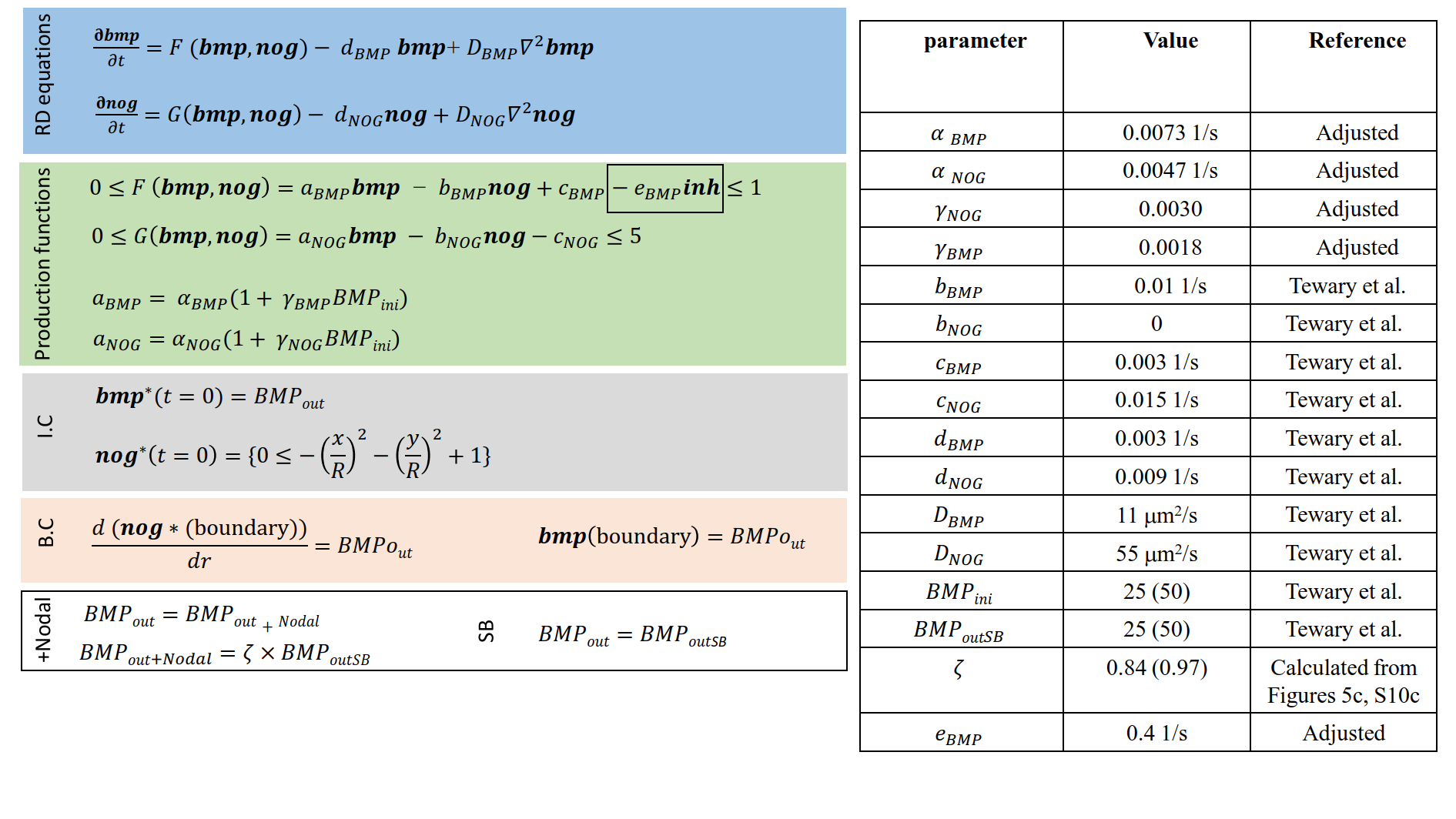


**Figure SM1:** Mathematical description of the RD equations employed in the simulations. The overall RD equations employed are shown in the blue box, the production functions are shown in the green box. The black rectangular outline highlights the inhibitory term added to describe the contribution to the inhibitor pool provided by BMP antagonists downstream of Nodal signalling. The grey box shows the initial conditions employed in the model. Notably, the initial conditions are robust to the profile of ‘nog*(t)’. As such, random profiles of the inhibitor pool result in identical responses. The boundary conditions are shown in the light orange rectangle. The mathematical definitions of BMP_out_ in Nodal and SB conditions are shown in the box outlined in black. The parameter values employed in the study were adapted from our previous reported model and are shown in the table on the right. All simulations in the study were performed using these parameter values.


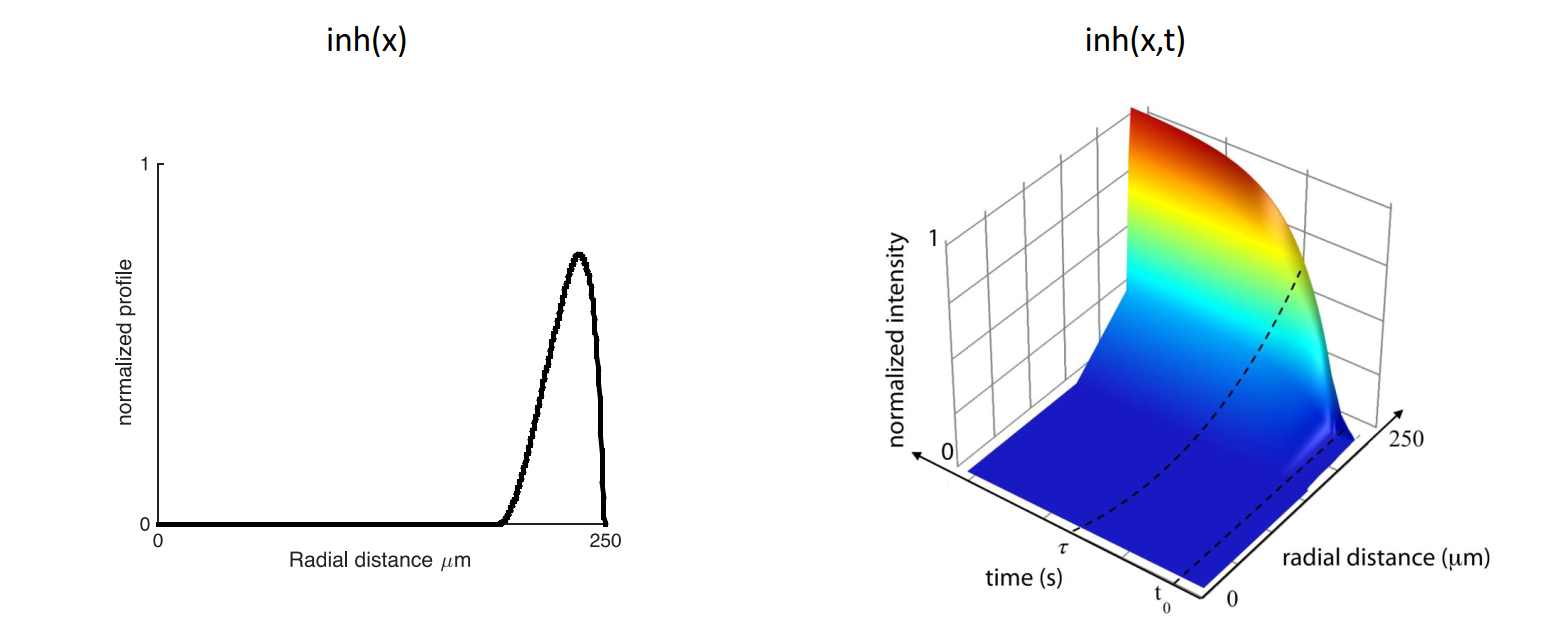


**Figure SM2**: The spatial profiles of employed that describe Nodal inhibition. The static profile inh(x) shown on the left mirrors the SMAD2 expression profile observed (Fig. S11A), and the dynamic profile on the left was employed to test the robustness of the model response to the dynamics of this profile.

**Image analysis**

In order to assess if the distribution of the BMP signalling foci in our experimental data had an underlying order, we compared the distributions of distances to the five nearest neighbours of each BMP signalling peak in the experimental data (as detected by the absence of SOX2 expression) with random profiles and profiles extracted from the simplified RD model reported in this study as for parameter e_bmp_=0. To extract the distance distributions from the valleys in Sox2 intensity, experimental images acquired of 3mm diameter colonies treated with N2B27 supplemented with SB and 200ng/ml of BMP4 were compared with 3 modelled exact one-frequency periodic distributions and 10 random distributions. Spots have been extracted from experimental images by employing the following image analysis pipeline. The images were inverted, and the intensities were rescaled; the image was then binarized via adaptive thresholding. For each colony, the maximum rectangular region that could be inscribed was cropped. Connected regions were then identified in these cropped images, and the distances between the centroids were calculated. The image processing was conducted using scikit-image [2] and the distance calculations was performed with scikit-learn [3]. Random distributions of centroids were created using numpy [4] and exact periodic patterns were created in comsol using our previously published reaction diffusion model. The nearest cell distances of experimental results somewhat departed from the idealized periodic distribution and displayed less dispersion than the distance distributions from the randomly generated profiles, as can be seen in the figure below.


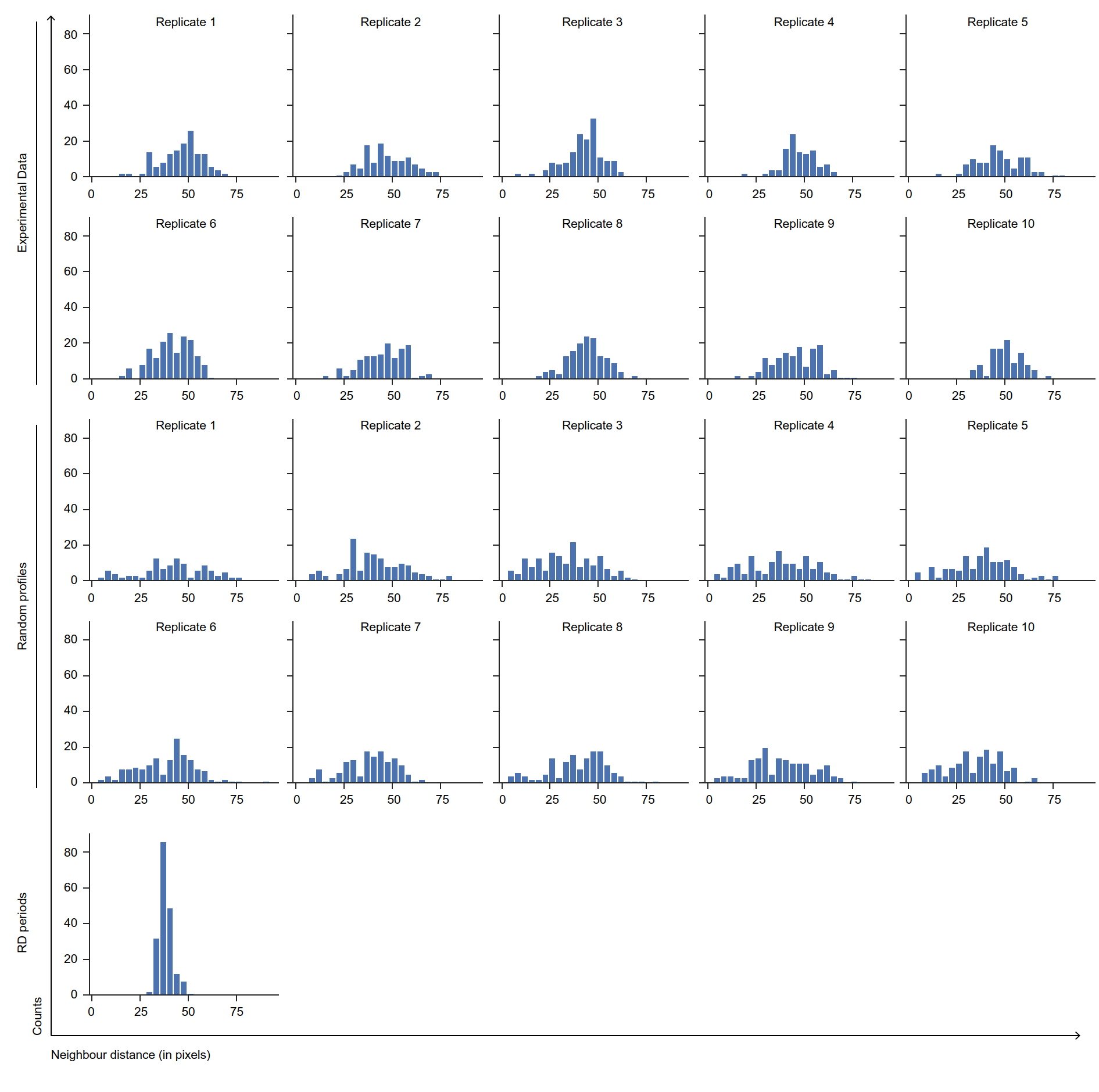


**Fig. SM3**: Histograms showing the distance distributions of experimentally observed foci of BMP signalling (first two rows), randomly generated distributions (third and fourth rows), and the distributions observed in our RD model (last row).

The neighbour distances for each spot in each condition show similar clustering of the three groups (individual neighbour distances in blue, mean neighbour distance in red):


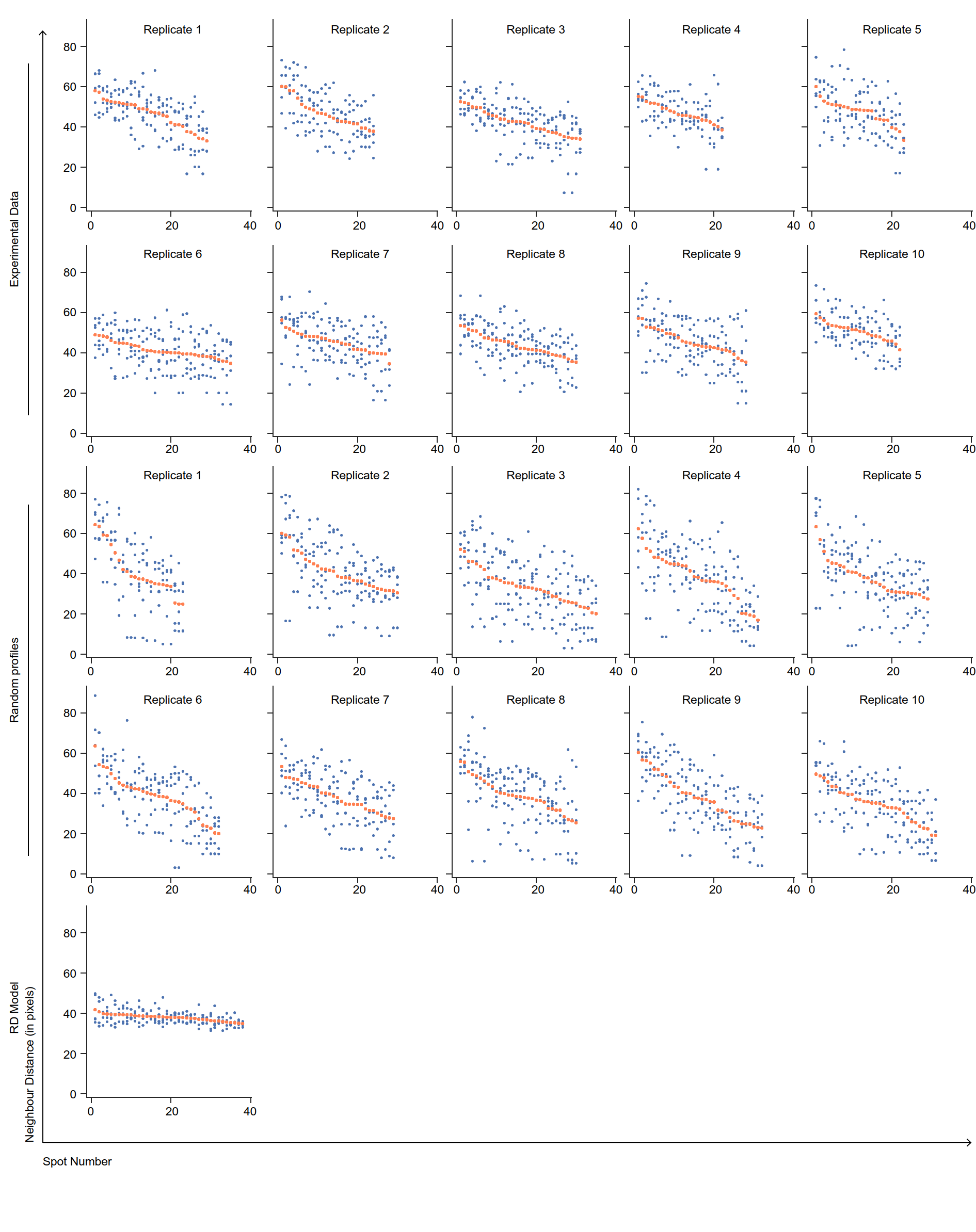


**Figure SM4:** The neighbour distances observed for each focal point of BMP signalling shown for the experimental, random, and deterministic RD condition.

We sought to employ the entropy [5] of these distance distributions as an initial measure to compare the experimental distributions with the periodic and random samples. The average entropies and standard deviations can be seen on the following table:

| Entropy | **Average** | **StDev** | **# instances** |
| --- | --- | --- | --- |
| **Periodic** | 1,145144 | 0,069481 | 3 |
| **Experimental** | 3,088798 | 0,177558 | 10 |
| **Random** | 3,524759 | 0,121471 | 10 |

Table SM1: Average entropies and standard deviations of the experimental image data and the random distributions.

The p-values between all 3 cases are under 0.001, indicating that these are all significantly different from each other. This can be interpreted as experimental distance distributions being neither random nor similar to the periodic distribution that is observed in the simplified mathematical model that we employed.

To understand what information in the experimental distributions is common with the rest, we measured the Symmetrical Uncertainty (SU) [6] for all instances of the three types of distributions. The SUs obtained when comparing periodic, experimental and random distributions with themselves are shown in the table below:

| SU | **Average** | **StDev** | **# instances** |
| --- | --- | --- | --- |
| **Per-Per** | 0,948233 | 0,000404 | 6 |
| **Exp-Exp** | 0,703027 | 0,190484 | 90 |
| **Rand-Rand** | 0,474641 | 0,056449 | 90 |

Table SM2: Symmetrical Uncertainty (SU) of the experimental image data and the random distributions

As expected, periodic instances have a very high correlation with each other, as they contain very similar information about the distribution, given the deterministic nature of the model. Experimental results are past 70% in SU, indicating the pattern in different experimental instances retain a significant amount of the same information. Again, the p-values between all 3 cases are under 0.001, indicating that they are differentiated comparisons.

Finally, SU is measured between different types of instances:

| SU | **Average** | **StDev** | **# instances** |
| --- | --- | --- | --- |
| **Per-Exp** | 0,341629 | 0,110418 | 30 |
| **Per-Rand** | 0,163981 | 0,042275 | 30 |
| **Exp-Rand** | 0,415921 | 0,074427 | 100 |

Table SM3: SU between different types of datasets

Exact periodic patterns have more information in common with experimental results than with random patterns. As before, the p-values between all 3 cases are under 0.001, indicating that they are differentiated comparisons.

References:

1. Tewary M, Ostblom J, Prochazka L, Zulueta-Coarasa T, Shakiba N, Fernandez-Gonzalez R, et al. A stepwise model of Reaction-Diffusion and Positional-Information governs self-organized human peri-gastrulation-like patterning. Development
2. Stéfan van der Walt, Johannes L. Schönberger, Juan Nunez-Iglesias, François Boulogne, Joshua D. Warner, Neil Yager, Emmanuelle Gouillart, Tony Yu, and the scikit-image contributors. scikit-image: Image processing in Python. PeerJ 2:e453 (2014) https://doi.org/10.7717/peerj.453
3. Fabian Pedregosa, Gaël Varoquaux, Alexandre Gramfort, Vincent Michel, Bertrand Thirion, Olivier Grisel, Mathieu Blondel, Peter Prettenhofer, Ron Weiss, Vincent Dubourg, Jake Vanderplas, Alexandre Passos, David Cournapeau, Matthieu Brucher, Matthieu Perrot, Édouard Duchesnay, JMLR 12, pp. 2825-2830, 2011. http://jmlr.csail.mit.edu/papers/v12/pedregosa11a.html
4. Travis E, Oliphant. A guide to NumPy, USA: Trelgol Publishing, (2006).
5. Shannon, C. E. (1948). A mathematical theory of communication. Bell system technical journal, 27(3), 379-423.
6. Press, W. H., Flannery, B. P., & Teukolsky, S. A. (1988). WT Vetterling WT. Numerical Recipes in C.
